# Synergistic effect of paired Cu(II) open metal sites for enhanced high-temperature hydrogen isotope separation

**DOI:** 10.1038/s41467-026-76556-7

**Published:** 2026-08-10

**Authors:** Fenglei Wang, Zhihai Fu, Xingwen Feng, Xiangyang Liu, Zining Wang, Chaofan Jiang, Wenting Liu, Yuchen Jiang, Yu Luo, Huimin Li, Qingyang Wang, Yuanhua Wang, Song Qin, Yongdong Jin, Chuanqin Xia, Lijian Ma

**Affiliations:** 1 College of Chemistry, Sichuan University, Chengdu, China; 2 Department of Radiochemistry (China Institute of Atomic Energy), Beijing, China; 3 Institute of Materials, China Academy of Engineering Physics, Mianyang, China; 4 College of Chemistry and Material Science, Sichuan Normal University, Chengdu, China

**Keywords:** Metal-organic frameworks, Nuclear chemistry

## Abstract

The selective separation of hydrogen isotopes under mild cryogenic conditions remains a formidable challenge due to their nearly identical physicochemical properties. Here, we report a dual strategy of pore topology design and paired Cu(II) open metal sites (OMS) synergistic engineering to amplify chemical affinity quantum sieving (CAQS). Among three tailored Cu(II)-MOFs, Cu-ATC exhibited exceptional performance, achieving a D_2_/H_2_ selectivity of 20 at 50 K (10 mbar) and 1.8 in breakthrough experiments at 77 K, demonstrating excellent H_2_/D_2_ separation performance. The ultramicroporous topology of Cu-ATC fixes a Cu···Cu distance of 5.98 Å within one-dimensional channels, while Jahn–Teller distortion induces axial elongation at each Cu(II) center, thereby enhancing the accessibility of the d_z_^2^ orbitals for interaction with hydrogen isotope molecules. This structural combination creates two closely spaced OMSs that enhance differential interactions with H_2_ and D_2_, thereby driving isotope separation via CAQS. The distinct binding strength is evidenced by in situ DRIFTS (v(H-H)/ v(D-D) red-shift of 203 cm^−1^/ 147 cm^−1^) and by DFT calculations showing stronger adsorption of H_2_ ( − 9.7 kJ mol^−1^) and D_2_ ( − 13.0 kJ mol^−1^). These microscopic differences account for the observed D_2_/H_2_ selectivity, highlighting the potential of paired OMSs engineering for CAQS-based isotope separation under mild cryogenic conditions.

## Abstract (web summary)

A dual strategy of pore topology and paired Cu(II) open metal sites amplifies chemical affinity quantum sieving, enabling high D_2_/H_2_ selectivity (20 at 50 K) in Cu-ATC. Ultramicropores and Jahn–Teller–elongated d_Z_^2^ orbitals create closely spaced sites that enhance differential isotope binding, confirmed by spectroscopy and DFT. This approach demonstrates a promising route for hydrogen isotope separation under mild cryogenic conditions.

## Introduction

Deuterium enrichment from hydrogen isotope mixtures represents a critical bottleneck in nuclear fusion fuel cycles. In current tokamak reactors, the utilization of D_2_ and T_2_ fuels is typically below 10%, with most discharged as waste streams, leading to tritium loss and the requirement for continuous deuterium enrichment to sustain the fuel cycle^1,2^. Despite decades of research, conventional separation technologies, including cryogenic distillation, thermal diffusion, and chromatographic methods, suffer from high energy consumption and low separation factors (~1.5), rendering them economically unsustainable for large-scale implementation^3^, underscoring the urgent need for new approaches that combine higher selectivity with lower energy demand. The intrinsic difficulty in hydrogen isotope separation lies in their nearly identical physicochemical properties, with the primary difference being a twofold mass difference. The heavier isotope (D_2_) has a shorter de Broglie wavelength and a lower zero-point energy, making it more readily adsorbed by microporous materials. This quantum effect can therefore be leveraged for effective isotope separation.

Metal-organic frameworks (MOFs) have emerged as promising platforms for hydrogen isotope separation by exploiting quantum sieving effects arising from zero-point energy differences^4–7^. Kinetic quantum sieving (KQS) can deliver remarkable selectivity—D_2_/H_2_ = 41.4 at 20 K^8^—through diffusion discrimination in confined pores, but this requires impractically low temperatures. To extend applicability, chemical-affinity quantum sieving (CAQS) was introduced^9^, relying on Kubas-type *σ* donation and *π* back-donation between H_2_/D_2_ and open metal sites^10–17^. At 77 K, however, achieving high selectivity (>10) remains challenging, particularly regarding stability and capacity for practical application. Building on this, researchers have further pursued new explorations based on the CAQS separation mechanism. Imidazole-modified MOF-74 attained a selectivity of 26 at 77 K, demonstrating the potential of KQS-CAQS synergy^18^. In addition, Cu(I)-exchanged ZSM-5 reached a selectivity of 24.9 at 100 K, illustrating the feasibility of introducing open metal sites (OMSs) in porous materials^19^. However, these studies also revealed several shortcomings, including the instability of Cu(I) sites, the complexity of post-synthetic modification, and the rapid loss of selectivity at temperature rises above 77 K. The central challenge, therefore, remains to establish isotope–metal interactions that are both strong and durable, capable of sustaining high selectivity under practical cryogenic conditions without compromising framework integrity.

**Fig. 1:**
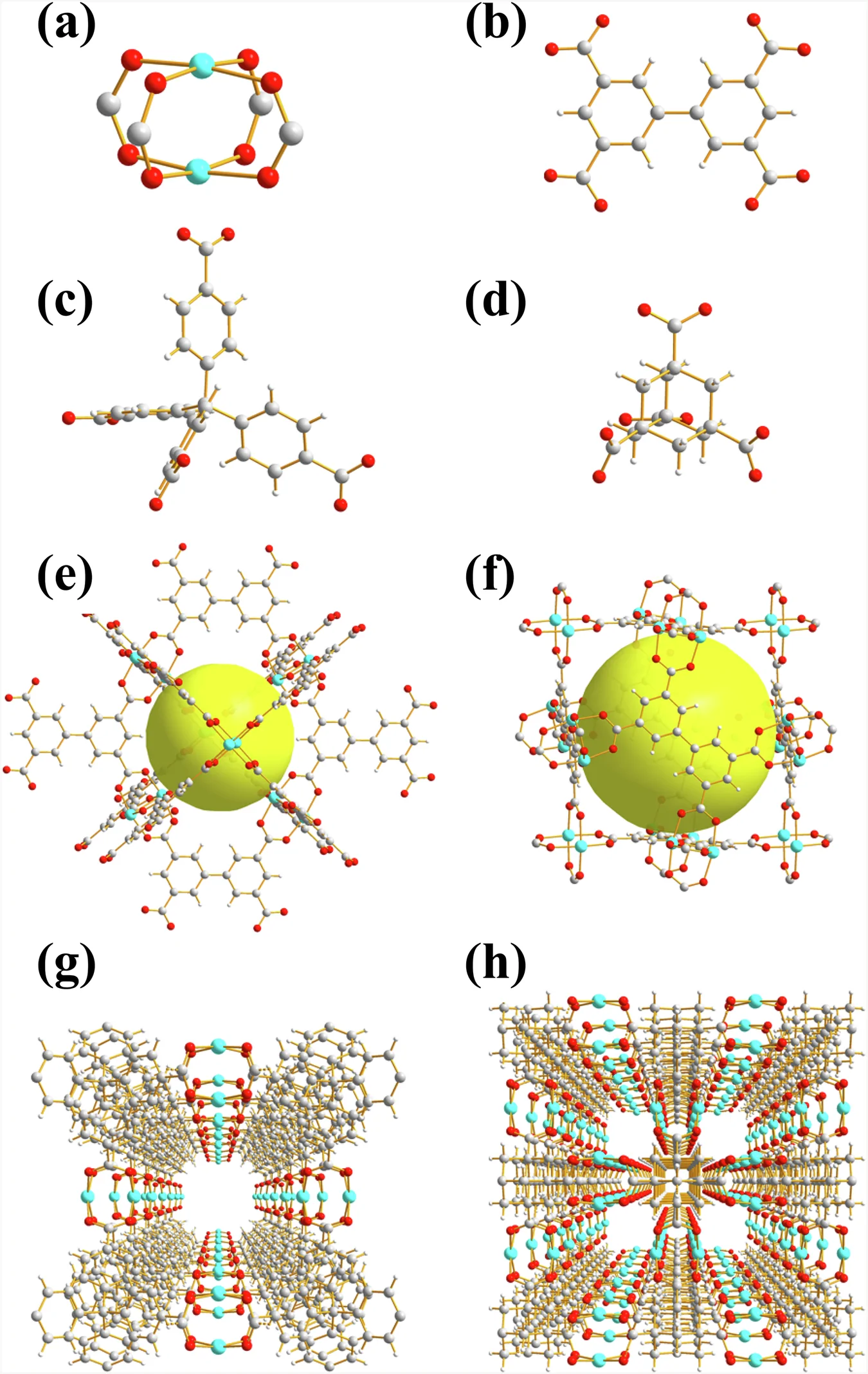
MOF Structure. Illustrated structure of **a** Cu_2_(CO_2_)_4_ units. **b** 3,3′,5,5′-biphenyltetracarboxylate. **c** 4,4′,4′′,4′′′-methanetetrayltetrabenzoate. **d** 1,3,5,7-adamantanetetracarboxylate. **e**, **f** Schematic illustrations of two cage-like structures in Cu-bptc. Three-dimensional structural schematic of **g** Cu-mbtc and **h** Cu-ATC. (Atom colors: C: gray, O: red, Cu: cyan, H: white, and the yellow sphere represents the largest sphere that would occupy the cavity without contacting the interior van der Waals surface with diameters of 8 Å (**e**) and 10 Å (**f**), respectively).

Among MOF architectures, Cu(II)-based paddlewheel frameworks are particularly attractive because their robust [Cu_2_(O_2_CR)_4_] secondary building units (SBUs, Fig. 1c) allow facile generation of open metal sites upon activation and systematic modulation of pore environments. In addition, the Jahn–Teller distortion intrinsic to Cu(II) enhances the accessibility of *d*_z_^2^ orbitals for guest–molecule interactions^20,21^, offering potential advantages for isotope binding. Despite these features, benchmark Cu-BTC (HKUST-1) delivers only modest performance, with a D_2_/H_2_ selectivity of 1.3 at 77 K^22–27^, as its large ~1 nm cages permit molecules to bypass OMSs and diminish the CAQS effect. Attempts to improve performance by reducing Cu(II) to Cu(I) temporarily boosted selectivity to 37.9 at 30 K^28^, but this advantage quickly disappeared above 40 K because the reduction disrupted *d*_z_^2^ orbital alignment and undermined framework stability. A similar issue was observed in FJI-Y11^29^, where DFT calculations revealed that adsorption occurred near oxygen atoms rather than Cu centers, as the *d*_z_^2^ orbitals were geometrically misaligned with the pore channels, limiting effective interaction with hydrogen molecules and resulting in low selectivity (*α* ≈ 1.4).

These observations show that OMS density alone is not sufficient, and that isotope separation critically depends on how pore confinement and orbital orientation cooperate. A promising direction is therefore to design frameworks with ultramicroporous channels that enforce close contact between isotopes and appropriately oriented, Jahn–Teller-stabilized Cu(II) sites, while preserving the structural integrity of the paddlewheel motif.

Guided by this rationale, we synthesized three tetracarboxylate Cu(II) MOFs with distinct topologies, including cage-type Cu-bptc^30^, wide-channel Cu-mbtc (~10 Å)^31^, and ultramicroporous Cu-ATC (~5.98 Å)^32^, to examine how pore architecture affects isotope separation. Comparative evaluation showed that Cu-bptc and Cu-mbtc displayed only limited selectivity, indicating that pore confinement alone cannot account for high performance. In contrast, Cu-ATC exhibited outstanding separation, achieving a D_2_/H_2_ selectivity of 20 at 50 K and 1.8 in breakthrough experiments at 77 K, demonstrating excellent H_2_/D_2_ separation performance among Cu(II)-MOFs. Subsequent analysis revealed that its ultramicroporous channels not only constrain molecular motion but also position isotopes within reach of two closely spaced paddlewheel Cu(II) sites, where accessible *d*_z_^2^ orbitals enable cooperative dual-site interactions that markedly amplify the CAQS effect. These findings establish Cu-ATC as a benchmark system and highlight a generalizable design principle in which pore topology and site-specific electronic structure act in concert to realize practical hydrogen isotope separation under mild cryogenic conditions.

## Results and discussion

### Structure characterization

The structures of the three Cu(II)-based MOFs were characterized by powder X-ray diffraction (PXRD) and nitrogen adsorption-desorption experiments, with detailed results provided in the Supplementary Information. The PXRD patterns confirmed that the crystal structures of these materials aligned with literature reports, indicating successful synthesis and high purity (Figs. S1–3). The nitrogen and Argon adsorption isotherms exhibited IUPAC Type I characteristics for all three MOFs, verifying their microporous nature (Figs. S4–9). Pore size distributions (Figs. S10–12), calculated using nonlocal density functional theory (NLDFT), revealed distinct topologies: Cu-ATC and Cu-bptc possess narrow ultramicropore (5.6 and 5.8 Å, respectively), whereas Cu-mbtc showed a larger pore size (10.0 Å). This variation reflects the designed optimization from cage-like Cu-bptc to one-dimensional channels in Cu-mbtc and Cu-ATC (Fig. 1). Brunauer–Emmett–Teller (BET) surface areas were 606 m^2^ g^−1^ for Cu-ATC, 1811 m^2^ g^−1^ for Cu-bptc, and 498 m^2^ g^−1^ for Cu-mbtc (Figs. S4–6). These values correlate with framework compactness: Cu-bptc’s high surface area arises from its interconnected cage network, whereas Cu-ATC’s lower value stems from denser ultramicroporous channels. Such topological tuning establishes the structural basis for subsequent hydrogen isotope adsorption studies, consistent with the dual strategy of pore topology modulation and bimetallic synergy.

### Adsorption properties of H_2_ and D_2_

**Fig. 2:**
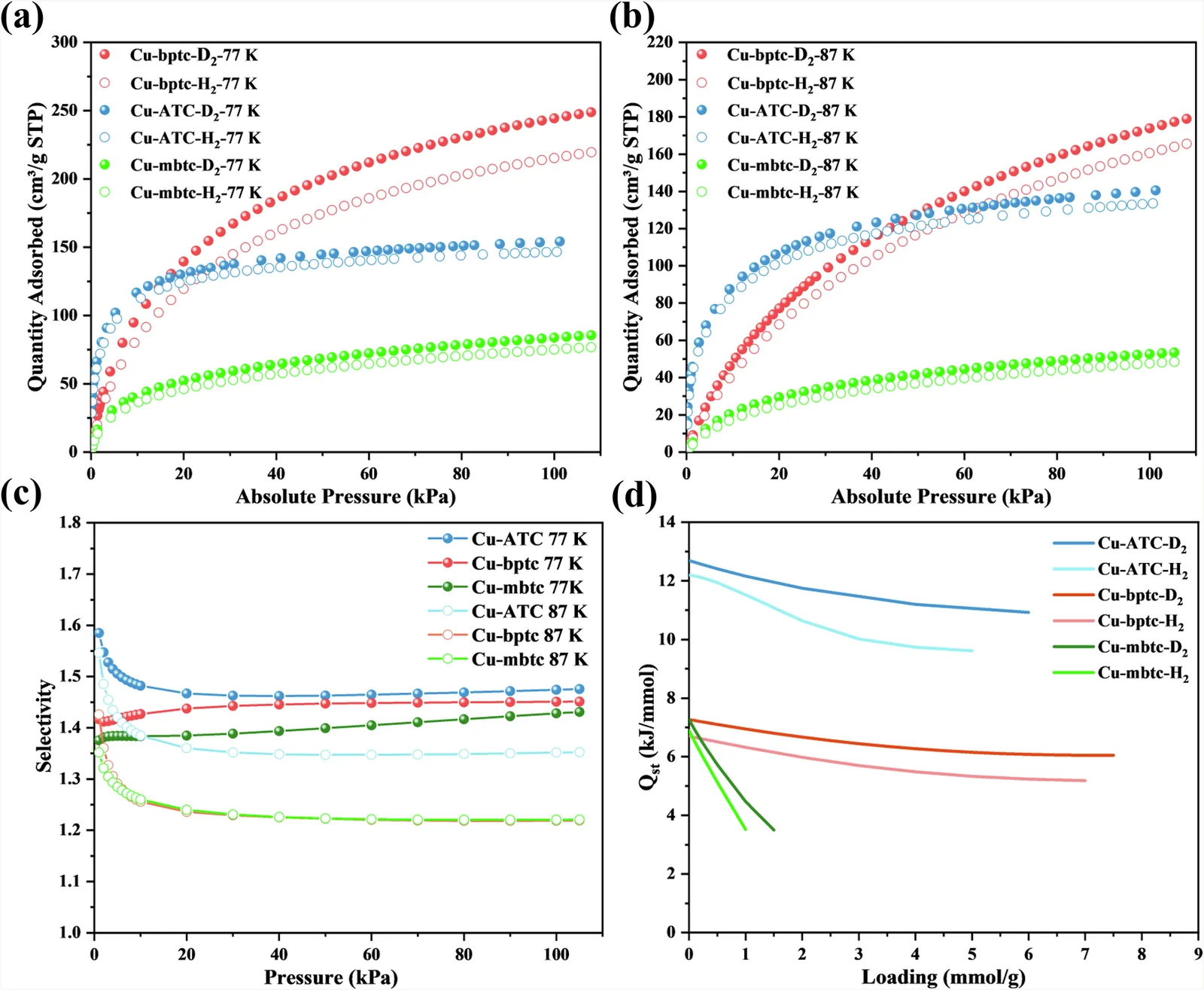
H2/D2 single-component adsorption. Single-component H_2_ and D_2_ adsorption isotherms of Cu-ATC (blue), Cu-bptc (red), and Cu-mbtc (green) at 77 K (**a**) and 87 K (**b**). IAST selectivity of Cu-ATC, Cu-bptc, and Cu-mbtc for 1:1 D_2_/H_2_ mixture at 77 and 87 K (**c**). The isosteric heat of D_2_ and H_2_ for Cu-ATC, Cu-bptc, and Cu-mbtc (**d**).

Single-component H_2_ and D_2_ adsorption isotherms for the three MOFs were measured at 77 and 87 K (Fig. 2a, b). At 77 K and 101 kPa, Cu-bptc exhibited high uptake capacities (*q*): 249 cm^3^ g^−1^ for D_2_ and 220 cm^3^ g^−1^ for H_2_, outperforming many reported MOF-based hydrogen storage materials. In contrast, Cu-ATC showed lower capacities (154 cm^3^ g^−1^ for D_2_ and 147 cm^3^ g^−1^ for H_2_), attributable to its narrow one-dimensional channels (~5.6 Å) limiting accessible volume, while Cu-bptc’s cage-like structure (~8–10 Å) offers larger voids. Cu-mbtc displayed even lower uptakes (86 cm^3^ g^−1^ for D_2_ and 77 cm^3^ g^−1^ for H_2_); this result was attributed to its larger channel size (~10 Å) relative to Cu-ATC, which provided a less favorable confinement environment for hydrogen adsorption in these topologically similar frameworks.

The isotherm steepness in the low-pressure region (<10 kPa at 77 K) followed the order Cu-ATC > Cu-bptc > Cu-mbtc (Fig. 2a), reflecting stronger gas-framework interactions and faster adsorption kinetics in Cu-ATC. At 87 K (Fig. 2b), Cu-ATC’s capacities decreased by only 8–9% (at 101 kPa), whereas Cu-bptc and Cu-mbtc decreased by 25–28% and 36–37%, respectively, underscoring Cu-ATC’s enhanced adsorption stability at elevated temperatures.

Ideal adsorbed solution theory (IAST) selectivity for an equimolar D_2_/H_2_ mixture, calculated using a dual-site Langmuir–Freundlich model, was 1.50 for Cu-ATC at 77 K, 101 kPa, surpassing those of Cu-bptc (1.45) and Cu-mbtc (1.43). At 87 K, 101 kPa, the selectivity of Cu-ATC remained 1.35, higher than that of Cu-bptc and Cu-mbtc (1.22). These results indicate Cu-ATC maintains superior D_2_/H_2_ separation performance under mild conditions.

Isosteric heats of adsorption (*Q*_st_), derived via the virial method (Fig. 2d), further elucidated the mechanism. Cu-ATC’s zero-coverage *Q*_st_ values were 12.47 kJ mol^−1^ for D_2_ and 12.09 kJ mol^−1^ for H_2_—the highest among copper paddlewheel MOFs—exceeding those of Cu-bptc (7.26/6.70 kJ mol^−1^), Cu-mbtc (7.26/6.88 kJ mol^−1^), FJI-Y11 (7.88/7.13 kJ mol^−1^), and HKUST-1 (7.32/6.41 kJ mol^−1^). Cu-ATC also outperformed Co-MOF-74 (12.1/11.0 kJ mol^−1^) and Zn-MOF-74 (9.2/8.3 kJ mol^−1^), being only slightly below Ni-MOF-74 (13.7/12.1 kJ mol^−1^). The maximum D_2_-H_2_ *Q*_st_ difference of 1.4 kJ mol^−1^ ranks among the highest for copper paddlewheel MOFs, indicating that paired OMSs and ultramicropores synergistically enhance isotope affinity differences, bolstering the CAQS mechanism.

### In situ diffuse reflectance infrared Fourier transform spectroscopy (DRIFTS)

**Fig. 3:**
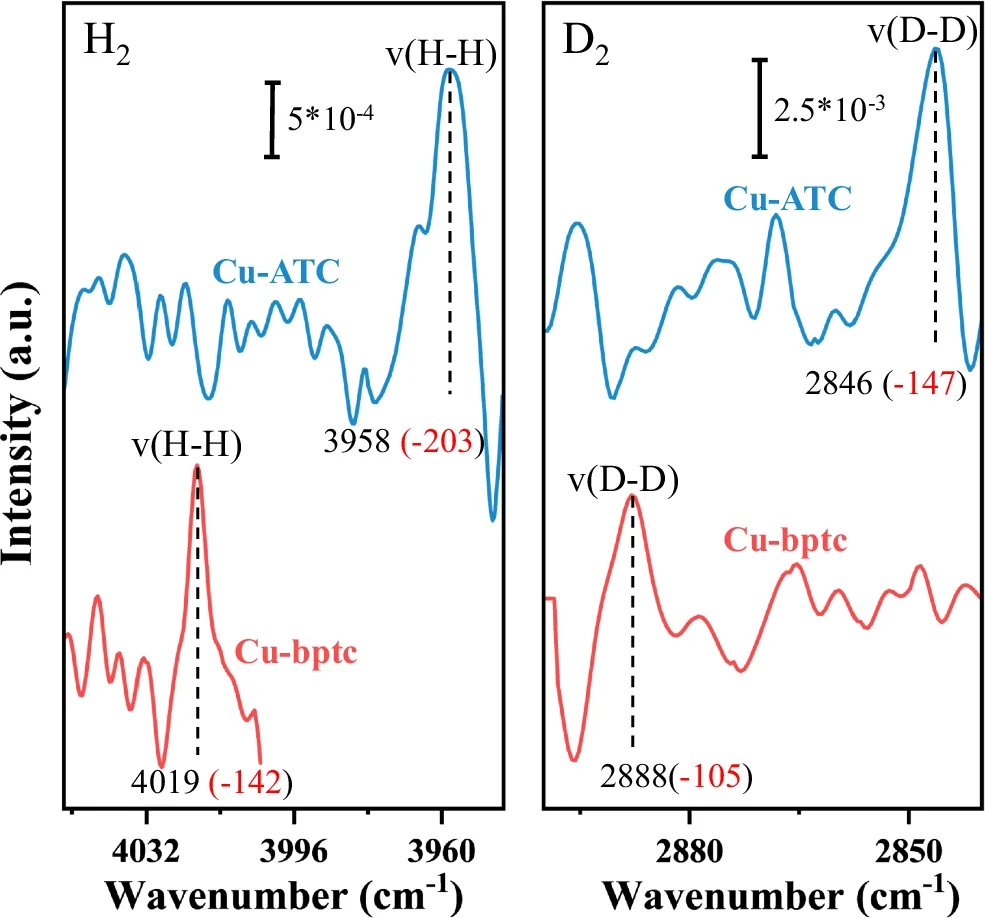
In situ DRIFT spectra of adsorbed H_2_ and D_2_ in the activated Cu-ATC (blue)/Cu-bptc (red). Gas was loaded at 130 K/1 atm.

To probe the H_2_/D_2_ adsorption mechanism in the MOFs, in situ DRIFTS measurements of H_2_/D_2_ adsorption were conducted at 130 K (Fig. 3). Distinct adsorption bands of H_2_ and D_2_ were observed for Cu-ATC and Cu-bptc, appearing at 3958/4019 and 2846/2888 cm^−1^, respectively. In contrast, no detectable signals for adsorbed H_2_ or D_2_ were observed on Cu-mbtc, which is consistent with its weaker confinement effect and less favorable adsorption environment for hydrogen isotopes. The H_2_ bands at 3958/4019 cm^−1^ fall within the typical range reported for H_2_ adsorbed at the open metal sites, and can be assigned to Cu-(H_2_) adducts.

Relative to gaseous H_2_ at 4161 cm^−1^, the H–H stretching vibration (v(H-H)) is red-shifted by 203 cm^−1^ for Cu-ATC and 142 cm^−1^ for Cu-bptc, indicating a stronger interaction in Cu-ATC. For D_2_ adsorption, compared to gaseous D_2_ at 2993 cm^−1^, the corresponding red shifts are 147 cm^−1^ for Cu-ATC and 105 cm^−1^ for Cu-bptc, following the expected isotopic trend. Although both Cu-ATC and Cu-bptc feature identical paddlewheel-type Cu(II) OMS and comparable pore apertures, the more pronounced red shifts observed for Cu-ATC suggest a stronger adsorption environment, which is consistent with the influence of Cu-ATC’s ultramicroporous one-dimensional channels (~5.98 Å) and closely spaced Cu sites.

### Cryogenic thermal desorption spectroscopy (TDS): adsorption sites and isotope preferential binding

**Fig. 4:**
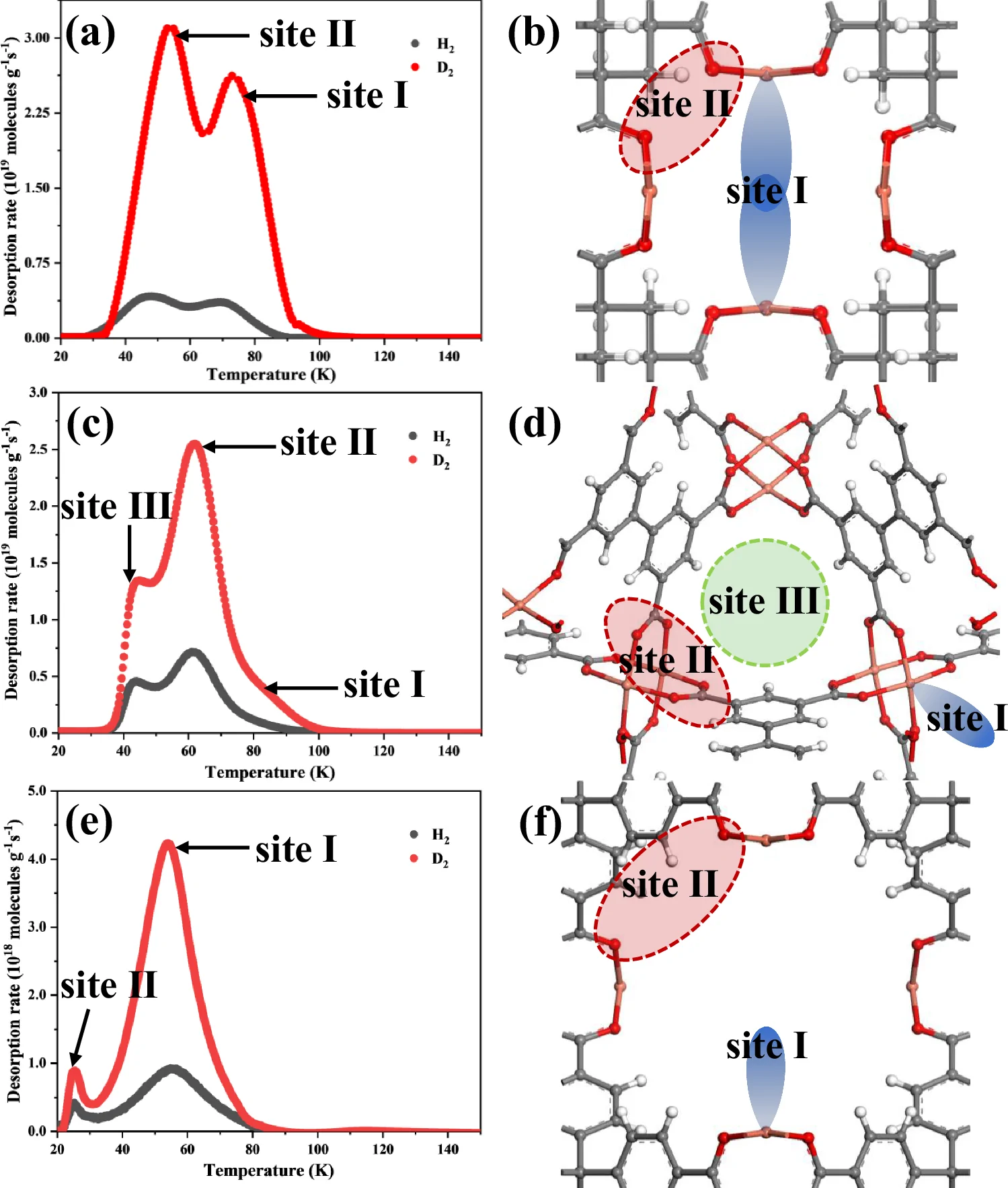
TDS measurement. Thermal desorption spectra and corresponding adsorption sites of Cu-ATC (**a**, **b**), Cu-bptc (**c**, **d**), and Cu-mbtc (**e**, **f**). The samples were exposed to 1:1 D_2_/H_2_ mixture gas at 26 K for 10 min and then evacuated before heating at a rate of 6 K/min.

To identify adsorption sites in the three MOFs and their affinity for H_2_/D_2_, TDS measurements were conducted. Samples were exposed to an equimolar H_2_/D_2_ mixture (10 mbar) at 26 K for 10 min, evacuated to 20 K, and heated at 0.1 K s^−1^ (6 K min^−1^) over 20–150 K (Fig. 4). Cu-ATC’s TDS spectrum revealed two desorption peaks (Fig. 4a): a higher-temperature peak assigned to OMSs (Site I) and a lower-temperature peak associated with framework oxygen atoms (Site II). D_2_ peaks shifted to higher temperatures than H_2_, indicating preferential D_2_ binding at OMSs due to lower zero-point energy, thereby enhancing CAQS, consistent with the *Q*_st_ difference (1.4 kJ mol^−1^) and in situ DRIFTS redshift. Cu-bptc showed three peaks (Fig. 4c): OMSs (I), oxygen atoms (II), and small pores (III), but the difference in adsorption capacity between D_2_ and H_2_ was relatively small, reflecting diluted site-specific interactions in its cage-like topology. Cu-mbtc exhibited two peaks (Fig. 4e): OMSs (I) and large pores (II), with the weakest D_2_ preference, attributed to its larger channel size (~10 Å) diminishing interactions. These TDS results quantify topology-dependent site diversity: Cu-ATC’s ultramicroporous channels amplify OMS preference for D_2_ (largest peak shift), providing the basis for its high selectivity and distinguishing it from conventional copper MOFs.

### Separation performance

**Fig. 5:**
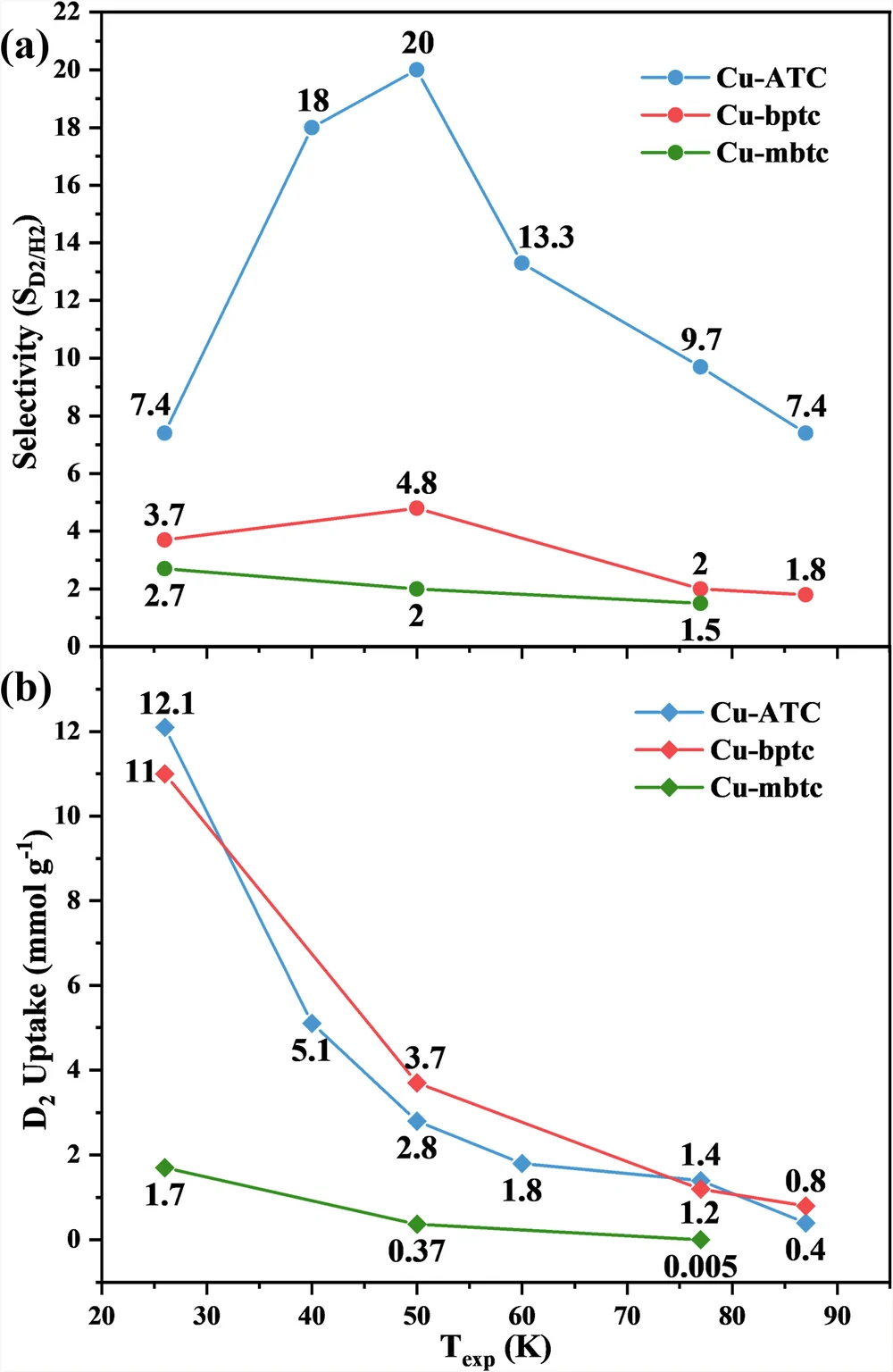
Adsorptive separation for an equimolar D_2_/H_2_ isotope mixture. **a** Selectivity for D_2_ as compared to H_2_ and **b** D_2_ uptake of Cu-ATC (blue), Cu-bptc (red), and Cu-mbtc (green) as a function of *T*_exp_.

**Fig. 6:**
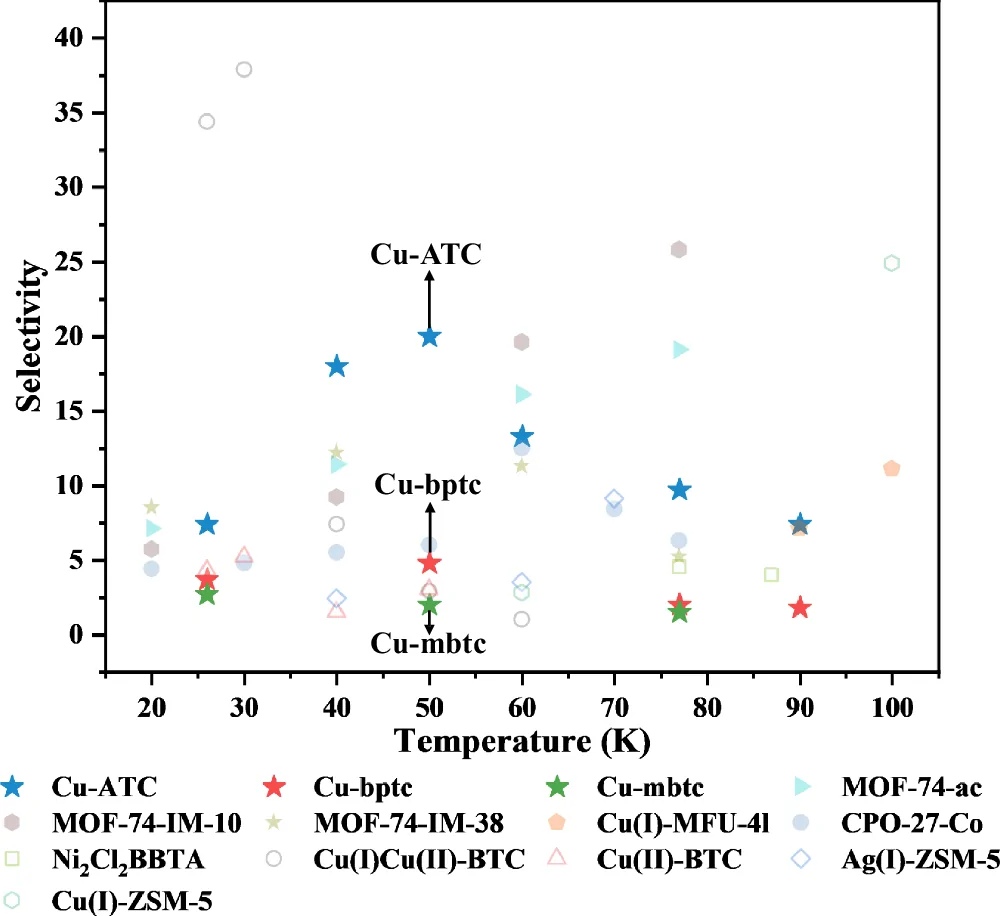
Comparison of D2/H2 selectivities. Comparison of the D_2_/H_2_ selectivity between the results in the literature^18,19,28,33–36^ and our work.

To assess the practical impact of the MOF topologies on separation, samples were exposed to an equimolar H_2_/D_2_ mixture (10 mbar) at varying temperatures (26, 40, 50, 60, 77, and 87 K), with selectivity (*S*_D2/H2_ = D_2_/H_2_ desorption area ratio) evaluated via TDS (Fig. 5 and Supplementary Information Figs. S13–15). At 26 K, all adsorption sites (strong and weak) were occupied, and the combined KQS and CAQS effects yielded a high D_2_ preference. Above 50 K, adsorption was confined to OMSs, yet CAQS continued to favor D_2_. Cu-bptc and Cu-mbtc selectivities declined sharply with temperature, reflecting their weak OMS affinity, whereas Cu-ATC exhibited relatively high D_2_/H_2_ selectivities of 20 at 50 K and 7.4 at 87 K. The selectivity of Cu-ATC first increased from 26 to 50 K and then decreased above 50 K, indicating a non-monotonic dependence on exposure temperature. This phenomenon can be understood as arising from the combined effects of weak binding sites (WBSs), strong binding sites (SBSs), and molecular thermal motion^18^. Below 50 K, WBSs, such as oxygen atoms, also contributed significantly to D_2_/H_2_ uptake. Because these WBSs showed only limited D_2_/H_2_ discrimination, their contribution led to relatively low selectivity. As the exposure temperature (*T*_exp_) increased toward 50 K, adsorption at WBSs was gradually reduced, while the SBSs, such as paired Cu(II) OMSs, remained capable of preferentially adsorbing D_2_ over H_2_. As a result, the D_2_/H_2_ selectivity reached its maximum at 50 K. When *T*_exp_ increased above 50 K, molecular thermal motion became stronger, and the preferential adsorption of D_2_ at the paired Cu(II) OMSs was weakened; consequently, the D_2_/H_2_ selectivity declined. Over the investigated *T*_exp_ range, the selectivity of Cu-ATC was consistently higher than that of Cu-bptc and Cu-mbtc. This performance originates from its ultramicroporous channels (~5.98 Å) and paired OMS synergy enhancing isotope affinity differences, consistent with *Q*_st_ and DRIFTS results. These findings highlight the potential of Cu-ATC for H_2_/D_2_ separation at liquid-nitrogen-accessible temperatures (Fig. 6 and Table S1), while further evaluation under practical process conditions will be required to assess its applicability.

### Dynamic column breakthrough experiments

**Fig. 7:**
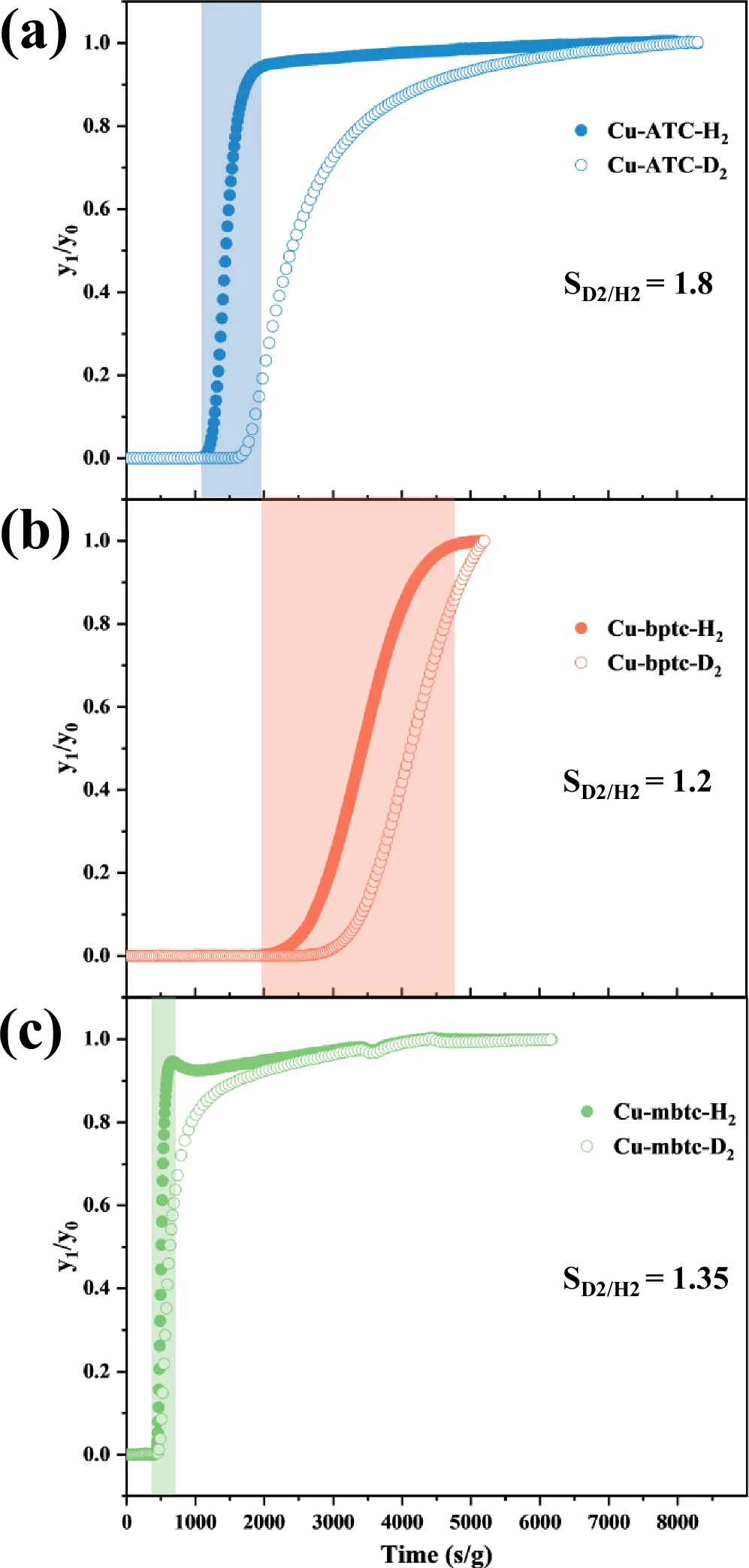
H2/D2 mixed-gas breakthrough curves. The dynamic breakthrough curves of Cu-ATC (**a**), Cu-bptc (**b**), and Cu-mbtc (**c**) at 77 K for the mixed gases of H_2_/D_2_/Ne (10/10/80 vol.%).

To simulate industrial H_2_/D_2_ separation, dynamic column breakthrough experiments were performed using an H_2_/D_2_/Ne mixture (10/10/80 vol.%) at a flow rate of 5 mL min^−1^, 77 K, and 100 kPa (Fig. 7). In all materials, H_2_ broke through first (reflecting its lower binding affinity), whereas D_2_ was retained longer. Cu-mbtc showed nearly simultaneous H_2_/D_2_ breakthrough (Fig. 7c), yielding a low selectivity of 1.35, due to channel dilation that weakens isotope discrimination separation. In contrast, Cu-ATC exhibited an H_2_ breakthrough time of 823 s g^−1^ (Fig. 7a), much shorter than Cu-bptc’s 2593 s g^−1^ (Fig. 7b), reflecting accelerated displacement kinetics in its ultramicroporous framework. For D_2_, the breakthrough time was 5400 s g^−1^ in Cu-ATC, significantly longer than that of Cu-bptc (2483 s g^−1^), demonstrating enhanced retention. Curve integration yielded dynamic parameters: Cu-ATC showed a D_2_ capacity of 1.04 mmol g^−1^ with a selectivity of 1.8, outperforming Cu-bptc (1.2) and Cu-mbtc (1.35). Although dynamic values were lower than those from static isotherms, owing to incomplete equilibration under flow, the superior dynamic selectivity and TDS selectivity (20 at 50 K) of Cu-ATC suggested its potential for further applied research.

### Adsorption mechanism study

**Fig. 8:**
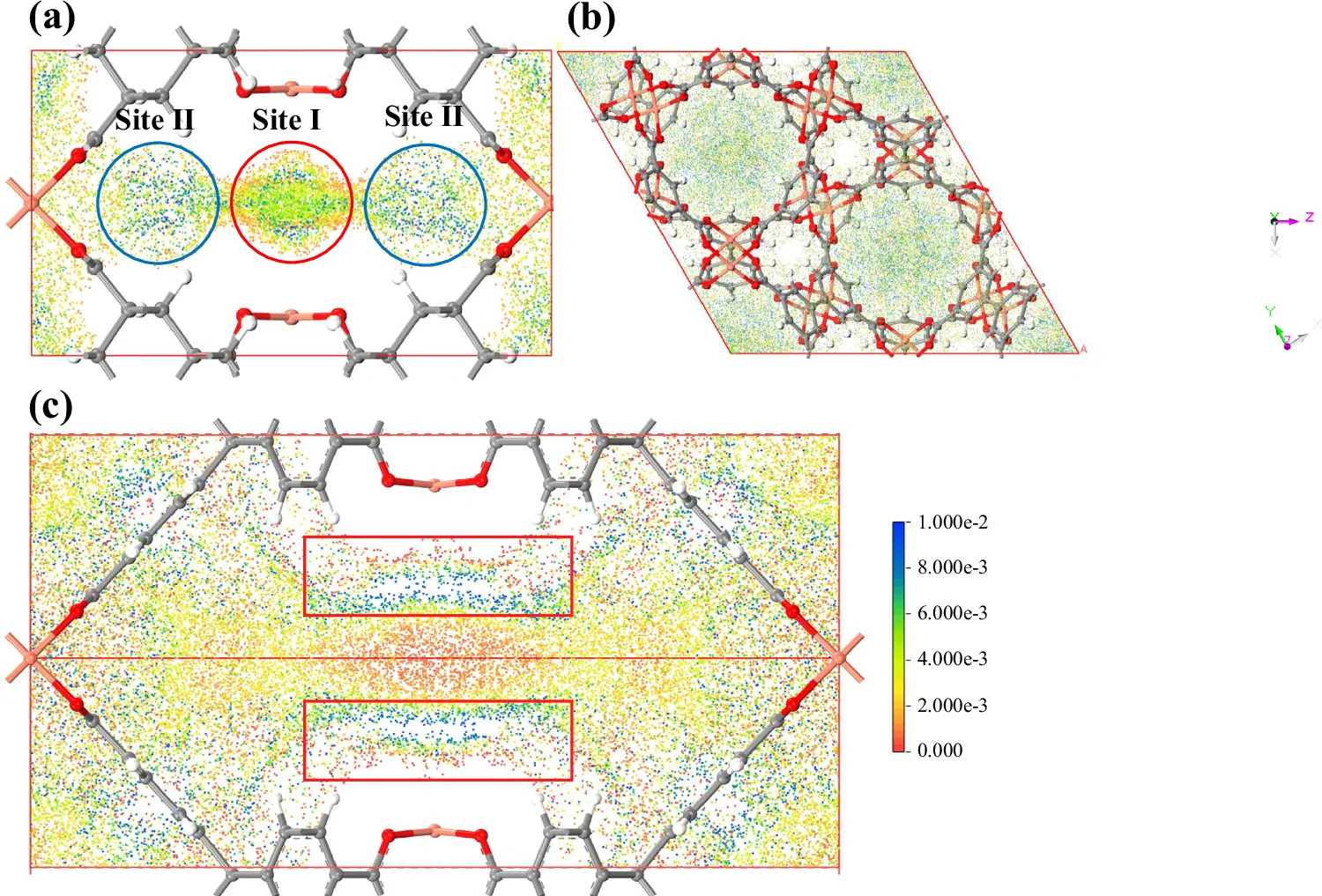
GCMC calculations. The probability distribution density diagram of H_2_ molecules adsorption sites around the Cu-ATC (**a**), Cu-bptc (**b**), and Cu-mbtc (**c**). Color code: C, gray; O, red; Cu, orange.

To elucidate the hydrogen adsorption and separation mechanisms in the three MOFs, Grand Canonical Monte Carlo (GCMC) simulations were conducted in Materials Studio at 77 K 101 kPa and constant pressure and probability density maps of H_2_ were obtained as shown in Fig. 8, the results showed two primary adsorption sites in Cu-ATC (Fig. 8a): (I) near copper paddlewheel OMSs and (II) proximal to oxygen atoms in the clusters. Adsorption at Site II was attributed to the compact structure, which facilitated significant interactions between H_2_ and oxygen atoms within the framework. In contrast, Cu-bptc (Fig. 8b) exhibited uniform H_2_ distribution across cage-like cavities, lacking OMS localization; its spacious voids (~8–10 Å) dilute interactions, weakening the CAQS effect. Cu-mbtc (Fig. 8c) showed a preference for wall-bound OMSs due to the excessively large pore size (~10 Å). The above three patterns confirm topological control: Cu-ATC’s confined channels (~5.98 Å) enforce OMS engagements, boosting CAQS.

**Fig. 9:**
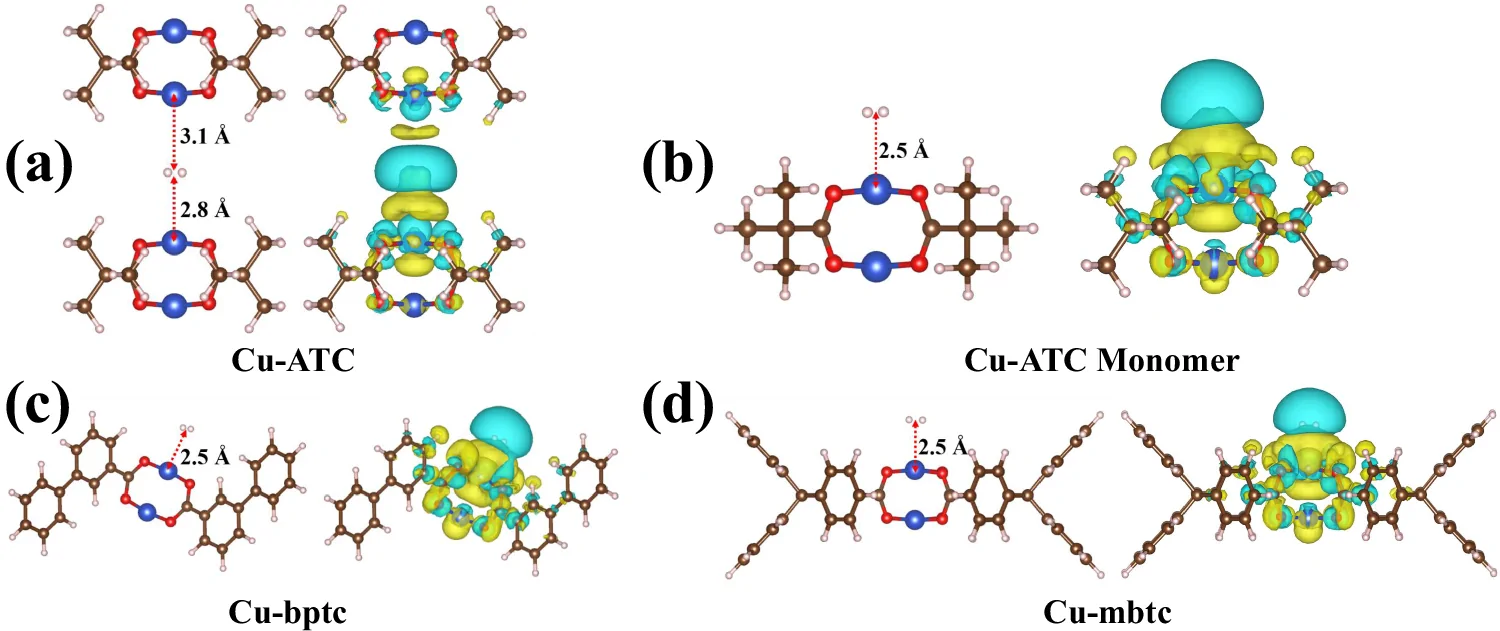
DFT calculations. Structure of finite model and 3D charge density difference of **a** Cu-ATC, **b** Cu-ATC monomer, **c** Cu-bptc, and **d** Cu-mbtc as optimized by DFT and corresponding equilibrium position of the hydrogen molecule adsorbed at the Cu(Ⅱ) atom. The yellow zone and cyan zone denote the charge accumulation and depletion. (blue, copper; red, oxygen; tan, carbon; and pink, hydrogen).

Periodic density functional theory (DFT) calculations, based on GCMC-identified sites, assessed H_2_/D_2_ adsorption energies (Fig. 9). The calculated H_2_/D_2_ adsorption energies at the primary adsorption site I (between adjacent paddlewheels, shown in Fig. 9a) of Cu-ATC were −9.07 and −13.00 kJ mol^−1^, respectively, which are markedly stronger than those of Cu-bptc (H_2_: −6.42 kJ mol^−1^, D_2_: −9.61 kJ mol^−1^) and Cu-mbtc (H_2_: −6.90 kJ mol^−1^, D_2_: −10.12 kJ mol^−1^). To distinguish the paired-site effect from a single-site effect, we further calculated the adsorption energies for H_2_/D_2_ on an isolated single paddlewheel-type Cu(II) site of Cu-ATC (Fig. 9b). The resulting adsorption energies (H_2_: −6.16 kJ mol^−1^, D_2_: −9.62 kJ mol^−1^) were comparable to those of Cu-bptc and Cu-mbtc. More importantly, the calculated vibrational frequencies of adsorbed H_2_/D_2_ in the three MOFs (shown in Table S2) were in good agreement with the experimental values. These computational results suggest that the enhanced affinity of Cu-ATC for hydrogen isotopes cannot be attributed to an individual Cu site alone, but is instead associated with the closely spaced Cu···Cu arrangement (5.98 Å). Consistent with this interpretation, the charge density difference maps (Fig. 9) reveal electron redistribution involving the adsorbed hydrogen isotope molecule and the two closely spaced Cu open metal sites in Cu-ATC, while the corresponding single-site model as well as Cu-bptc and Cu-mbtc do not exhibit the same dual-site interaction feature. Taken together, these results support the conclusion that the enhanced adsorption strength of Cu-ATC arises from a cooperative effect associated with the paired Cu OMSs.

In summary, we demonstrate that pore topology modulation combined with paired Cu(II) OMS synergistic engineering enables Cu-ATC, a paddlewheel-type MOF with one-dimensional ultramicroporous channels, to achieve record hydrogen isotope separation performance. Cu-ATC exhibits steep adsorption isotherms at low pressures and a TDS selectivity of 20 at 50 K, representing a relatively high value among reported Cu(II)-MOFs, although these literature data were obtained under non-identical experimental conditions. Importantly, at 77 K, its dynamic breakthrough selectivity of 1.8 is maintained, arising from synergistic interactions between paired Cu sites and ~5.98 Å channels that amplify CAQS (Δ*Q*_st_ = 1.4 kJ mol^−1^). These findings establish the structural principles by which confinement and OMS arrangement cooperate to determine isotope separation, providing a general framework for designing efficient MOFs under mild conditions and advancing deuterium enrichment technologies.

## Methods

### Synthesis of Cu_2_(ATC)(H_2_O)_2_·5H_2_O

The Cu_2_(ATC)(H_2_O)_2_·5H_2_O was synthesized according to the method from literature with slight modification^32^. A mixture of H_4_ATC (0.035 g, 0.11 mmol) and Cu(NO_3_)_2_·2.5H_2_O (0.077 g, 0.33 mmol) in 3 mL 1 × 10 M^−3^ aqueous NaOH, was heated to 100 °C to get the clear blue solution. The aqueous solution was then heated to 200 °C for 18 h to give green crystals of ATC-Cu along with tiny amounts of crystalline H_4_ATC. The solid was filtered and then washed with hot water several times and then get the pure Cu_2_(ATC)(H_2_O)_2_·5H_2_O crystal.

### Preparation of anhydrous Cu-ATC

The crystals of Cu_2_(ATC)(H_2_O)_2_·5H_2_O were soaked in anhydrous methanol for 2 days with fresh methanol 5 times. Then, the crystals were vacuumed at room temperature till the pressure below to 3  μmHg; the temperature was increased to 120 °C and vacuum for 5 h. After the pressure decreasing to 3  μmHg, the temperature was increased to 190 °C and vacuum for 12 h. Consequently, the dark purple crystal of anhydrous Cu-ATC was obtained.

### Synthesis of Cu_2_(bptc)(H_2_O)_2_·3DMF·H_2_O

The Cu_2_(bptc)(H_2_O)_2_·3DMF·H_2_O was synthesized according to the method from literature with slight modification^30^. The solvothermal reaction of 3,3′,5,5′-biphenyltetracarboxylic acid (H_4_bptc; 0.025 g, 0.076 mmol) and Cu(NO_3_)_2_·2.5H_2_O (0.052 g, 0.22 mmol) in DMF/ethanol/H_2_O (3:3:2 v/v/v) at 65 °C for 24 h gave green, blockshaped crystals.

### Preparation of anhydrous Cu-bptc

The crystals of Cu_2_(bptc)(H_2_O)_2_·3DMF·H_2_O were soaked in acetone for 3 days with fresh acetone 3 times to exchange the DMF guests. Then, the crystals were vacuumed at room temperature till the pressure below to 3 μmHg, the temperature was increased to 120 °C and vacuum for 12 h. Consequently, the dark blue crystals of anhydrous Cu-bptc were obtained.

### Synthesis of Cu_2_(mbtc)(H_2_O)_2_·6DEF·2H_2_O

The Cu_2_(mbtc)(H_2_O)_2_·6DEF·2H_2_O was synthesized according to the method from literature with slight modification^31^. A mixture of H_4_mbtc (0.015 g, 0.030 mmol) and Cu(NO_3_)_2_·2.5H_2_O (0.03 g, 0.13 mmol) in DEF/H_2_O (4.5 mL/1.5 mL) mixed solvents was placed in a screw-capped vial. After addition of several drops of HCl (3 M, aq.), the vial was capped and placed in an oven at 80 °C for 1 day. Blue crystals of Cu_2_(mbtc)(H_2_O)_2_·6DEF·2H_2_O were collected by filtration.

### Preparation of anhydrous Cu-mbtc

The crystals of Cu_2_(mbtc)(H_2_O)_2_·6DEF·2H_2_O were washed with MeOH three times over a 12 h period, and then washed with CH_2_Cl_2_ three times. The resulting dark blue crystals were washed with cyclohexane several times and then soaked in cyclohexane overnight. About 1 mL of benzene was left in the sample cell, and the sample cell was then frozen at 0 °C. After three freeze-thaw cycles, the sample cell was placed in an ice/H_2_O bath and evacuated under a dynamic vacuum for 24 h. The ice/H_2_O bath was removed, and the sample was kept under vacuum at room temperature for another 24 h, and then heated under vacuum at 60 °C for 16 h. Consequently, the blue crystals of anhydrous Cu-mbtc were obtained.

### Reporting summary

Further information on research design is available in the Nature Portfolio Reporting Summary linked to this article.

## Supplementary information

Supplementary Information

Transparent Peer Review File

Reporting Summary

## Source data

Source Data

## Data Availability

All data involved in this work are included in this article and the corresponding Supplementary Information. Source data are provided in this paper. Source data are provided with this paper.
