# Supplementary material for: Synergistic effect of paired Cu(II) open metal sites for enhanced high-temperature hydrogen isotope separation
Source: Nat Commun. 2026 Aug 10;17:9585. doi: 10.1038/s41467-026-76556-7 (PMC13550398; doi:10.1038/s41467-026-76556-7)
Supplement: Supplementary file 1 [file 41467_2026_76556_MOESM1_ESM.pdf]

Supplementary Information

Synergistic Effect of Paired Cu(II) Open Metal Sites for Enhanced High-Temperature Hydrogen Isotope Separation

Fenglei Wang[1], Zhihai Fu[2], Xingwen Feng[3], Xiangyang Liu[4], Zining Wang[1], Chaofan Jiang[1], Wenting Liu[1], Yuchen Jiang[1], Yu Luo[1], Huimin Li[1], Qingyang Wang[1], Yuanhua Wang[1], Song Qin[1], Yongdong Jin[1], Chuanqin Xia[1,], Lijian Ma[1,*]*

Experimental Section

Materials

1, 3, 5, 7-adamantanetetracarboxylic acid (H_4ATC), 3,3′,5,5′-biphenyltetracarboxylic acid (H_4bptc), methanetetra(p-benzoic acid) (H_4mbtc), $Cu(NO_3)_2\cdot 2.5H_2O$ were obtained from the Shanghai Haohong Scientific Co.,Ltd. (Shanghai, China). Sodium hydroxide (NaOH), methanol (CH_3OH), ethanol (CH_3CH_2OH), N,N-dimethylformamide (DMF) and N,N-Diethylformamide (DEF) were purchased from Knowles Chengdu Chron Chemical Co., ltd. (Chengdu, China). All the water used was ultrapure water. All chemicals were analytical grade (AR) and used as received.

Instruments and characterizations

Gas sorption measurements

The N_2 (99.999%) adsorption isotherms of the materials was performed on ASAP 2460 at 77 K (Micromeritics, USA). Ar (99.999%) adsorption isotherms of the materials was performed on 3Flex at 87 K (Micromeritics, USA). Nonlocalized density functional theory (NLDFT) was used to obtain the pore size distribution of the materials, and Brunauer-Emmett-Teller (BET) model was used to describe the BET surface areas of the materials. The H_2 (99.999%), D_2 (99.999%) adsorption isotherms of the materials at 77 K and 87 K were measured on automatic volumetric adsorption apparatus (Micromeritics ASAP 2020 +).

In situ **Diffuse Reflectance Infrared Fourier-transformed (DRIFT)**

DRIFT spectra were collected in Nicolet IS50 spectrometer. Samples were loaded into a sample cup within a Harrick praying Mantis reaction chamber (CHC), two K-type thermocouples were placed in the bottom of the cup to monitor sample temperatures. The inlet/outlet of the chamber was connected to a homemade gas evacuation and charging apparatus, MOF powders were loaded into the sample cup, and its bottom was filled with short copper wires to accelerate heat conduction. Activated MOF was obtained by in situ heating MOF powders up to 120 °C under vacuum with a ramp of 4 °C /min and then maintaining at 120 °C for ~1.5 h. Hydrogen gas was employed with the purity of ≥ 99.999%. During DRIFTs measurements, all spectra were collected using the spectrum of outgassed MOF sample as the background.

Cryogenic thermal desorption spectroscopy

The Cryogenic thermal desorption spectroscopy (TDS) is a method of observing gases desorbed from the surface while elevating the surface temperature after exposing the sample to a target gas pressure at a preset temperature. The mass spectrometer installed at the outlet can analyze and

detect the species and quantity of desorbed gas molecules in real time, and the separation factor can be obtained based on the ratio of desorbed isotope gases. The TDS of H_2/D_2 gas mixture on MOF was carried out using the experimental procedure recommended by the Max Planck Institute. First, about ~ 5 mg samples were placed in the sample chamber and activated under vacuum at 110 ℃ for 12 h (final pressure < 10^{-4} Pa). Then the sample chamber was assembled into the ACTDS experimental apparatus for evacuation (final pressure < 10^{-3} Pa). At the same time, the sample chamber was cooled down and maintained for a period of time after the sample chamber was reduced to the experimental temperature. A H_2/D_2 mixture of 1.0 kPa was injected into the sample tube (nD_2:nH_2 = 1:1.1636) and maintained for 10 min, the H_2/D_2 mixture gas saturated competitive adsorption was completed. After the H_2/D_2 mixture was completely saturated and competitively adsorbed, the unattached H_2/D_2 mixture was rapidly evacuated within 10 minutes. The sample tube was then rapidly cooled to 20 K at a rate of 15 K/min. For desorption, the sample was heated with a rate of 0.1 K s^{-1} from 20 K to 150 K and the desorbing gas was continuously detected by a quadrupole mass spectrometer. (after absolute quantity calibration, the peak area of H_2/D_2 signal in MS response was proportional to the capacity of H_2/D_2), which corresponded to the desorption H_2/D_2 capacity, that is, the capacity of H_2/D_2 adsorbed by the material.

Dynamic column breakthrough experiment

The real-time low-temperature apparatus coupled with mass spectrometer (PFEIFFER VACUUM) was used for receiving the breakthrough curves. $H_2/D_2/Ne$ (10/10/80, vol.%) gas mixture at a flow rate of 5 mL/min and 77 K and 100 kPa conditions were used. The flow rate of the gas mixture was controlled by a mass flowmeter at the entrance, and the composition of the gas at the exit was monitored by mass spectrometer.

Isosteric Enthalpy of Adsorption

The isosteric enthalpies of adsorption for hydrogen and deuterium were calculated using the isotherms at 77 K and 87 K, following the Viral equation:

$$\ln(p) = \ln(N) + \frac{1}{T}\sum_{i=1}^{m} a_i \times N^i + \sum_{j=1}^{n} b_j \times N^j$$

Where P is the pressure (mmHg), N is the capacity adsorbed (mg g^{-1}), T is the temperature (K), a_i and b_j represents Viral coefficients, and m, n is the number of coefficients required to detailed describe the isotherms. The values of the Viria coefficients a_0 to am were then used to calculate the Isosteric enthalpy of adsorption according to the following equation:

$$Q_{st} = -R \times \sum_{i=0}^{m} a_i \times N^i$$

Q_{st} is the isosteric enthalpy of adsorption in kJ mol^{-1} and R is universal gas constant with the value of 8.314 J mol^{-1} K^{-1}.

Selectivity by IAST

Ideal adsorption solution theory (IAST) was calculated to evaluate the hydrogen isotope capture performance. [4] Double-site Langmuir (DSL) equation was firstly employed to fit the single-component gas equilibrium adsorption isotherms of MOF over the full pressure range. DSL equation can be written as:

$$N = A_1 \frac{b_1 p}{1 + b_1 p} + A_2 \frac{b_2 p}{1 + b_2 p}$$

Where N is the adsorption capacity (mmol g^{-1}), p is the pressure of bulk gas at equilibrium with adsorbed phase (bar), A_1 and A_2 are the saturation loadings for sites 1 and 2, respectively. b_1 and b_2 are the Langmuir parameters for sites 1 and 2. The parameters of A_1, A_2, b_1 and b_2 were then used to predict the adsorption selectivity based on IAST, which is finally defined as:

$$S = \frac{x_1/x_2}{y_1/y_2}$$

Where S represents the ideal selectivity of component 1 over component 2, x_i x_1 and y_i are the adsorbed phase mole fractions and the gas phase mole fractions, respectively.

Adsorption Capacity Calculation Based on Breakthrough Experiments

On the basis of mass balance, the gas adsorption amounts based on breakthrough experiments can be defined as follows [5] :

$$q_i = \frac{a_i V}{m \times V_m} \int_0^t \left(1 - \frac{y_i}{y_0}\right) dt$$

Where qi is the equilibrium adsorption amount of component i (mmol g^{-1}), ai is the percent of component i in the mixture, V is the flow rate of the mixed gas (mL min^{-1}), m is the quality of activated sample packed in the sample column (g), t is the adsorption time for the component i (min), y_0 and y_i are the inlet and outlet gas molar flow rates, respectively. V_m = 22.4 L/mol. L mol^{-1}. The separation factor (α) of the breakthrough test is defined as follows:

$$\alpha = \frac{q_i/q_j}{y_i/y_j}$$

Where y_i and y_j represent the molar fractions of component i and j in the steel bottle, respectively.

Computational method

The crystal structures used for the parameterizations and simulations in Cu-ATC, Cu-bptc and Cu-mbtc herein were taken from ref. 23, 25 and 34, respectively (refcodes BIMDIL, SUKYIH and LASYOU from the Cambridge Structure Database (CSD)). The adsorption simulation was performed using the Materials Studio. All giant canonical Monte Carlo (GCMC) calculations in this work were accomplished by Sorption package in Materials Studio. The Universal Force field was used to describe MOF parameters. The MOFs symmetry was changed to P1. To get stable geometry, optimizations were obtained using a Forcite module, GCMC calculations were performed by Sorption module where Universal forcefield and Metropolis method were used. L-J 12-6 potential was used to describe the van der Waals interactions between MOF and adsorbate materials, while Ewald summation method described the electrostatic interactions. The equilibrium steps were set as 1×10^6 and 1×10^7 production steps for sampling thermodynamic properties.

Following the determination of H_2 adsorption sites within these MOFs, we conducted density functional theory (DFT) calculations to quantify adsorption energies at each identified site. All computations All calculations in this work were accomplished by Dmol3 package in Materials Studio with DNP basis set. As for the exchange-correlation functional, the GGA-PW91 method was chosen, which has been confirmed appropriate for our system in previous works. [6-8] The space

cut-off radius was set as 4.5 Å and OBS method was adopted for DFT-D correction. The geometry optimization was performed until the energy, force and displacement on each atom was converged to less than 2.0×10^{-5} Ha, 4.0×10^{-3} Ha/Å, 5.0×10^{-3} Å, respectively. The molecule adsorption energy (Eads) was defined according to the following equation:

$$E_{ads} = E_{MOF/H2} - E_{MOF} - E_{H2}$$

where $E_{MOF/H2}$ represents the total energy when "H_2" molecule adsorbed on MOFs, E_{MOF} means the energy of MOFs with no "H_2" molecule adsorption, while E_{H2} is the energy of dissociative "H_2" molecules.

In VASP, [9-12] the electronic exchange-correlation has been treated using the Perdew–Burke–Ernzerhof (PBE) functional within the generalized gradient approximation (GGA). [13] The interaction between atomic nuclei and valence electrons has been modeled using projector augmented wave (PAW) pseudopotentials, with a plane-wave cutoff energy set to 500 eV. [14] The convergence criterion for self-consistent iterations is set to 1.0×10^{-5} eV, and structural optimization is considered complete when forces on all atoms are less than 0.02 eV/Å^{-1}. A 3 × 3 × 2 k-point mesh is used for all calculations. The computational models used in this study have been constructed based on the Cu-ATC model and the D_2 molecule is put between two Cu atoms of Cu-ATC, and the lattice parameters of the constructed models are a = b = 8.467 Å, c = 14.444 Å while $\alpha = \beta = \gamma = 90°$ (see Figure 13a). The adsorption energies are calculated using the following equation:

$$Eads = -(E_{total} - E_{Cu} - E_{D2})$$

Here, E_{total}, E_{Cu}, and E_{D2} represent energies of D_2 adsorbed in the Cu-ATC model, the Cu-ATC model itself, and D_2 molecules, respectively. The charge density difference (CDD) is calculated using VASPKIT (see Figure 13b). [15]

The adsorption energy for D_2 molecule is calculated to be 0.1636 eV and the CDD indicates the interaction between the D_2 molecules and the Cu atoms.

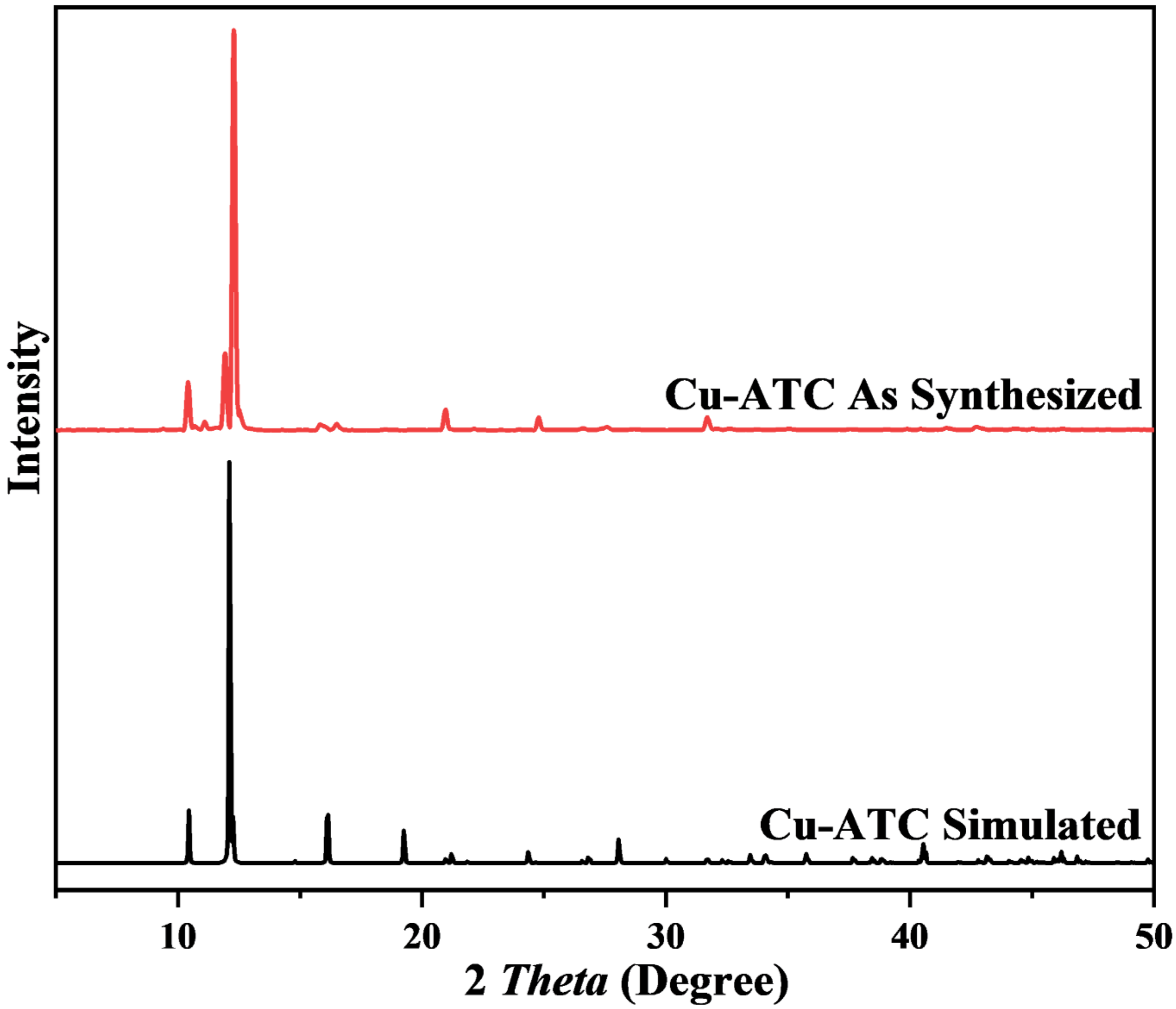

Figure S1. The calculated and synthesized PXRD patterns of Cu-ATC.

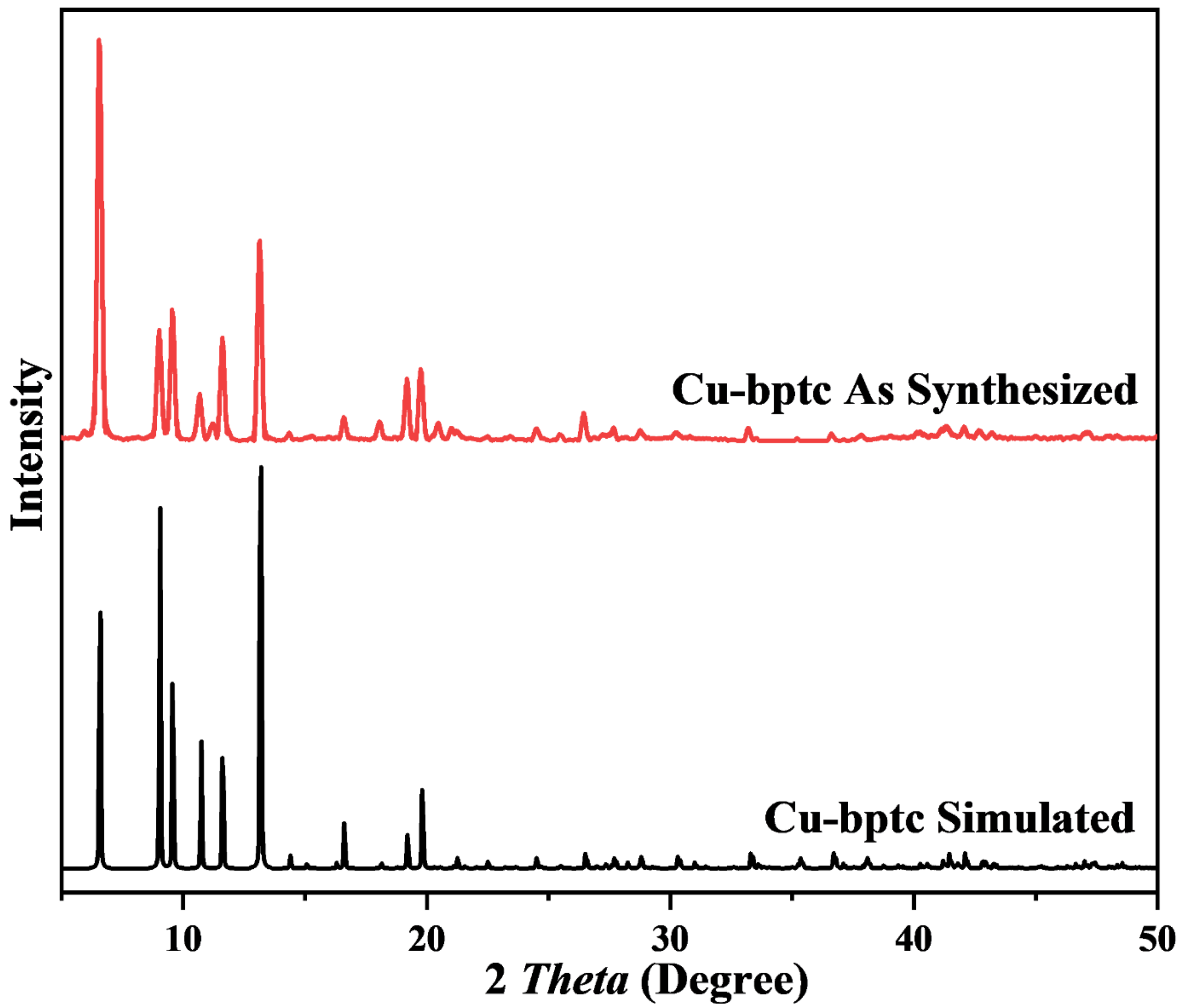

Figure S2. The calculated and synthesized PXRD patterns of Cu-bptc.

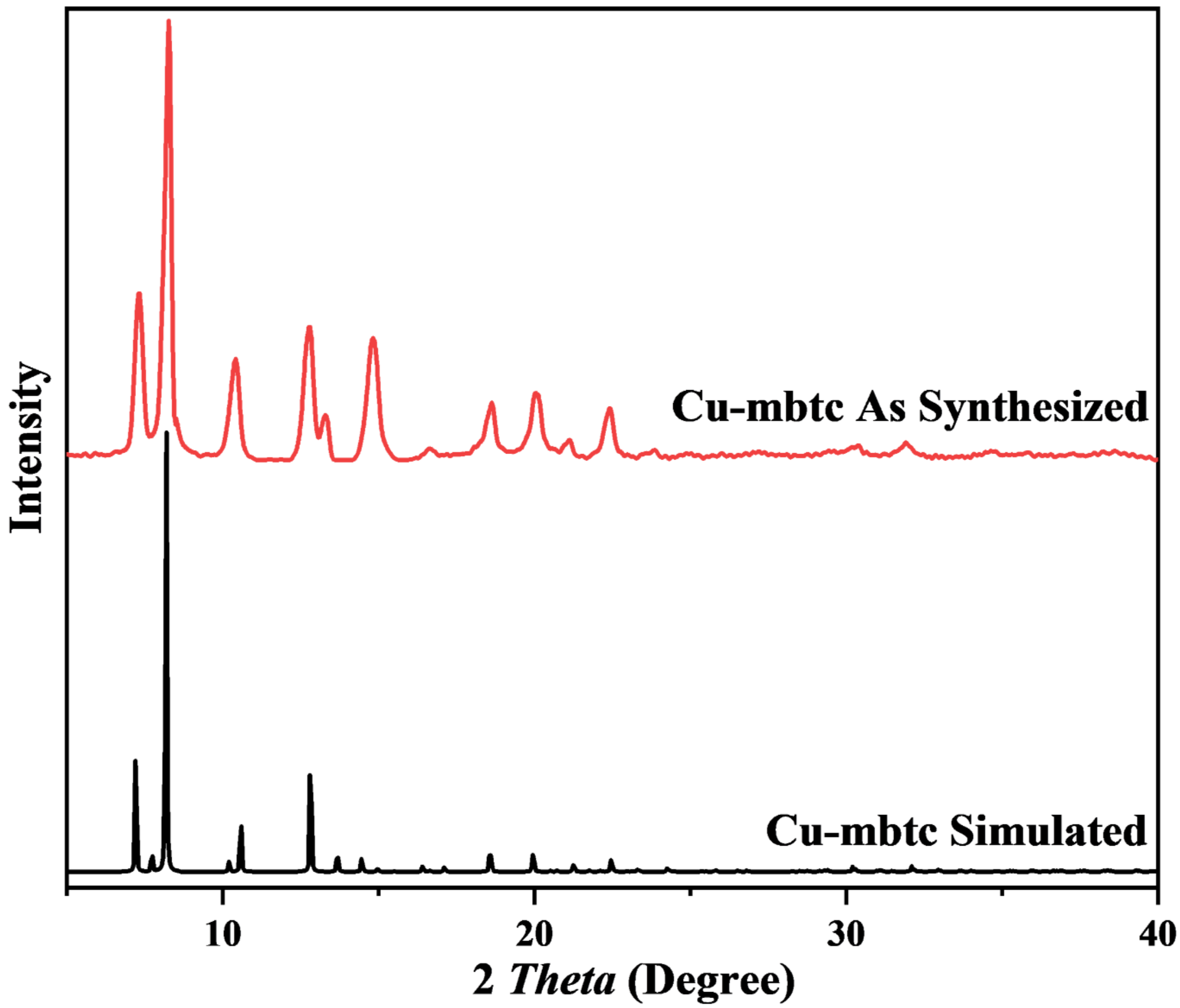

Figure S3. The calculated and synthesized PXRD patterns of Cu-mbtc.

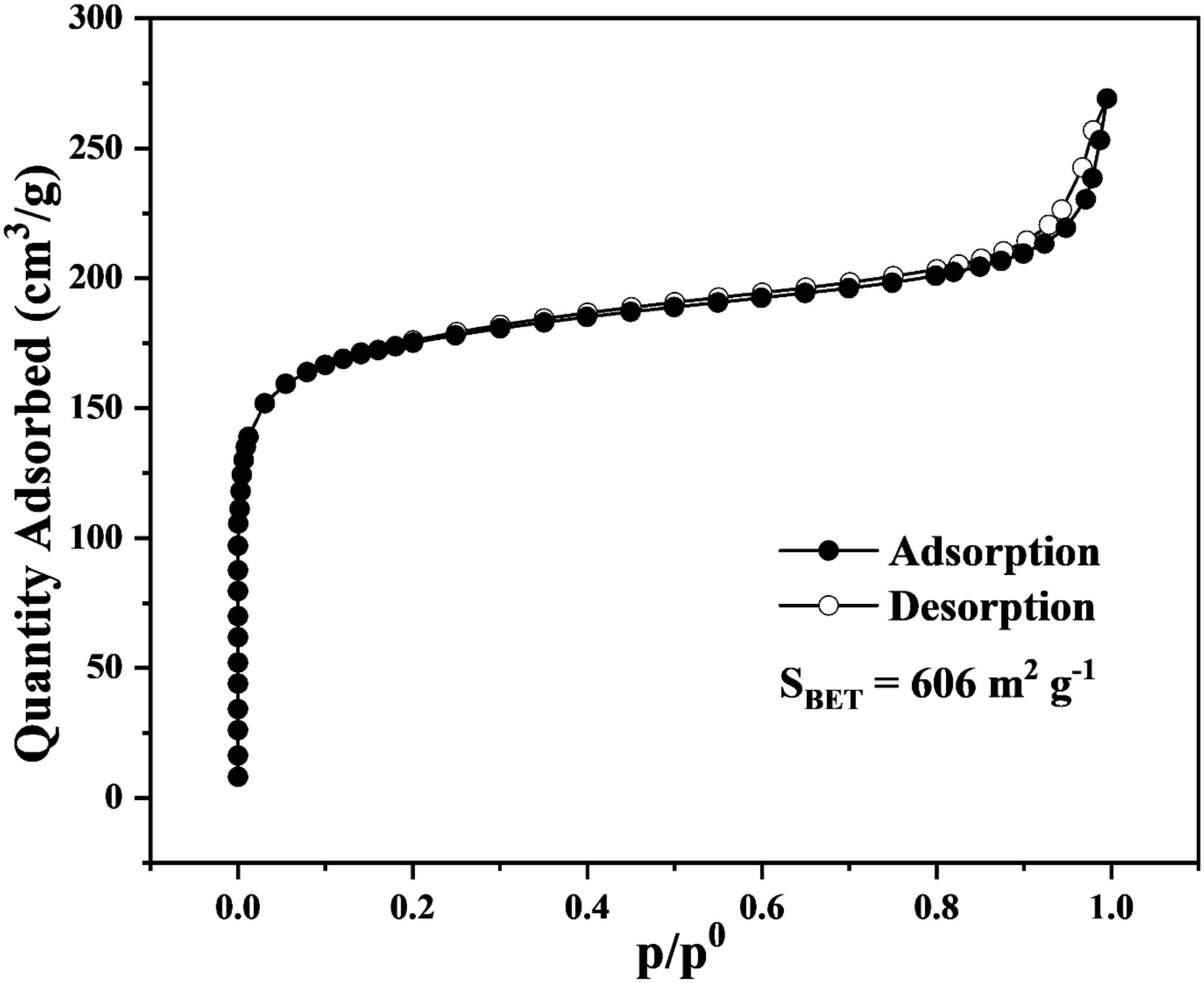

Figure S4. N_2 isotherm for Cu-ATC at 77 K.

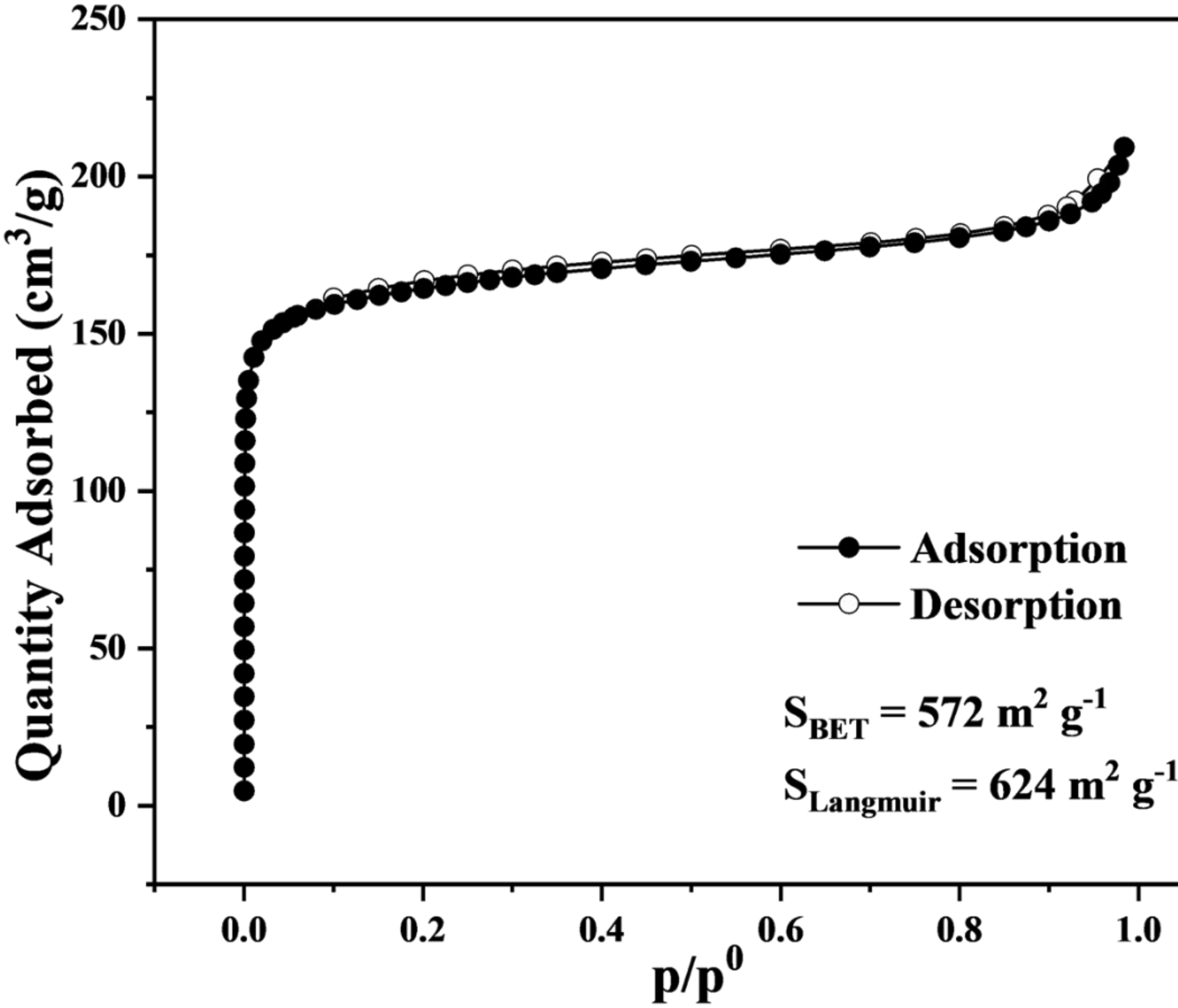

Figure S5. Ar isotherm for Cu-ATC at 87 K.

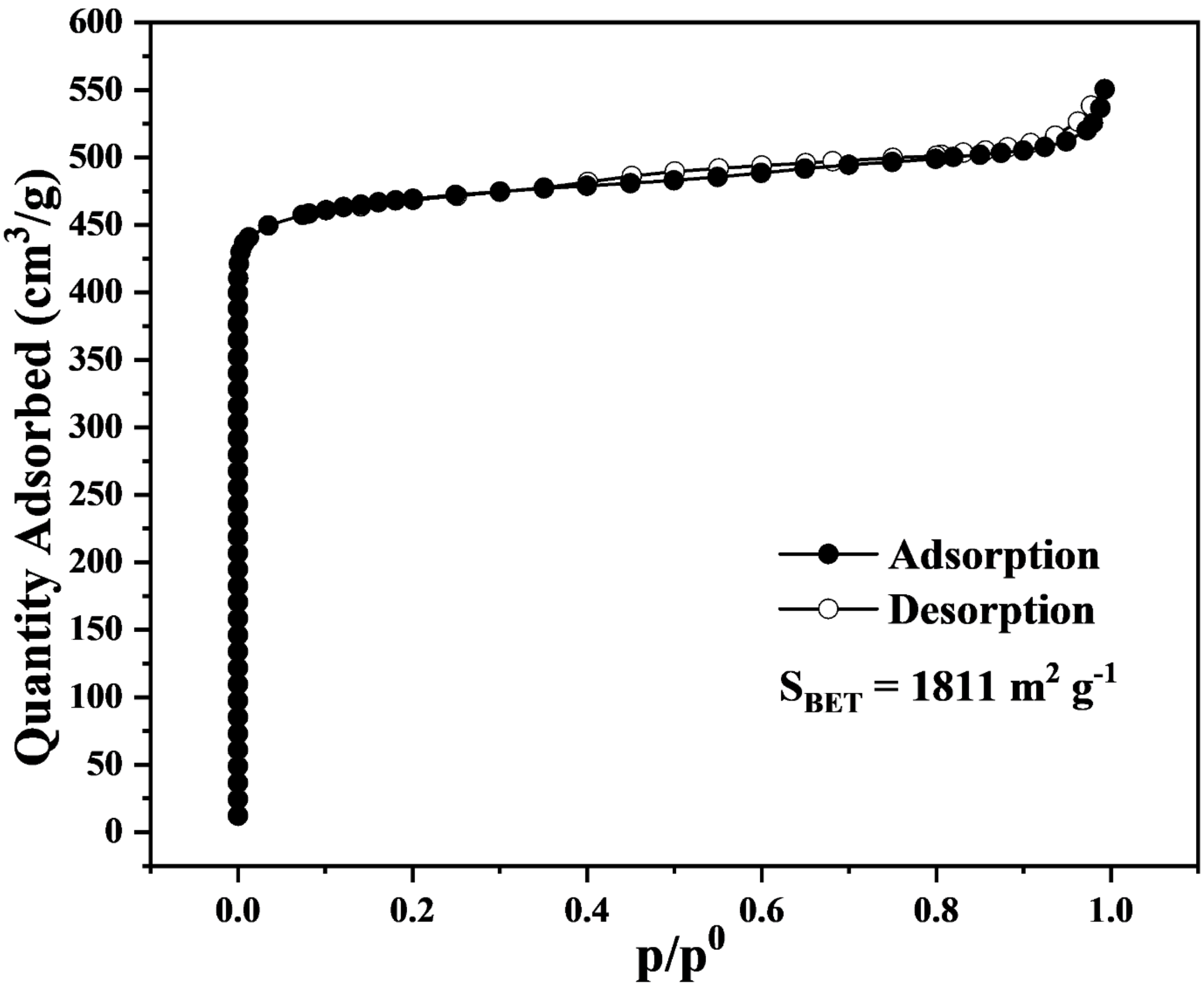

Figure S6. N_2 isotherm for Cu-bptc at 77 K.

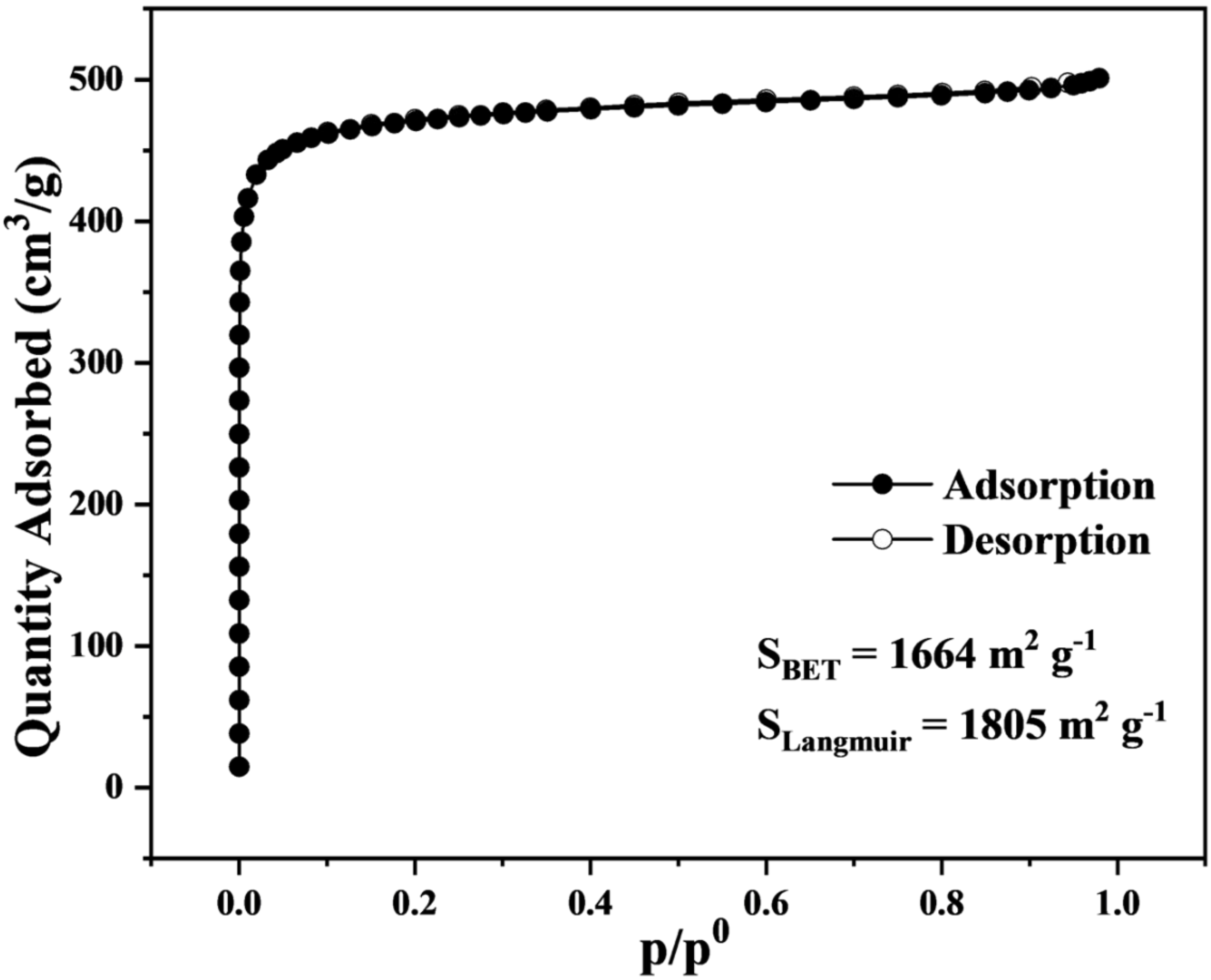

Figure S7. Ar isotherm for Cu-bptc at 87 K.

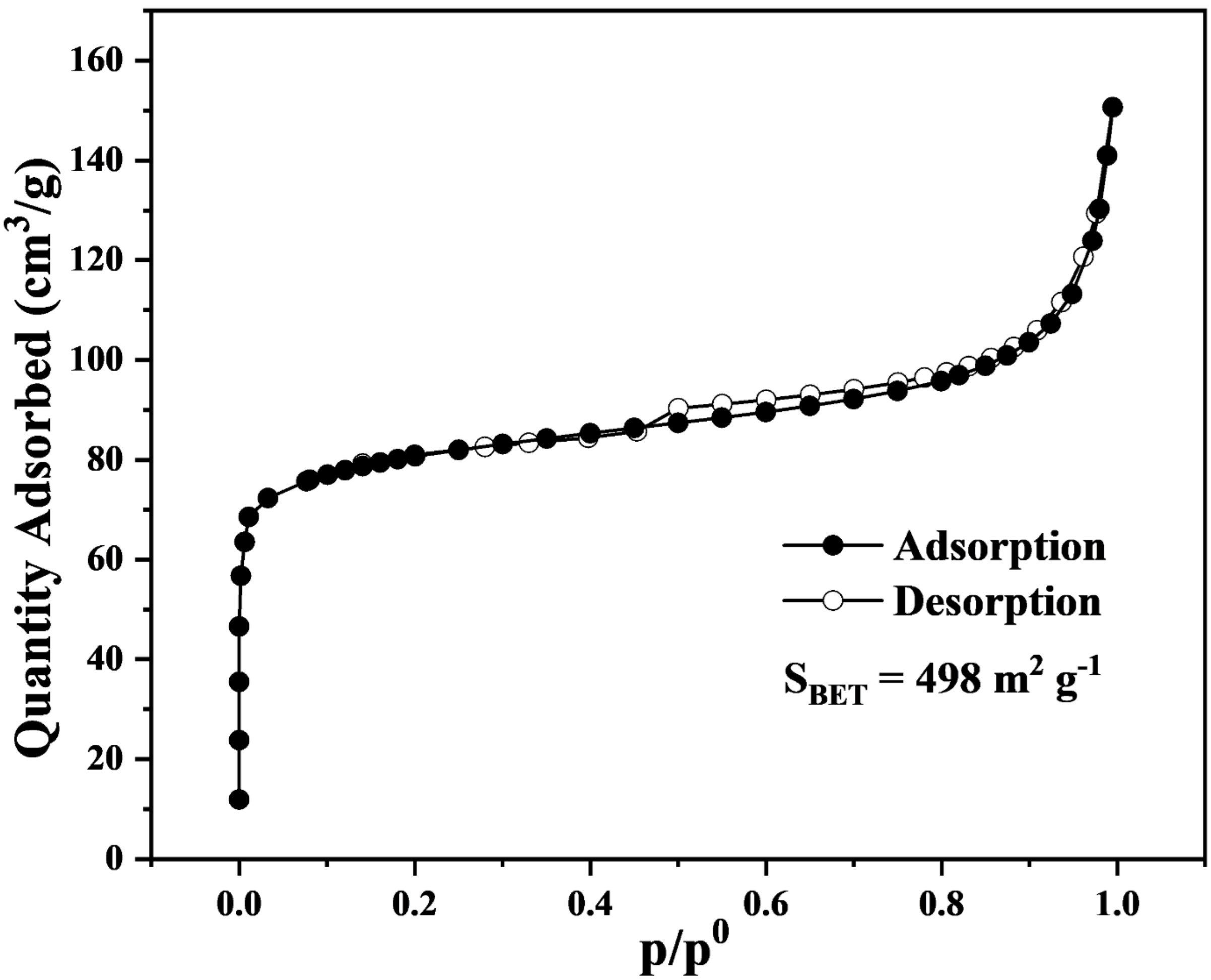

Figure S8. N_2 isotherm for Cu-mbtc at 77 K.

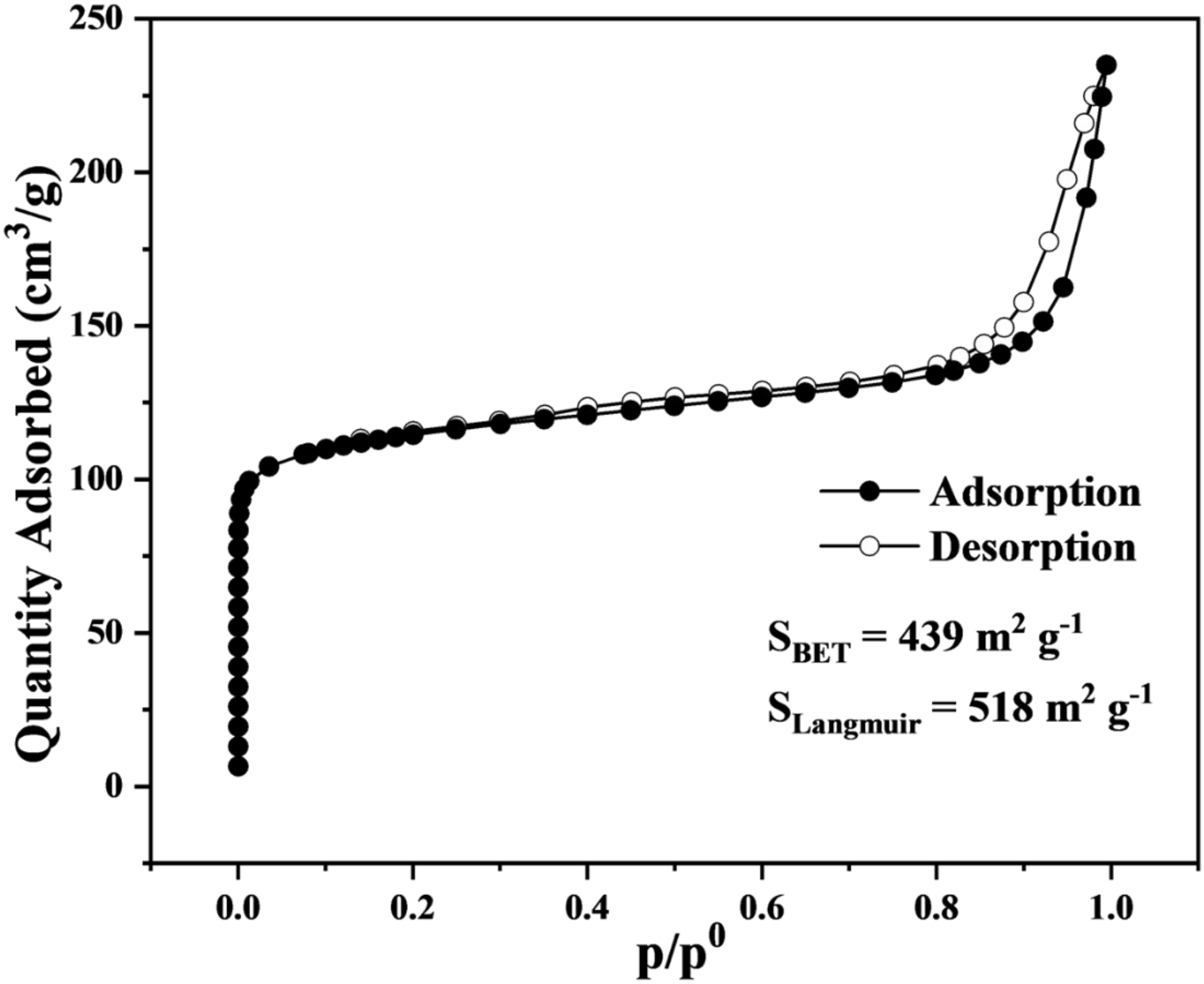

Figure S9. Ar isotherm for Cu-mbtc at 87 K.

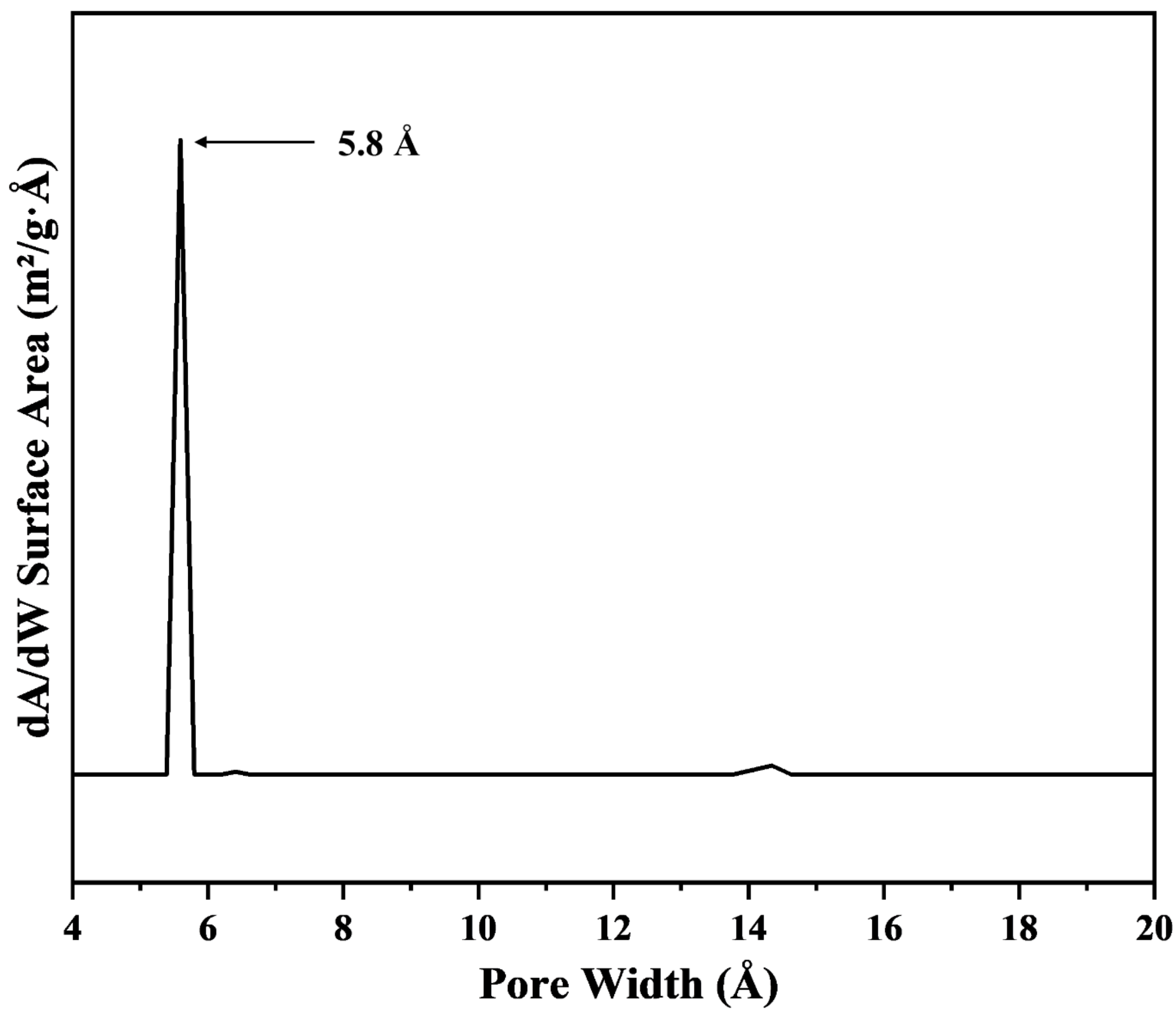

Figure S10. Pore size distributions from NLDFT method of Cu-ATC.

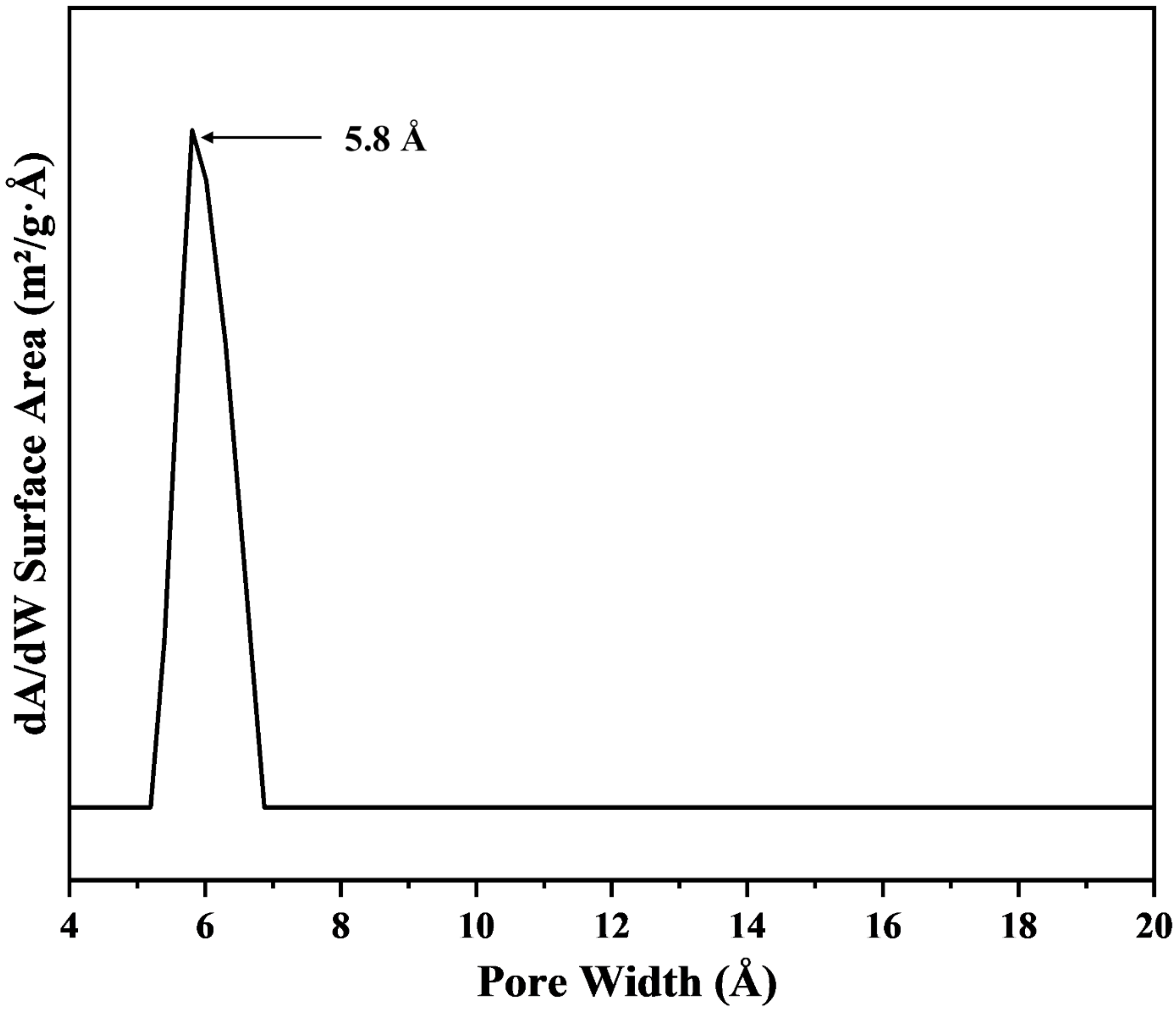

Figure S11. Pore size distributions from NLDFT method of Cu-bptc.

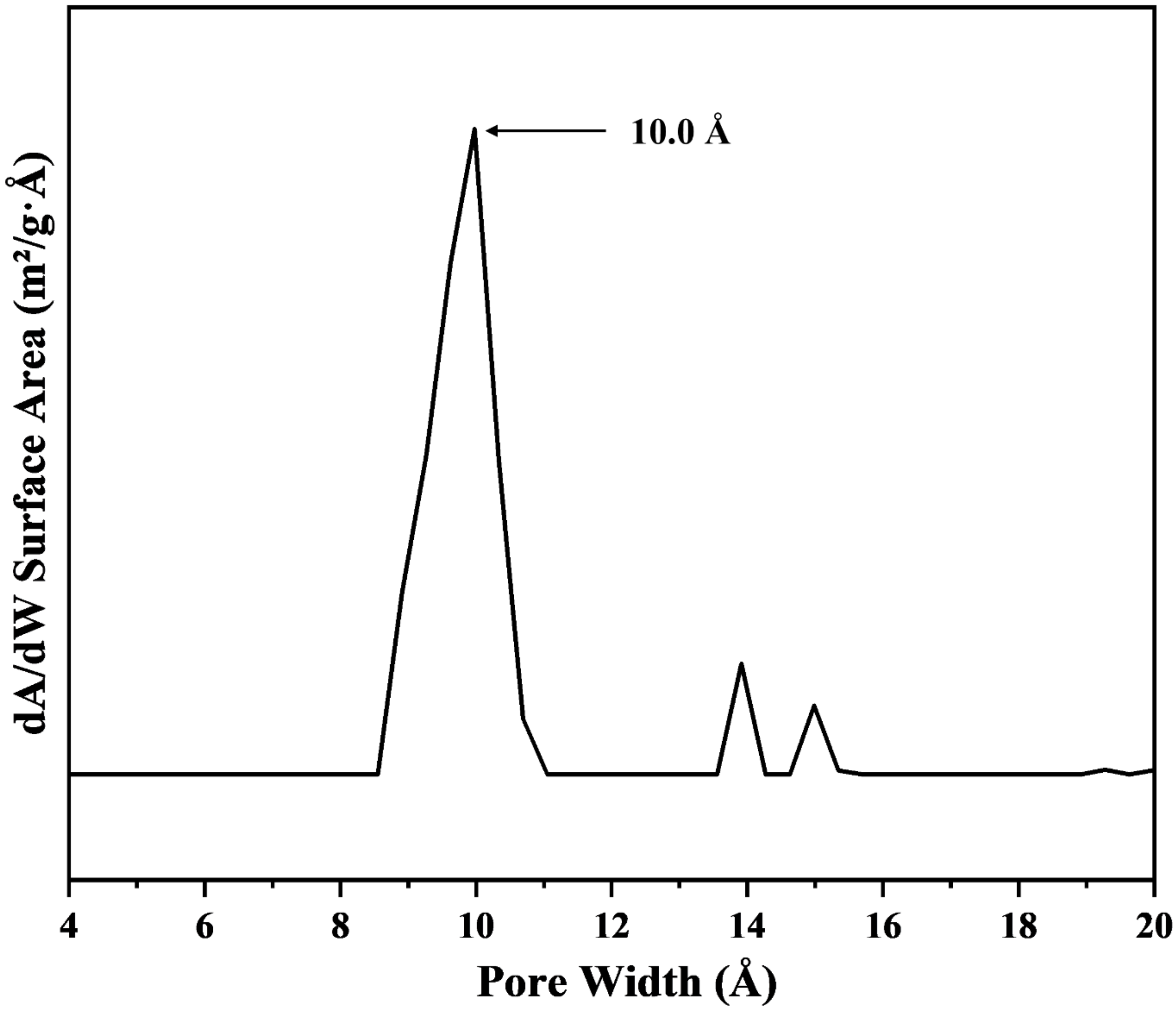

Figure S12. Pore size distributions from NLDFT method of Cu-mbtc.

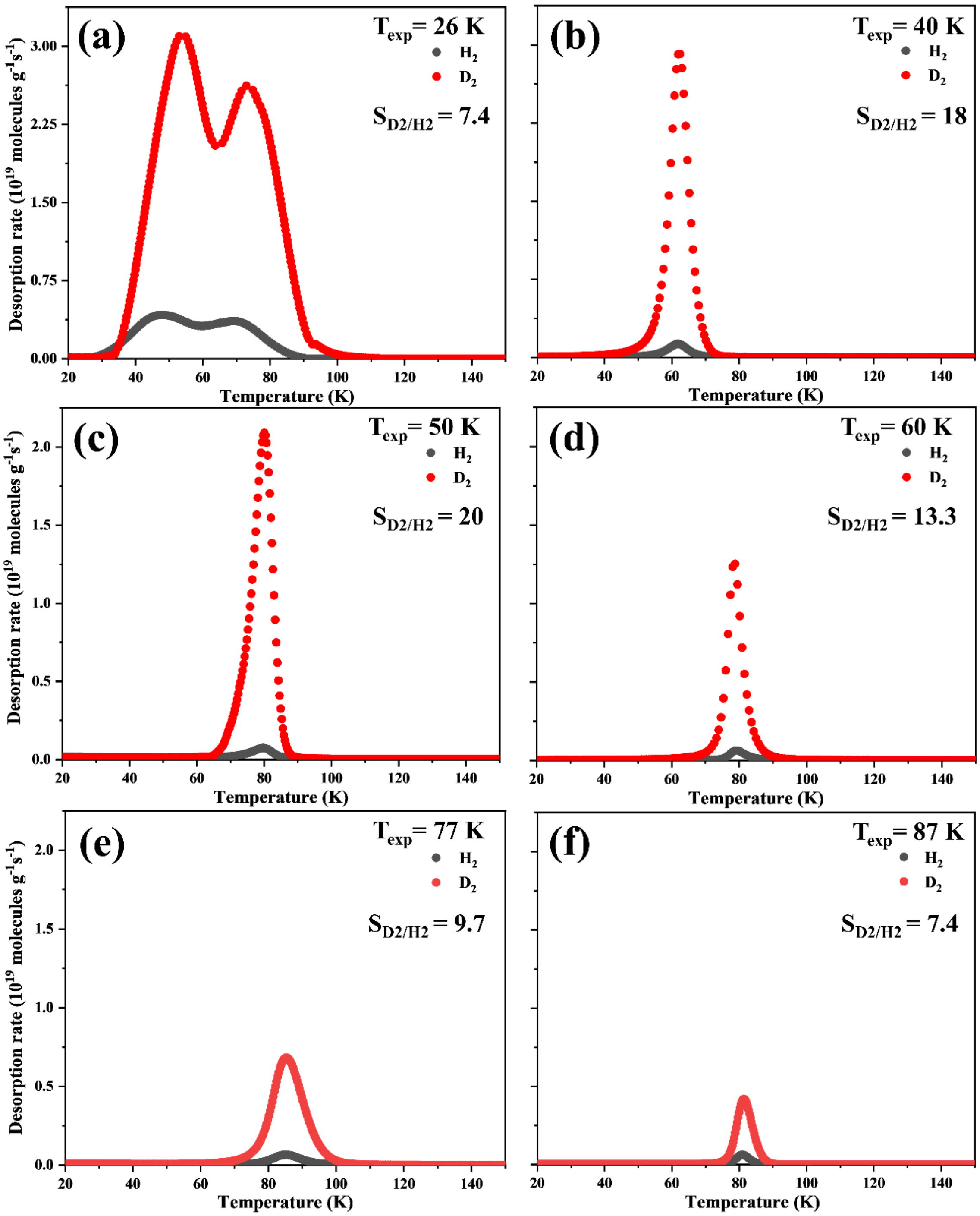

Figure S13. H_2 (black) and D_2 (red) desorption spectra of 10 mbar (1:1 D_2/H_2 mixture) loading on Cu-ATC with a heating rate of 0.1 K/s. Exposure temperature (T_{exp}) at (a) 26 K, (b) 40 K, (c) 50 K, (d) 60 K, (e) 77 K and (f) 87 K.

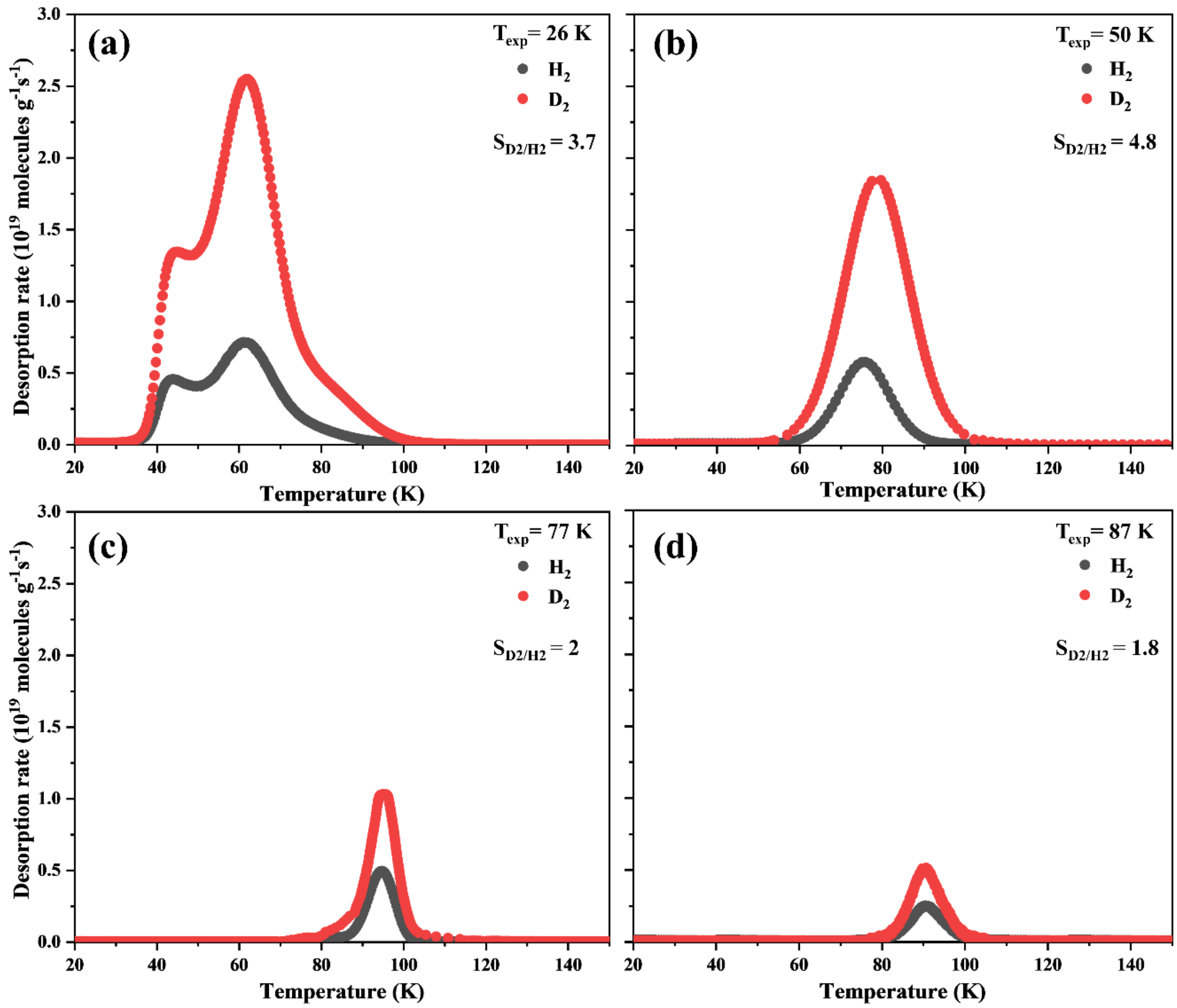

Figure S14. H_2 (black) and D_2 (red) desorption spectra of 10 mbar (1:1 D_2/H_2 mixture) loading on Cu-bptc with a heating rate of 0.1 K/s. Exposure temperature (T_{exp}) at (a) 26 K, (b) 50 K, (c) 77 K and (d) 87 K.

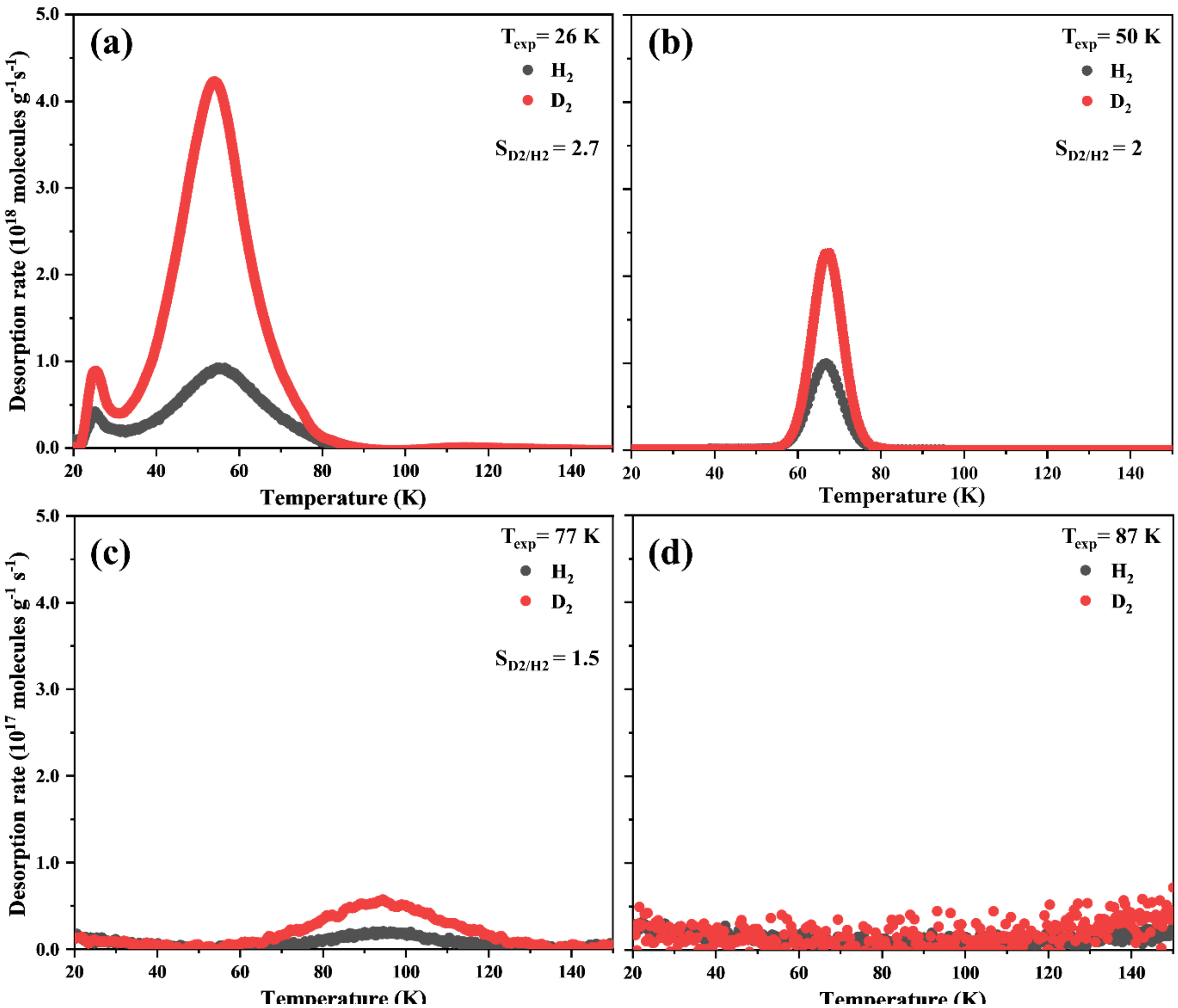

Figure S15. H_2 (black) and D_2 (red) desorption spectra of 10 mbar (1:1 D_2/H_2 mixture) loading on Cu-mbtc with a heating rate of 0.1 K/s. Exposure temperature (T_{exp}) at (a) 26 K, (b) 50 K, (c) 77 K and (d) 87 K.

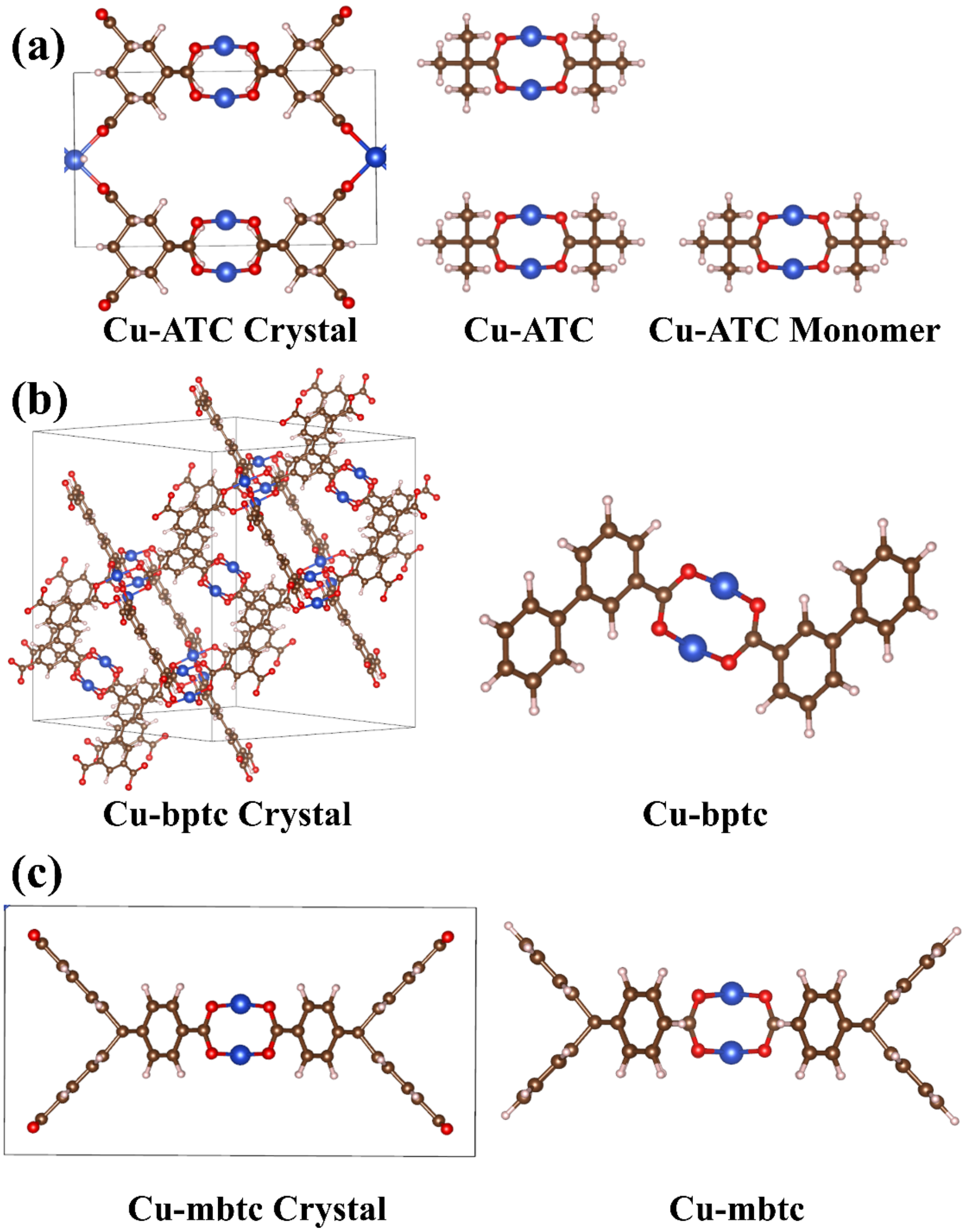

Figure S16. (a) Cu-ATC, (b) Cu-bptc, and (c) Cu-mbtc: Structural models used for DFT calculations, illustrating both the full crystal structure and extracted molecular fragments. (blue, copper; red, oxygen; tan, carbon; pink, hydrogen)

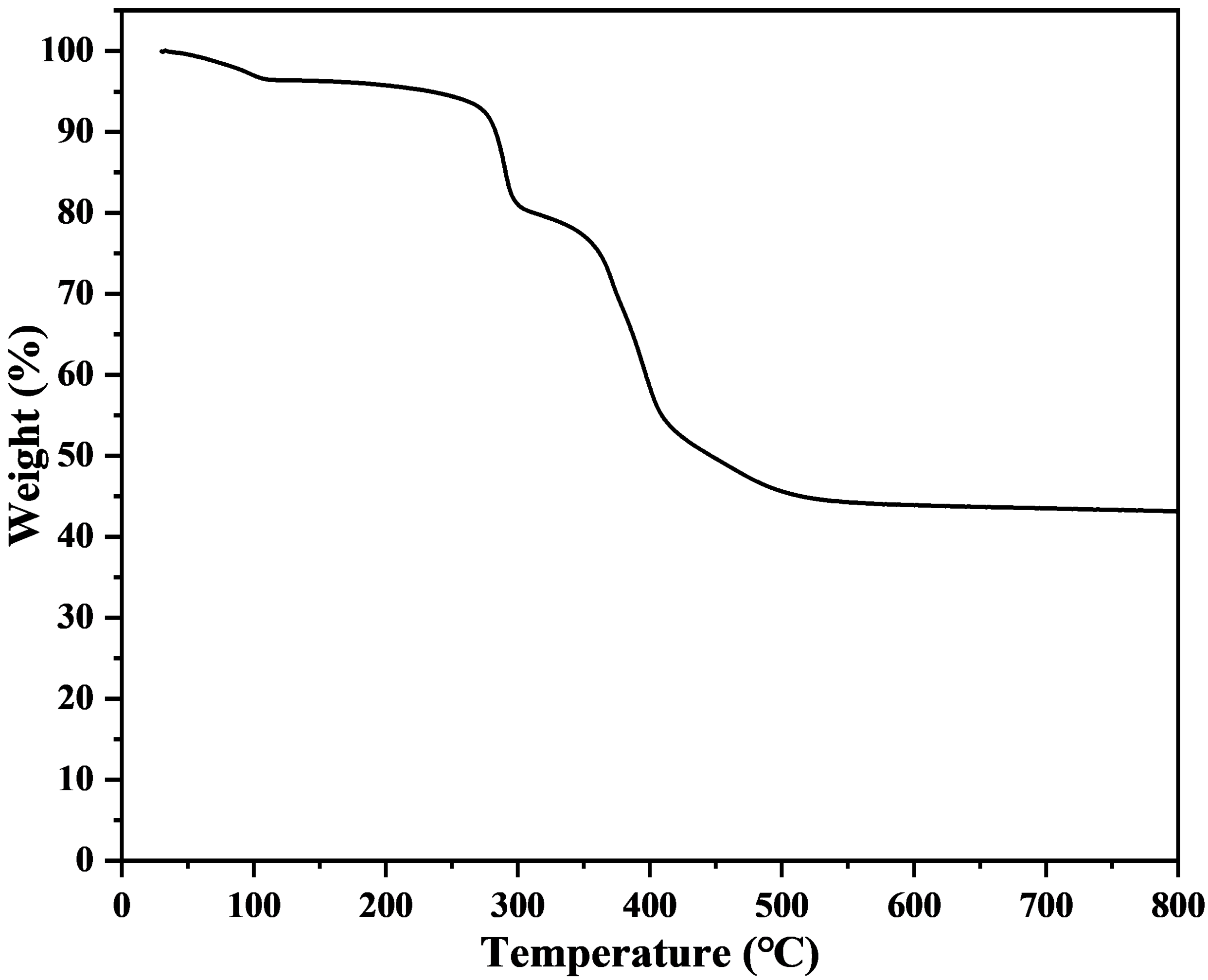

Figure S17. The TGA curve of Cu-ATC from room temperature to 800 °C.

Compound	method	T_{exp}(K)	P(mbar)	t_{exp}(min)	$S_{D2/H2}$(1:1 mixture)	Ref.
Cu-ATC	TDS	**26/40/50/60/77/87**	10	**10**	**7.4/18/20/13.3/9.7/7.4**	This work
Cu-bptc	TDS	**26/50/77/87**	10	**10**	**3.7/4.8/2/1.8**	This work
Cu-mbtc	TDS	**26/50/77**	10	**10**	**2.7/2/1.5**	This work
MOF-74-ac	TDS	20/40/**60**/**77**	10	**10**	7.1/11.4/**16.1**/**19.1**	16
MOF-74-IM-10	TDS	20/40/**60**/**77**	10	**10**	5.7/9.2/**19.6**/**25.8**	16
MOF-74-IM-38	TDS	20/40/**60**/**77**	10	**10**	8.5/12.2/**11.3**/**5.2**	16
Cu(I)-MFU-4l	TDS	90/100	10	**10**	7.1/11.1	17
CPO-27-Co	TDS	20/30/**40**/**50**/**60**/**77**	10	**10**	1.9/2.2/2.6/**2.9**/**5.8**/**5.5**/**5.4**	18
Ni_2Cl_2BBTA	TDS	**77**/**87**	10	**10**	**4.5**/**4**	19
Cu(I)Cu(II)-BTC	TDS	25/30/40/**50**/**60**	10	**10**	34.4/37.9/7.4/**2.9**/**1**	20
Cu(II)-BTC	TDS	25/30/**40**/**50**/**60**	10	**10**	4.3/**5.2**/**1.5**/**3**	20
Ag(I)-ZSM-5	TDS	40/**60**/70	10	**10**	2.4/**3.5**/9.1	21
Cu(I)-ZSM-5	TDS	**60**/100	10	**10**	**2.8**/24.9	22

Table S1. Summary of hydrogen isotope separation factors for various MOFs as a function of working temperature

Structures	H_2	H_2 red shift	D_2	D_2 red shift
Cu-ATC	3931 (3958) cm^{-1}	230 (203) cm^{-1}	2830 (2846) cm^{-1}	163 (147) cm^{-1}
Cu-bptc	4011 (4019) cm^{-1}	150 (142) cm^{-1}	2886(2888) cm^{-1}	107 (105) cm^{-1}
Cu-ATC monomer	4011 cm^{-1}	152 cm^{-1}	2884 cm^{-1}	109 cm^{-1}

Table S2. The DFT-computed vibrational frequencies of H_2 and D_2 adsorbed on Cu-ATC, Cu-bptc, and the Cu-ATC monomer, with experimental values given in parentheses.

Supplementary References:

1 Chen, B. *et al.* Cu2(ATC)·6H2O: Design of Open Metal Sites in Porous Metal−Organic Crystals (ATC: 1,3,5,7-Adamantane Tetracarboxylate). *Journal of the American Chemical Society* **122**, 11559-11560, doi:10.1021/ja003159k (2000).

2 Chen, B., Ockwig, N. W., Millward, A. R., Contreras, D. S. & Yaghi, O. M. High H2 Adsorption in a Microporous Metal–Organic Framework with Open Metal Sites. *Angewandte Chemie International Edition* **44**, 4745-4749, doi:https://doi.org/10.1002/anie.200462787 (2005).

3 Ma, L., Jin, A., Xie, Z. & Lin, W. Freeze Drying Significantly Increases Permanent Porosity and Hydrogen Uptake in 4,4-Connected Metal–Organic Frameworks. *Angewandte Chemie International Edition* **48**, 9905-9908, doi:https://doi.org/10.1002/anie.200904983 (2009).

4 Myers, A. L. & Prausnitz, J. M. Thermodynamics of mixed-gas adsorption. *AIChE Journal* **11**, 121-127, doi:https://doi.org/10.1002/aic.690110125 (1965).

5 Lin, R.-B. *et al.* Molecular sieving of ethylene from ethane using a rigid metal–organic framework. *Nature Materials* **17**, 1128-1133, doi:10.1038/s41563-018-0206-2 (2018).

6 Wu, Y. *et al.* Enhancement effect of lithium-doping functionalization on methanol adsorption in copper-based metal-organic framework. *Chemical Engineering Science* **123**, 1-10, doi:https://doi.org/10.1016/j.ces.2014.10.010 (2015).

7 Zhang, Q., Cao, L., Li, B. & Chen, L. Catalyzed activation of CO2 by a Lewis-base site in W–Cu–BTC hybrid metal organic frameworks. *Chemical Science* **3**, 2708-2715, doi:10.1039/C2SC20521A (2012).

8 Zhou, C., Cao, L., Wei, S., Zhang, Q. & Chen, L. A first principles study of gas adsorption on charged CuBTC. *Computational and Theoretical Chemistry* **976**, 153-160, doi:https://doi.org/10.1016/j.comptc.2011.08.018 (2011).

9 Kresse, G. & Furthmüller, J. Efficient iterative schemes for ab initio total-energy calculations using a plane-wave basis set. *Physical Review B* **54**, 11169-11186, doi:10.1103/PhysRevB.54.11169 (1996).

10 Kresse, G. & Hafner, J. Ab initio molecular dynamics for open-shell transition metals. *Physical Review B* **48**, 13115-13118, doi:10.1103/PhysRevB.48.13115 (1993).

11 Kresse, G. & Hafner, J. Ab initio molecular dynamics for liquid metals. *Physical Review B* **47**, 558-561, doi:10.1103/PhysRevB.47.558 (1993).

12 Kresse, G. & Hafner, J. Ab initio molecular-dynamics simulation of the liquid-metal--amorphous-semiconductor transition in germanium. *Physical Review B* **49**, 14251-14269, doi:10.1103/PhysRevB.49.14251 (1994).

13 Perdew, J. P., Burke, K. & Ernzerhof, M. Generalized Gradient Approximation Made Simple. *Physical Review Letters* **77**, 3865-3868, doi:10.1103/PhysRevLett.77.3865 (1996).

14 Blöchl, P. E. Projector augmented-wave method. *Physical Review B* **50**, 17953-17979, doi:10.1103/PhysRevB.50.17953 (1994).

15 Wang, V., Xu, N., Liu, J.-C., Tang, G. & Geng, W.-T. VASPKIT: A user-friendly interface facilitating high-throughput computing and analysis using VASP code. *Computer Physics Communications* **267**, 108033, doi:https://doi.org/10.1016/j.cpc.2021.108033 (2021).

16 Kim, J. Y. *et al.* Exploiting Diffusion Barrier and Chemical Affinity of Metal–Organic Frameworks for Efficient Hydrogen Isotope Separation. *Journal of the American Chemical Society* **139**, 15135-15141, doi:10.1021/jacs.7b07925 (2017).
17 Weinrauch, I. *et al.* Capture of heavy hydrogen isotopes in a metal-organic framework with active Cu(I) sites. *Nature Communications* **8**, 14496, doi:10.1038/ncomms14496 (2017).
18 Oh, H., Savchenko, I., Mavrandonakis, A., Heine, T. & Hirscher, M. Highly Effective Hydrogen Isotope Separation in Nanoporous Metal–Organic Frameworks with Open Metal Sites: Direct Measurement and Theoretical Analysis. *ACS Nano* **8**, 761-770, doi:10.1021/nn405420t (2014).
19 Li, X. *et al.* Highly selective adsorption of D2 from hydrogen isotopes mixture in a robust metal bistriazolate framework with open metal sites. *International Journal of Hydrogen Energy* **45**, 21547-21554, doi:https://doi.org/10.1016/j.ijhydene.2020.05.096 (2020).
20 Hu, X. *et al.* Highly Effective H2/D2 Separation within the Stable Cu(I)Cu(II)-BTC: The Effect of Cu(I) Structure on Quantum Sieving. *ACS Applied Materials & Interfaces* **15**, 3941-3952, doi:10.1021/acsami.2c18221 (2023).
21 Xiong, R. *et al.* Hydrogen isotopes separation in Ag(I) exchanged ZSM-5 zeolite through strong chemical affinity quantum sieving. *Microporous and Mesoporous Materials* **313**, 110820, doi:https://doi.org/10.1016/j.micromeso.2020.110820 (2021).
22 Xiong, R. *et al.* Highly effective hydrogen isotope separation through dihydrogen bond on Cu(I)-exchanged zeolites well above liquid nitrogen temperature. *Chemical Engineering Journal* **391**, 123485, doi:https://doi.org/10.1016/j.cej.2019.123485 (2020).
