# Supplementary material for: Synergistic effect of paired Cu(II) open metal sites for enhanced high-temperature hydrogen isotope separation
Source: Nat Commun. 2026 Aug 10;17:9585. doi: 10.1038/s41467-026-76556-7 (PMC13550398; doi:10.1038/s41467-026-76556-7)
Supplement: Supplementary file 2 [file 41467_2026_76556_MOESM2_ESM.pdf]

nature portfolio

Peer Review File

Synergistic Effect of Paired Cu(II) Open Metal Sites for Enhanced High-Temperature Hydrogen Isotope Separation

Corresponding Author: Professor Chuanqin Xia

This file contains all reviewer reports in order by version, followed by all author rebuttals in order by version.

Version 0:

Reviewer comments:

Reviewer #1

(Remarks to the Author)
The manuscript claims that deuterium/hydrogen isotope separation is amplified by a dual-Cu open-metal-site (OMS) arrangement in an ultramicroporous Cu-MOF. However, the experimental evidence provided does not directly demonstrate that two adjacent OMSs cooperatively bind a single H_2/D_2 molecule, and several datasets that should track smoothly with this hypothesis show non-monotonic or even contradictory behavior. Because the central mechanistic claim is insufficiently supported and several key figures contain internal inconsistencies, I cannot recommend publication in the present form.

Major comments
1. The main point of this paper is that "dual (paired) Cu OMSs" improve the adsorption strength and thus enhance D_2/H_2 selectivity. However, the manuscript does not provide any experiments that directly demonstrate two cooperative Cu sites binding the same molecule. The only site-discriminating measurement is the low-temperature temperature-programmed desorption (TDS), which shows multiple desorption features. This only indicates that "more than one type of site exists (e.g. weak and strong site)," rather than proving that "two Cu sites act synergistically on one adsorbate." All other evidence presented (such as the higher heat of adsorption (Qst) for Cu-ATC, the red shift observed in DRIFTS at 298 K, GCMC/DFT maps, and breakthrough selectivity) is indirect and could also support a simpler explanation: that "Cu-ATC contains one relatively strong OMS located in a narrow one-dimensional channel."
Similarly, the unusually large red shift (≈682 cm^{-1}) observed only for Cu-ATC is used as a key argument for “strong, cooperative” binding. However, (i) the shift is very large for RT H_2 on Cu(II) OMS; (ii) there is no D_2 analogue to demonstrate isotope-sensitive binding at the same site; and (iii) Cu-bptc and Cu-mbtc show essentially no signal under otherwise identical conditions. In its current form the DRIFTS result shows “Cu-ATC has a strong site,” not “Cu-ATC has two Cu sites acting together.”
2. The highest reported separation factor occurs at 50 K. In a typical "two-site" mechanism, where the population of strong and weak sites gradually changes with temperature, we would expect a more consistent or smoothly varying selectivity. However, the data reveal a significant peak specifically at 50 K.
3. In Fig. 2(a–c), the H_2 and D_2 uptakes of the three materials are very close at 77/87 K (differences are small). However, in Fig. 2(d) the loading trace/line for D_2 is visibly much longer (roughly twice) than that for H_2, for all three materials. This is not consistent with the preceding panels.
4. The TDS data appear to suffer from a procedural issue. In the SI, the exposure temperature and the TDS onset/peak temperatures do not line up consistently, and this mismatch is especially visible for the higher-temperature exposures. This is a well-known artefact in low-temperature isotope TDS: if, after high-T exposure, the sample is pumped under vacuum for too long at (or near) the exposure temperature, a non-negligible fraction of the more weakly bound hydrogen will already desorb during the pumping step. What remains on the sample when the actual TDS ramp starts is then an enriched, slower-desorbing fraction, so the apparent D_2/H_2 selectivity looks better than it really is.
- Temperature-dependent pumping time must be defined and justified.
- Show reproducibility
- Align exposure T and TDS onset.
5. The manuscript reports IAST-based D_2/H_2 selectivities (e.g. Cu-ATC: 1.84 at 77 K; 1.60 at 87 K) only in the main text, but provides no description of the actual IAST procedure. Specifically, it is not stated which isotherm model(s) were fitted to the single-component H_2 and D_2 data (Langmuir, dual-site Langmuir, Langmuir–Freundlich, or another form), the fitted parameters, goodness of fit, and the coverage/pressure ranges used for the fits are not given, the composition and total pressure used for the IAST prediction (nominally “equimolar”) are not cross-checked against the experimental mixture used

in TDS and breakthrough, no IAST curves (selectivity vs pressure, or vs loading) are shown for the three MOFs.

Minor comments

a. The manuscript repeatedly uses the term “bimetallic synergistic engineering” although all presented frameworks are monometallic Cu(II) paddlewheels. Please change to “paired Cu(II) OMS” or “dual-site Cu(II).”

b. The comparison plot with literature mixes data taken at different pressures (10 mbar vs 60 mbar) and slightly different temperatures without proper normalization; conclusions like “surpassing all reported Cu-MOFs” are therefore too strong.

c. The 87 K TDS/selectivity value in the main text does not match the value in the SI; please unify.

d. Several captions (GCMC/DFT figures) do not state the exact T/P used, which makes reproduction difficult.

e. If the authors wish to keep the strong dual-site wording, they should add at least one site-resolving dataset (VT-DRIFTS with D_2, coverage-dependent calorimetry, or deconvoluted TDS peaks with activation energies- A single gas TDS will also be helpful.).

f. It seems typo “Cu-mbtc (0.154 cm3 m-2 for D_2 and 0.172 cm3 m-2 for H_2), “ should be other way around.

Reviewer #2

(Remarks to the Author)

The manuscript entitled "Dual-Orbital Synergy in Paired Cu(II) Open Metal Sites for Enhanced High-Temperature Hydrogen Isotope Separation" presents a carefully designed and well-executed study that advances the field of hydrogen isotope separation. The authors introduced a novel approach using Cu-ATC, a copper-based metal-organic framework featuring paired open metal sites. They demonstrated how dual-orbital synergy enables precise control over adsorption energetics and quantum sieving behavior.

The study employed a comprehensive suite of techniques including in situ DRIFTS, cryogenic temperature-desorption spectroscopy (TDS), column breakthrough experiments, and DFT calculations. These methods collectively provided compelling evidence for the proposed mechanism. The achieved D_2/H_2 selectivity of 20 at 50 K represents a significant advance for Cu(II)-based MOFs. The data are internally consistent and robustly support the proposed model.

A key strength of this work lies in its successful integration of fundamental orbital interactions with practical separation performance through systematic characterization. The authors demonstrated how spatially organized dual open metal sites enhance chemical affinity quantum sieving and thereby establish clear, evidence-based design principles. The concept of combining pore confinement with strategic open metal site positioning provides a valuable framework for rational material development that should benefit the broader research community.

This study represents an important contribution to both the mechanistic understanding of quantum sieving and the development of MOF materials capable of maintaining high selectivity at more practical operating temperatures. The work represents a valuable contribution to the field of hydrogen isotope separation. With some additional data and clarifications, this manuscript would be well-suited for publication in Nature Communications.

The following points should be addressed before acceptance:

1） The manuscript highlights the exceptional performance of Cu-ATC but does not thoroughly investigate the structural stability under multiple adsorption-desorption cycles or the framework's tolerance to potential impurities such as water and O_2. Could the authors provide data on the recyclability of Cu-ATC and discuss its chemical stability in practical operating environments?

2） The remarkable selectivity of 20 was achieved at 50 K, whereas the breakthrough experiment conducted at 77 K showed a significantly lower selectivity of 1.8. Could the authors discuss the energy penalty and technical feasibility of operating a large-scale separation unit at 50 K compared to 77 K? What is the primary factor responsible for this substantial selectivity drop over a 27 K temperature increase?

3） The study focuses on H_2/D_2 separation. Given the more pronounced quantum effects in heavier isotopes, does the dual-OMS strategy and the enhanced CAQS effect provide an even greater advantage for separating D_2/T_2 or H_2/T_2 mixtures, which are critically important for the nuclear industry?

4） In the TDS spectra of Cu-ATC at Texp = 26 K, H_2 desorption emerges anomalously earlier than D_2. This phenomenon is not observed in other materials or at other temperatures in this study. Could the authors provide a plausible explanation for this unique behavior in the main text?

5） Two software packages (Materials Studio and VASP) were employed in the computational section. Could the authors elaborate on how the consistency and reliability between these computational findings were ensured?

6） In the breakthrough experiments, Cu-mbtc exhibited a D_2/H_2 separation factor of 1.35, exceeding the value of 1.2 for Cu-bptc. This trend contradicts the observations from TDS measurements. Please provide a reasonable explanation for this apparent discrepancy.

7） It would be beneficial to include calculations of charge density difference and adsorption energy for H_2 in the VASP simulations. This would enable a direct comparative analysis with the corresponding results for D_2.

8） The authors should provide the purity information for all test gases used in the experiments.

9） Some physical quantities should be written in italics.

10) Reference format should be checked and corrected.

Reviewer #3

(Remarks to the Author)
The manuscript investigates hydrogen-isotope separation using Cu(II) paddlewheel MOFs and advances a clear design principle in which facing paired open-metal sites are embedded within ultramicroporous channels to enhance chemical-affinity quantum sieving. The concept is novel, as dual opposing OMSs create a confined interaction environment that strengthens CAQS through cooperative orbital engagement while preserving diffusion pathways. The methodology is tightly integrated, combining two closely related structural controls with TDS, column breakthrough, in situ DRIFTS, and GCMC/DFT to build a coherent structure-to-mechanism-to-performance narrative that connects macroscopic metrics with molecular-level insight. Especially, for Cu-ATC, DFT calculations confirmed the strong interaction between the σ orbital of hydrogen isotope molecules and both Cu dz2 orbital, which underscores both fundamental significance and application relevance. Therefore, Cu-ATC has meaningful selectivity and favorable kinetics persist near liquid-nitrogen temperature. I recommend publication in Nature Communications after minor revision.
1. At line 42, the authors mention utilizing quantum effects for hydrogen isotope separation but provide limited mechanistic detail. A more substantive discussion of the underlying physical principles governing these quantum effects would strengthen the introductory narrative and better contextualize the experimental strategy.
2. The Cu(I)-exchanged ZSM-5 system cited in line 54, which does not exhibit KQS–CAQS synergy, would be more appropriately repositioned within the section introducing CAQS effects, as this placement would enhance the logical flow of the mechanistic discussion.
3. To facilitate reader comprehension of the structural hierarchy, the authors should indicate the spherical diameter representing the intracage space in Figures 1e and 1f.
4. In the "Adsorption Mechanism Study" section, while charge density difference and electron density distribution were calculated for Cu-ATC, the authors should incorporate corresponding computations for Cu-bptc and Cu-mbtc. This addition would establish a parallel and more rigorous comparative framework across the three MOF systems.
5. In the "Adsorption properties of H2 and D2" section, while the authors present adsorption capacities normalized by specific surface area, a comparison based on adsorption capacity per open metal site would offer more direct mechanistic insight, given that OMS are the primary active centers governing isotope selectivity in this work.
6. In the analysis of the TDS spectra for Cu-bptc (Figures 4c, d), the first desorption peak is assigned to ultramicropores and the second to oxygen atoms. The authors should provide explicit justification for this assignment to ensure interpretive rigor. Possible supporting evidence could include computational energetics, site occupancy analysis, or previous literatures.
7. The TDS selectivity of both Cu-ATC and Cu-bptc shows a characteristic pattern of initial increase followed by decrease with rising exposure temperature. The authors should elucidate the underlying mechanism responsible for this behavior, as understanding this trend is important for optimizing operating conditions and clarifying the balance between kinetic and thermodynamic factors.

Reviewer #4

(Remarks to the Author)
The manuscript on "Dual-Orbital Synergy in Paired Cu(II) Open Metal Sites for Enhanced High-Temperature Hydrogen Isotope Separation" by Fenglei Wang et al. reports on gas adsorption isotherms, DRIFT spectroscopy, TDS measurements, breakthrough measurements and GCMC calculations for H2/D2 isotope separation. Several experimental details are missing, the interpretation and assignment of adsorption sites is not clear and not well supported by the experimental data.
1. Structure characterization: The BET area is analyzed measuring nitrogen adsorption isotherms. The IUPAC report DOI 10.1515/pac-2014-1117 suggest that preferentially Argon should be used, but nitrogen might be okay but with a higher uncertainty. However, the BET method is not valid for microporous materials, but can be applied using special criteria (so-called Rouquerol's criteria), see IUPAC report. The authors don't specify which pressure range has been applied and if the criteria are met. This should be checked and included in the SI.
2. Adsorption isotherms of H2 and D2: The uptake values are given in a ridiculous accuracy "high uptake capacities (q): 248.907 cm3 g-1 for D_2 and 219.596 cm3 g-1 for H_2". Then the authors discuss the H2 and D2 adsorption by comparing to the BET area obtained by N2. The different size of the molecules may easily cause different areas and pores accessible for each molecule, therefore, such a comparison is questionable. They mention "despite a comparable surface area to Cu-ATC, highlighting the adverse effect of channel expansion" but no proof for an expansion is given. In Figure 2(c) the adsorbed uptake is divided by the N2 BET area, which might be a quite random approach considering my previous arguments.
3. TDS measurements: The authors claim in the first sentence: "To identify adsorption sites in the three MOFs and their affinity for H2/D2, TDS measurements were conducted." TDS is a macroscopic measurement of the desorption rate during a constant heating in high vacuum, therefore, no assignment to a microscopic adsorption site is possible. Considering this, the assignment of the specific sites to different TDS maxima is done only by intuition and could be as well completely different or wrong. In Figure 4(a) the site I is even proposed to be occupied on opposite sides in the channel, a highly unlikely situation at relatively high temperature.
Figure 5 shows the dependence on exposure temperature, this should be clearly indicated on the x-axis. It is not described how the experiment is performed, is the evacuation of the non-adsorbed gas performed at the exposure temperature? Furthermore, the amount of D2 desorbed should be included either in Figure 5 or S10-12. For separation efficiency one should consider both selectivity and uptake.
Comparison to literature in Figure 6: For a proper comparison, the authors should give the references for the different materials. Here it would be important if for all the literature experiments the conditions of exposure pressure and time as well as evacuation temperature are comparable. In contrast, the authors state: "These findings underscore Cu-ATC's CAQS potential above liquid nitrogen temperatures, supporting its promise for industrial hydrogen isotope separation (Figure 6).", I cannot see this promise.

Summarizing, the results and data are not supporting the interpretation and don’t allow a sound discussion.

Version 1:

Reviewer comments:

Reviewer #1

(Remarks to the Author)
The revised manuscript is improved. The newly added D_2 DRIFTS results, the paired-site versus monomer DFT comparison, and the expanded methodological descriptions together provide a reasonably coherent basis for the proposed cooperative dual-site interpretation, even though the evidence remains indirect.

However, a few points still need correction :
(i) the D_2 red-shift values in the DRIFTS discussion should be checked carefully for consistency with the figure/table values;
(ii) it still does not fully explain why the selectivity reaches a distinct maximum specifically at 50 K. Since this is one of the most notable features of the data, the discussion should be expanded so that the reader can better understand the physical origin of this non-monotonic behavior.
(iii) the literature comparison should be softened because the compared data were obtained under non-identical conditions.

Reviewer #2

(Remarks to the Author)
It can be accepted.

Reviewer #3

(Remarks to the Author)
The authors have made satisfactory revisions to the manuscript and addressed the concerns I raised. I have no further comments and recommend acceptance of this article for publication in Nature Communications.

Reviewer #4

(Remarks to the Author)
The revised manuscript on Synergistic Effect of Paired Cu(II) Open Metal Sites for Enhanced High-Temperature Hydrogen Isotope Separation by Fenglei Wang et al. has improved in quality and clarity. Most of the reviewers’ questions have been satisfactorily answered and additional information is given.
Since the response to reviewers is typically published, the authors’ answers should be correct.
1) Reviewer #2’s question 3) is answered by “The difference in zero-point energy between T_2 and D_2 is greater than that between H_2 and D_2 [Nature Communications 8, 14496].” The difference of zero point energy depends on the mass difference between the isotopes, therefore, $H_2/D_2 = 2/4$ and $D_2/T_2 = 4/6$ and I cannot find this statement in the reference given.
2) 1) Reviewer #2’s question 4) is answered by “As generally observed, D_2 consistently desorbed earlier than H_2 as the temperature increased [Science 366, 613–620 (2019); Adv. Mater. 2019, 31, 1805293].” Typically, all TDS experiments show H_2 desorption maxima at lower temperature compared to D_2, again I cannot find this argument shown in the two references.

Version 2:

Reviewer comments:

Reviewer #1

(Remarks to the Author)
Now the authors have responded to all my concerns.

Reviewer #4

(Remarks to the Author)
The second revision of the manuscript on Synergistic Effect of Paired Cu(II) Open Metal Sites for Enhanced High-Temperature Hydrogen Isotope Separation by Fenglei Wang et al. has improved in quality and clarity, again.
However, authors’ response to the reviewer’s comment 2): “Reviewer #2’s question 4) is answered by “As generally observed, D_2 consistently desorbed earlier than H_2 as the temperature increased [Science 366, 613–620 (2019); Adv. Mater. 2019, 31, 1805293].” Typically, all TDS experiments show H_2 desorption maxima at lower temperature compared to D_2, again I cannot find this argument shown in the two references.”

The authors’ new answer is really a bit strange, since now the authors are no longer considering the main desorption maxima, but look at the tiny initial start of desorption. This little initial desorption is only around 1 % of the total desorption and 98 % are in the maxima which show the typical behavior first H_2 than D_2 desorption. Assigning 1 % of the total adsorbed amount to a specific adsorption site is simply impossible, therefore, such an analysis is unnecessary and doesn’t add any additional information to the paper. Furthermore, this very minor effect occurs at temperatures below 30 K, here the higher boiling point of D_2 compared to H_2 may cause this small prior desorption, by a very tiny amount of condensed D_2 in pores or inter-particle voids.

Version 3:

Reviewer comments:

Reviewer #4

(Remarks to the Author)
The authors answered all questions satisfactorily and clarified all points in a sound manner. The manuscript can be accepted in its present form.

Open Access This Peer Review File is licensed under a Creative Commons Attribution 4.0 International License, which permits use, sharing, adaptation, distribution and reproduction in any medium or format, as long as you give appropriate credit to the original author(s) and the source, provide a link to the Creative Commons license, and indicate if changes were made.
In cases where reviewers are anonymous, credit should be given to 'Anonymous Referee' and the source.
The images or other third party material in this Peer Review File are included in the article’s Creative Commons license, unless indicated otherwise in a credit line to the material. If material is not included in the article’s Creative Commons license and your intended use is not permitted by statutory regulation or exceeds the permitted use, you will need to obtain permission directly from the copyright holder.
To view a copy of this license, visit https://creativecommons.org/licenses/by/4.0/

Nature Communications manuscript NCOMMS-25-81623 Point-by-point response

We thank all reviewers for their valuable comments helping to improve the manuscript!

Reviewer #1 (Remarks to the Author):

The manuscript claims that deuterium/hydrogen isotope separation is amplified by a dual-Cu open-metal-site (OMS) arrangement in an ultramicroporous Cu-MOF. However, the experimental evidence provided does not directly demonstrate that two adjacent OMSs cooperatively bind a single H_2/D_2 molecule, and several datasets that should track smoothly with this hypothesis show non-monotonic or even contradictory behavior. Because the central mechanistic claim is insufficiently supported and several key figures contain internal inconsistencies, I cannot recommend publication in the present form.

Major comments：

1. The main point of this paper is that "dual (paired) Cu OMSs" improve the adsorption strength and thus enhance D_2/H_2 selectivity. However, the manuscript does not provide any experiments that directly demonstrate two cooperative Cu sites binding the same molecule. The only site-discriminating measurement is the low-temperature temperature-programmed desorption (TDS), which shows multiple desorption features. This only indicates that "more than one type of site exists (e.g. weak and strong site)," rather than proving that "two Cu sites act synergistically on one adsorbate." All other evidence presented (such as the higher heat of adsorption (Qst) for Cu-ATC, the red shift observed in DRIFTS at 298 K, GCMC/DFT maps, and breakthrough selectivity) is indirect and could also support a simpler explanation: that "Cu-ATC contains one relatively strong OMS located in a narrow one-dimensional channel."

Answer: We sincerely thank the reviewer for this insightful comment. We fully agree that the experimental results in the original manuscript, including TDS, DRIFTS, and calculated Q_{st}, can distinguish the relative strength of adsorption sites but do not by themselves directly prove that one hydrogen/ deuterium(H_2/D_2) molecule simultaneously binds to two Cu(II) OMSs.

To address this concern, we performed more comprehensive and accurate calculations using VASP to rigorously examine the origin of the enhanced adsorption in Cu-ATC(shown in Figure 1a). Specifically, we compared the H_2/D_2 adsorption energies at the primary adsorption site in the three MOFs.

The results of calculations show that Cu-ATC exhibits significantly higher adsorption energies for H_2 (–9.07 kJ·mol^{-1}) and D_2 (–13.00 kJ·mol^{-1}) compared to Cu-bptc (H_2: –6.42 kJ·mol^{-1}; D_2: –9.61 kJ·mol^{-1}) and Cu-mbtc (H_2: –6.90 kJ·mol^{-1}; D_2: –10.12 kJ·mol^{-1}). Importantly, when the interaction is restricted to a single paddlewheel-type Cu(II) site in an isolated paddlewheel structural unit of Cu-ATC (shown in Figure 9b), the adsorption energies decrease to –6.16 kJ·mol^{-1} for H_2 and –9.62 kJ·mol^{-1} for D_2, which are essentially comparable to those of Cu-bptc and Cu-mbtc. Moreover, the charge density difference maps (Figure 9) show that in Cu-ATC, the adsorbed H_2/D_2 molecules undergo orbital interactions with the Cu open metal sites on both sides, consistent with a cooperative paired-sites adsorption mode.

Taken together, these results strongly support that the enhanced adsorption in Cu-ATC might originate from the cooperative effect of closely spaced paired Cu sites, rather than from a single strong Cu site in a narrow channel.

Accordingly, we have revised the relevant text in the manuscript to avoid overstatement and to clarify that our conclusion is based on a combination of adsorption-energy comparison, single-site control calculations, and charge density difference analysis.

In Revised Manuscript on Page 19, 20.

From:

"Cu-ATC's primary Site I (between adjacent paddlewheels) yielded a H_2 adsorption energy of -11.01 kJ mol^{-1}, matching experimental Q_{st}, far stronger than the values at the comparable OMS in Cu-bptc (-8.52 kJ mol^{-1}) and Cu-mbtc (-7.30 kJ mol^{-1}). This enhancement arises from Cu-ATC's short Cu-Cu distance (5.98 Å)."

To:

"The calculated H_2/D_2 adsorption energies at the primary adsorption site I (between adjacent paddlewheels, shown in Figure 9a) of Cu-ATC were -9.07 kJ mol^{-1} and -13.00 kJ mol^{-1}, respectively, which are markedly stronger than those of Cu-bptc (H_2: -6.42 kJ mol^{-1}, D_2: -9.61 kJ mol^{-1}) and Cu-mbtc (H_2: -6.90 kJ mol^{-1}, D_2: -10.12 kJ mol^{-1}). To distinguish the paired-site effect from a single-site effect, we further calculated the adsorption energies for H_2/D_2 on an isolated single paddlewheel-type Cu(II) site of Cu-ATC (Figure 9b). The resulting adsorption energies (H_2: -6.16 kJ mol^{-1}, D_2: -9.62 kJ mol^{-1}) were comparable to those of Cu-bptc and Cu-mbtc. More importantly, the calculated vibrational frequencies of adsorbed H_2/D_2 in the three MOFs (shown in Table 1) were in good agreement with the experimental values. These computational results suggest that the enhanced affinity of Cu-ATC for hydrogen isotopes cannot be attributed to an individual Cu site alone, but is instead associated with the closely spaced Cu···Cu arrangement (5.98 Å). Consistent with this interpretation, the charge density difference maps (Figure 10) reveal electron redistribution involving the adsorbed hydrogen isotope molecule and the two closely spaced Cu open metal sites in Cu-ATC, while the corresponding single-site model as well as Cu-bptc and Cu-mbtc do not exhibit the same dual-site interaction feature. Taken together, these results support the conclusion that the enhanced adsorption strength of Cu-ATC arises from a cooperative effect associated with the paired Cu OMSs."

In Revised Manuscript at Page 20, 21.

Added Figure 9

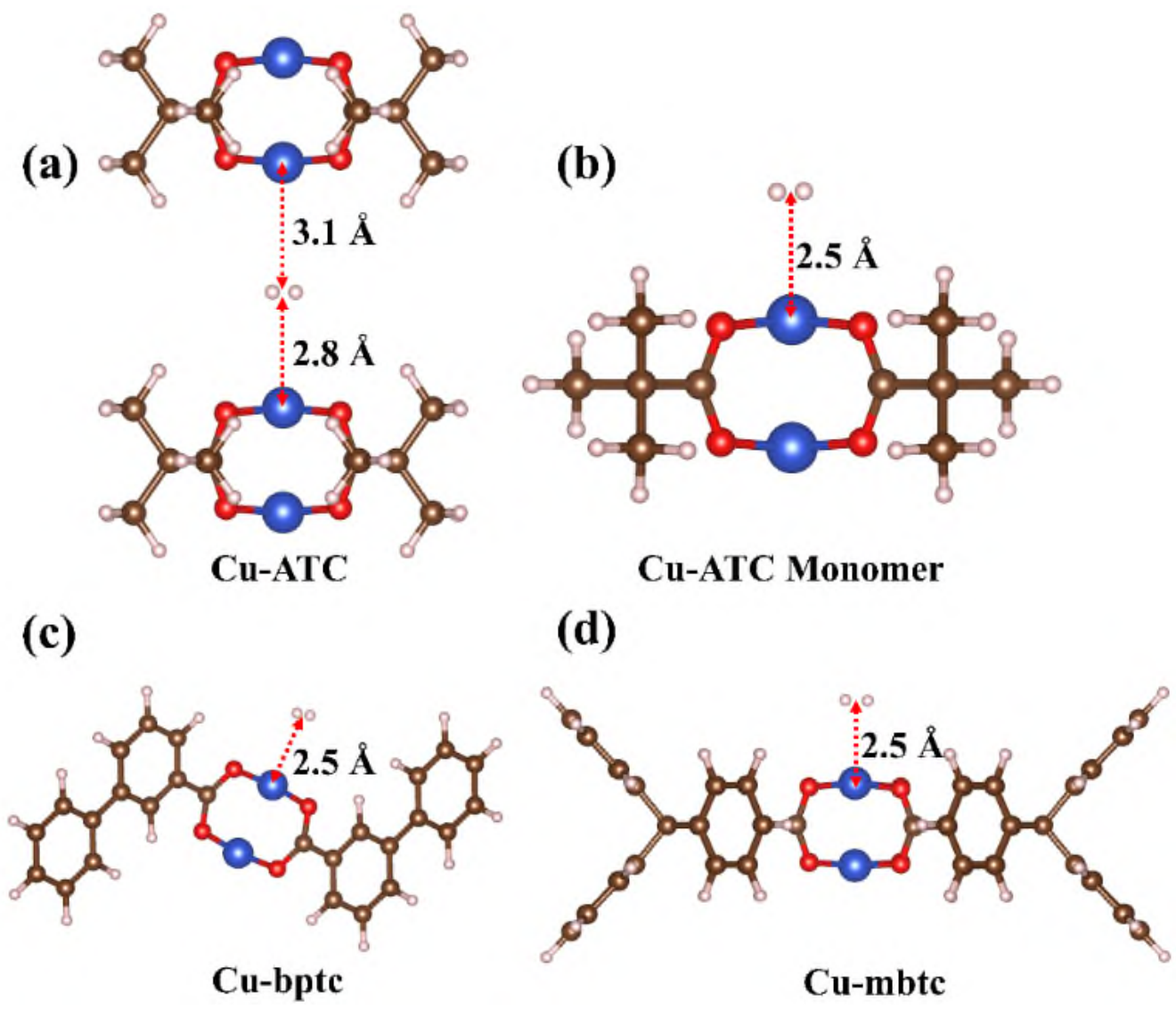

Figure 9. Structure of finite model of (a) Cu-ATC, Cu-ATC Monomer(c) Cu-bptc, and (d) Cu-mbtc as optimized by DFT and corresponding equilibrium position of the hydrogen molecule adsorbed at the Cu(II) atom. (blue, copper; red, oxygen; tan, carbon; pink, hydrogen)

Added Table 1

Structures	H_2	H_2 red shift	D_2	D_2 red shift
Cu-ATC	3931 (3958) cm^{-1}	230 (203) cm^{-1}	2830 (2846) cm^{-1}	163 (147) cm^{-1}
Cu-bptc	4011 (4019) cm^{-1}	150 (142) cm^{-1}	2886(2888) cm^{-1}	107 (105) cm^{-1}
Cu-ATC monomer	4011 cm^{-1}	152 cm^{-1}	2884 cm^{-1}	109 cm^{-1}

Table 1. The DFT-computed vibrational frequencies of H_2 and D_2 adsorbed on Cu-ATC, Cu-bptc, and the Cu-ATC monomer , with experimental values given in parentheses.

Updated Figure 10

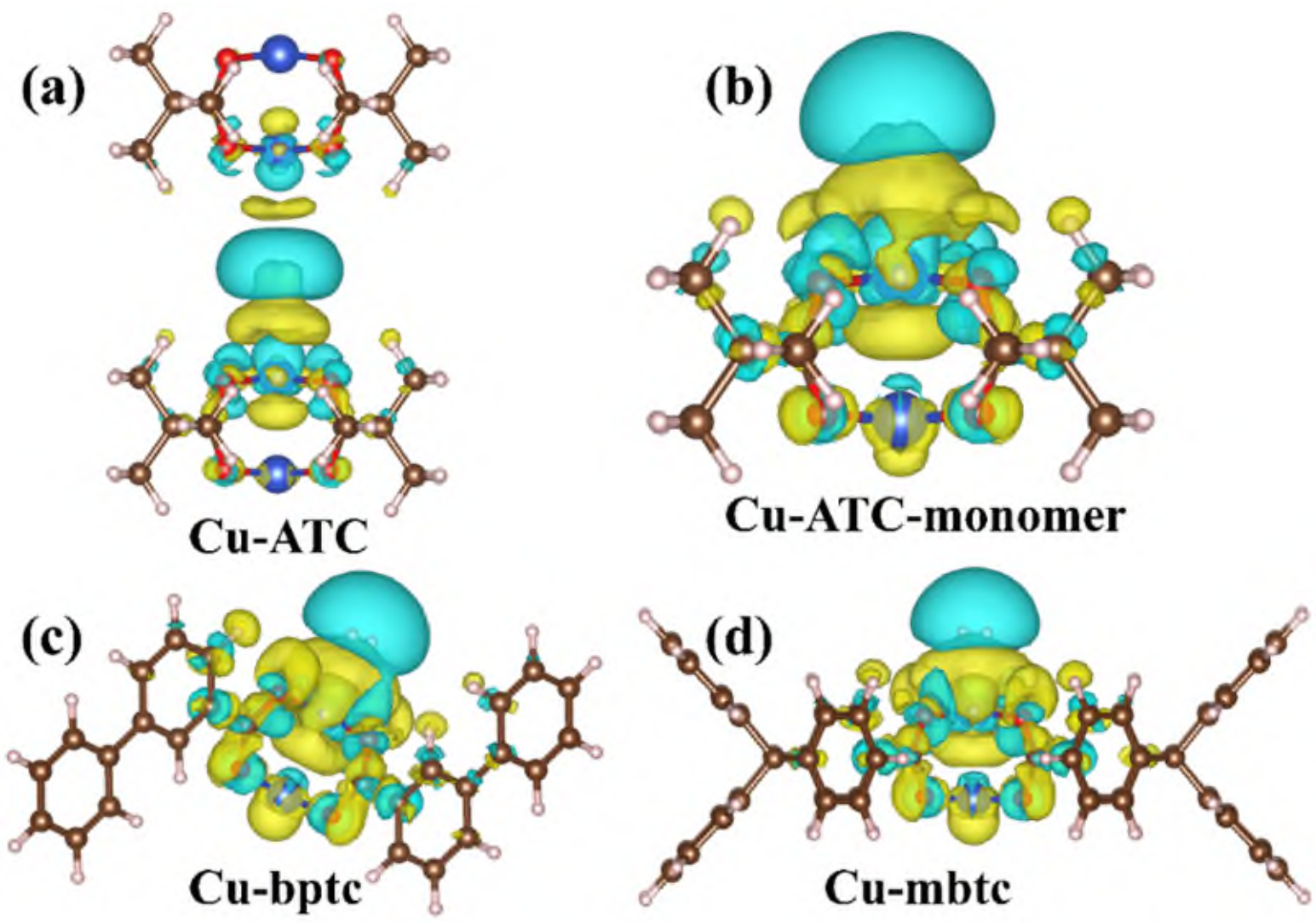

Figure 10. The 3D charge density difference distribution of (a) between the Cu OMSs of adjacent paddlewheels in Cu-ATC and about a single Cu OMS in Cu-ATC-monomer (b), Cu-bptc (c), Cu-mbtc (d) as determined from simulation. The yellow zone and cyan zone denote the charge accumulation and depletion.

Similarly, the unusually large red shift (≈682 cm⁻¹) observed only for Cu-ATC is used as a key argument for "strong, cooperative" binding. However, (i) the shift is very large for RT H_2 on Cu(II) OMS; (ii) there is no D_2 analogue to demonstrate isotope-sensitive binding at the same site; and (iii) Cu-bptc and Cu-mbtc show essentially no signal under otherwise identical conditions. In its current form the DRIFTS result shows "Cu-ATC has a strong site," not "Cu-ATC has two Cu sites acting together."

Answer:

We sincerely thank the reviewer for this valuable comment and fully agree that the original room-temperature DRIFTS data, particularly the unusually large red shift initially assigned to adsorbed H_2 in Cu-ATC, were not sufficient to support a conclusion of cooperative paired-sites binding. In particular, the absence of corresponding D_2 data and the lack of detectable signals in Cu-bptc and Cu-mbtc under the original conditions made the interpretation overly strong.

After carefully re-examining the original room-temperature measurement, we found that the

initially assigned band was likely affected by temperature fluctuation during H_2 dosing, which may have led to an inaccurate assignment. To address this issue, we have performed new *In situ* DRIFTS measurements at 130 K for H_2 and D_2 adsorption on Cu-ATC, Cu-bptc, and Cu-mbtc using a more precise instrument(shown in SI Section 2), and the data are now presented in the revised Figure 3. The results indicated that no detectable signals for adsorbed H_2 or D_2 were observed for Cu-mbtc, which was consistent with its weaker confinement effect toward hydrogen isotopes. In contrast, the characteristic bands of adsorbed H_2 and D_2 on Cu-ATC and Cu-bptc were observed. For H_2 adsorption, the bands appeared at 3958 cm^{-1} for Cu-ATC and 4019 cm^{-1} for Cu-bptc. Relative to gaseous H_2 (4161 cm^{-1}), these correspond to red shifts of 203 and 142 cm^{-1}, respectively, indicating a stronger interaction of H_2 with Cu-ATC. For D_2 adsorption, the bands appeared at 2846 cm^{-1} for Cu-ATC and 2888 $cm^{-1}$for Cu-bptc. Relative to gaseous D_2 (2993 cm^{-1}), these correspond to red shifts of 147 and 105 cm^{-1}, respectively, indicating a stronger interaction of D_2 with Cu-ATC.

To further support the assignment, we also performed DFT calculations of the vibrational frequencies of adsorbed H_2/D_2 in the three MOFs (shown in figure 9). The calculated results (shown in Table 1) were in good agreement with the experimental values, supporting that the stronger interaction of H_2/D_2 with Cu-ATC might originate from the cooperative effect of closely spaced paired Cu sites, rather than from a single strong Cu site in a narrow channel.

Importantly, we have revised the manuscript to avoid overstating what *In situ* DRIFTS alone can establish: the updated DRIFTS results do not by themselves directly prove simultaneous cooperative binding of one molecule by two Cu sites, but, together with the adsorption-energy calculations and charge density difference analysis, they are consistent with a paired-site contribution to the enhanced adsorption in Cu-ATC.

The revised content in the manuscript is presented below：

In Revised Manuscript on Page 11, 12.

Updated Figure 3.

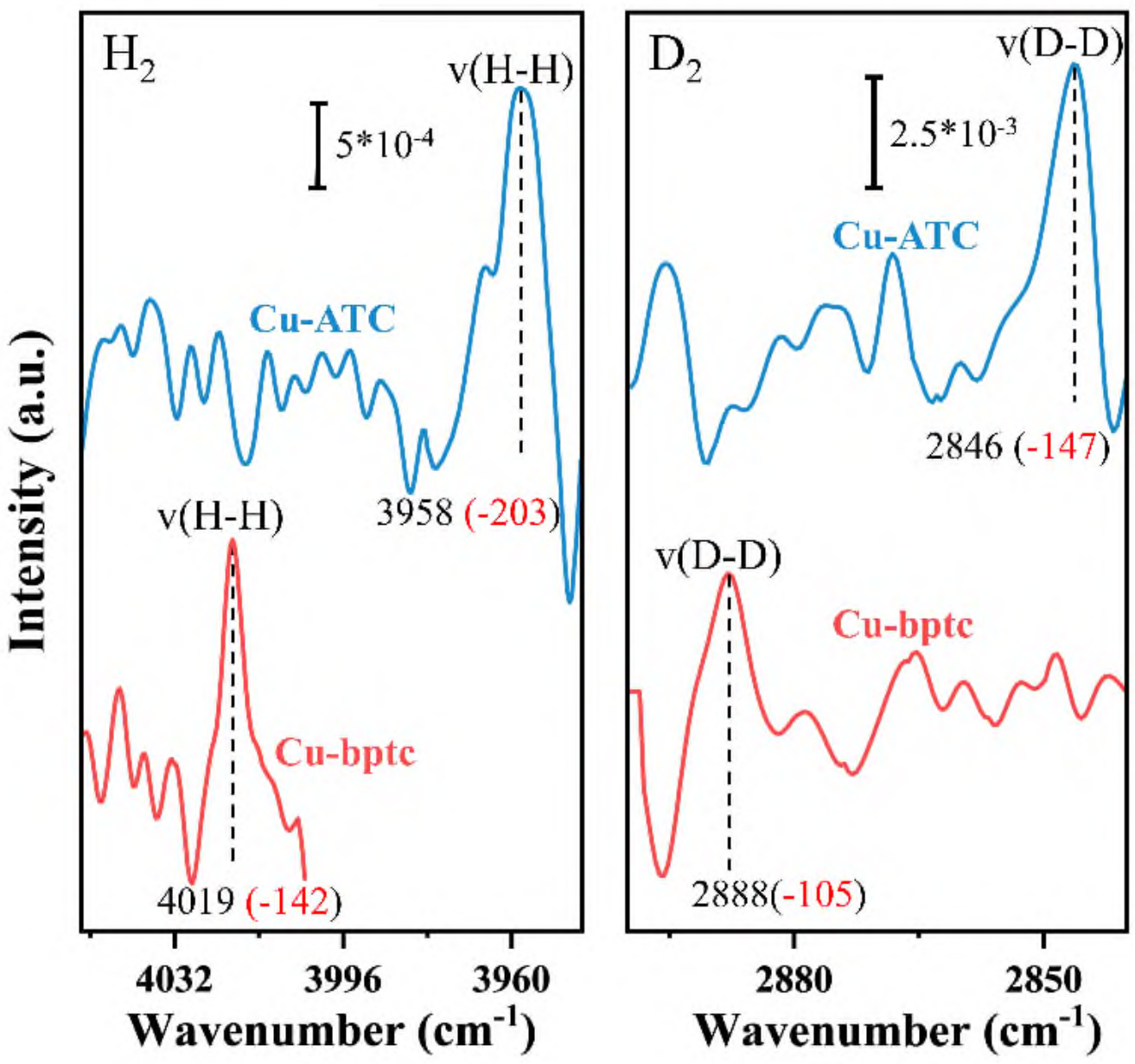

Figure 3. *In situ* DRIFT spectra of adsorbed H_2 and D_2 in the activated Cu-ATC (blue)/Cu-bptc (red). H_2/D_2 Gas was loaded at 130 K/1 atm.

Structures	H_2	H_2 red shift	D_2	D_2 red shift
Cu-ATC	3931 (3958) cm^{-1}	230 (203) cm^{-1}	2830 (2846) cm^{-1}	163 (147) cm^{-1}
Cu-bptc	4011 (4019) cm^{-1}	150 (142) cm^{-1}	2886(2888) cm^{-1}	107 (105) cm^{-1}
Cu-ATC monomer	4011 cm^{-1}	152 cm^{-1}	2884 cm^{-1}	109 cm^{-1}

Table 1. The DFT-computed vibrational frequencies of H_2 and D_2 adsorbed on Cu-ATC, Cu-bptc, and the Cu-ATC monomer , with experimental values given in parentheses.

From：

“To probe the H_2 adsorption mechanism in the MOFs, a stepwise hydrogen dosing protocol (2.6 mL min^{-1} per dose) was employed with *In situ* DRIFTS monitoring at room temperature (298 K) (Figure 3). In Cu-ATC, increasing H_2 exposure produced a progressively intensified broad absorption band centered at 3479 cm^{-1}, red-shifted by 682 cm^{-1} relative to free H_2 vibrations (~4161 cm^{-1}). [33,34] This substantial shift confirms a strong interaction between H_2 and OMSs, supporting dihydrogen bond formation even at ambient temperature. In contrast,

Cu-bptc and Cu-mbtc showed no detectable H_2-related IR signals (Figure 3b and c), indicating negligible chemisorption at 298 K. This observation is consistent with Cu-ATC's elevated adsorption enthalpies (Q_{st} > 12 kJ mol^{-1}), highlighting topological differences: Cu-ATC's ultramicroporous one-dimensional channels (~5.6 Å) facilitate optimized H_2 interactions with paired OMSs (symmetric d_z^2 orbitals), whereas the larger cavities/channels (>8 Å) in Cu-bptc and Cu-mbtc allow molecules to bypass OMSs, weakening the interaction. These DRIFTS results directly substantiate Cu-ATC's CAQS advantage through structural synergy, extending the applicability of copper paddlewheel MOFs under mild conditions."

To:

"To probe the H_2/D_2 adsorption mechanism in the MOFs, *in situ* DRIFTS measurements of H_2/D_2 adsorption were conducted at 130 K (Figure 3). Distinct adsorption bands of H_2 and D_2 were observed for Cu-ATC and Cu-bptc, appearing at 3958/4019 and 2846/2888 cm^{-1} (Figure 3), respectively. In contrast, no detectable signals for adsorbed H_2 or D_2 were observed on Cu-mbtc, which is consistent with its weaker confinement effect and less favorable adsorption environment for hydrogen isotopes.

Relative to gaseous H_2 at 4161 cm^{-1}, the H–H stretching vibration (v(H-H)) is red-shifted by 203 cm^{-1} for Cu-ATC and 142 cm^{-1} for Cu-bptc, indicating a stronger interaction in Cu-ATC. For D_2 adsorption, compared to gaseous D_2 at 2993 cm^{-1}, the corresponding red shifts are 169 cm^{-1} for Cu-ATC and 147 cm^{-1} for Cu-bptc, following the expected isotopic trend. Although both Cu-ATC and Cu-bptc feature identical paddlewheel-type Cu(II) OMS and comparable pore apertures, the more pronounced red shifts observed for Cu-ATC suggest a stronger adsorption environment, which comes from the influence of Cu-ATC's ultramicroporous one-dimensional channels (~5.98 Å) and closely spaced Cu sites."

2. The highest reported separation factor occurs at 50 K. In a typical "two-site" mechanism, where the population of strong and weak sites gradually changes with temperature, we would expect a more consistent or smoothly varying selectivity. However, the data reveal a significant peak specifically at 50 K.

Answer: We greatly appreciate the reviewer's comment and fully agree with the suggestion. To better resolve the temperature dependence of the separation behavior, we performed additional TDS selectivity measurements at 40 K and 60 K, and the results are shown in Figure 5. As the exposure temperature increased from 26 K to 50 K, the TDS selectivity increased, whereas a further increase from 50 K to 87 K led to a gradual decrease. This behavior was also observed in the following reference. [***J. Am. Chem. Soc. 2017, 139, 15135−15141; J. Mater. Chem. A, 2020, 8, 6319–6327]***.

The revised content in the manuscript is presented below:

In supporting information on page S22.

Added Figure S13.

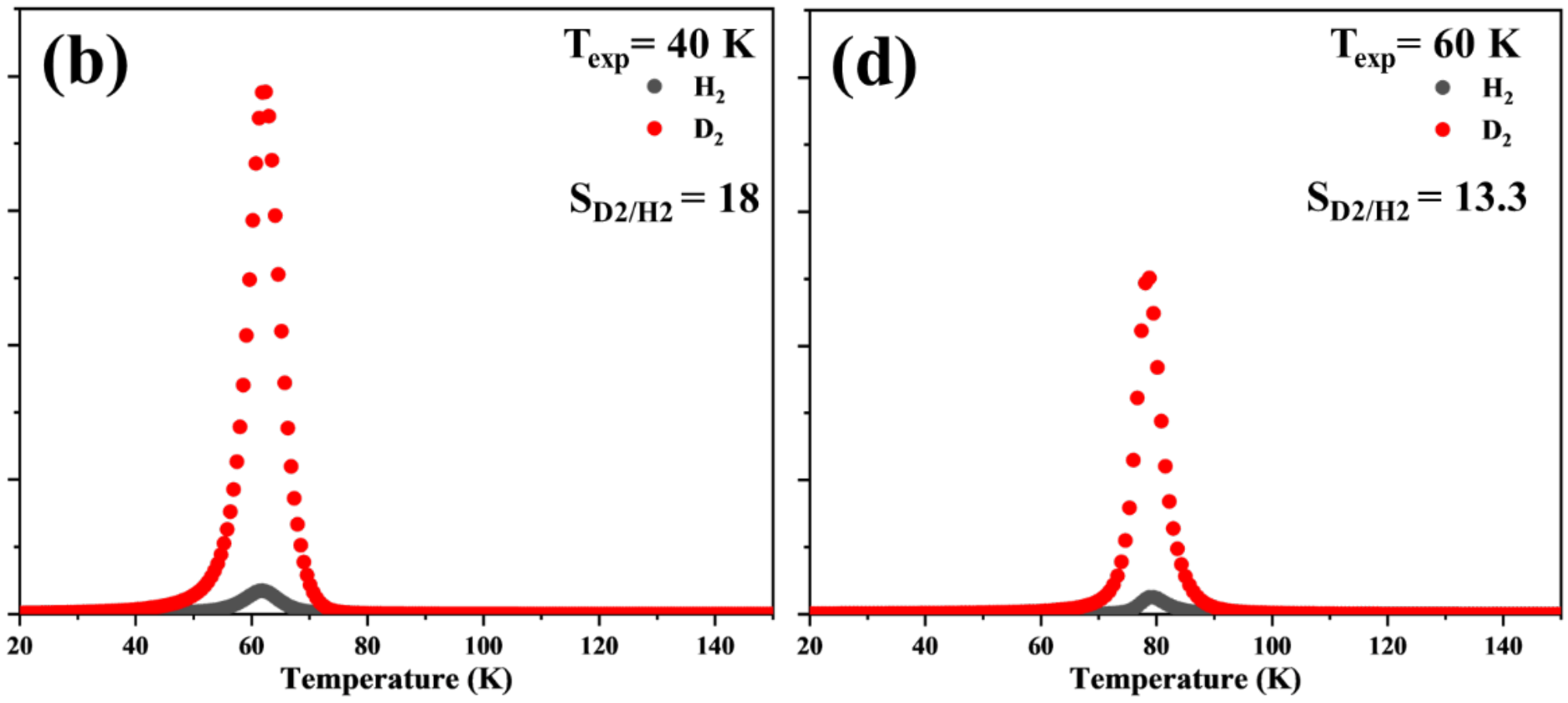

In Revised Manuscript on Page 15.

Updated Figure 5.

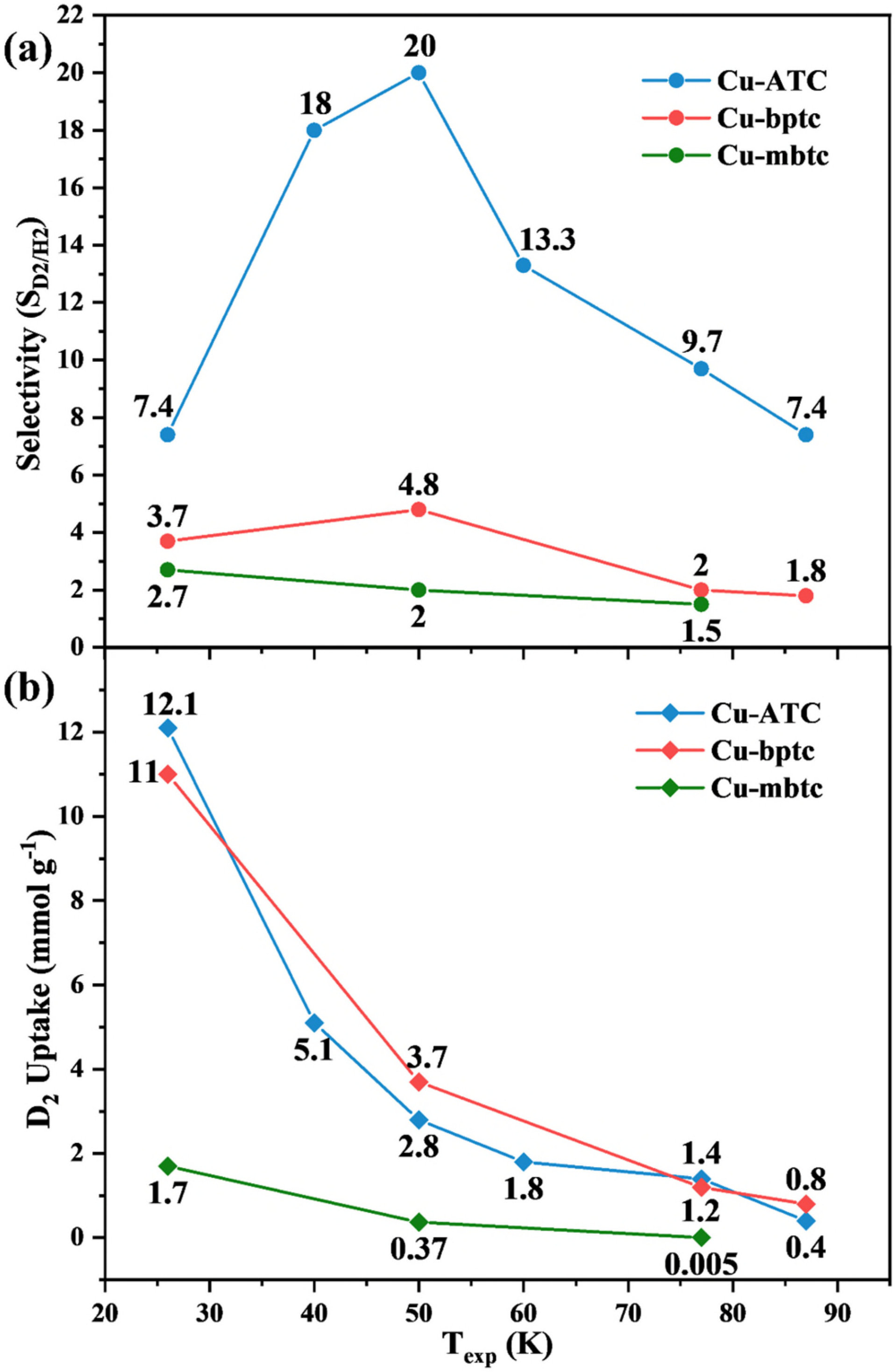

Figure 5. Adsorptive separation for an equimolar D_2/H_2 isotope mixture. (a) Selectivity for D_2 as compared to H_2 and (b) D_2 uptake of Cu-ATC (blue), Cu-bptc (red), and Cu-mbtc (green) as a function of T_{exp}.

3. In Fig. 2(a–c), the H_2 and D_2 uptakes of the three materials are very close at 77/87 K

(differences are small). However, in Fig. 2(d) the loading trace/line for D_2 is visibly much longer (roughly twice) than that for H_2, for all three materials. This is not consistent with the preceding panels.

Answer: We are grateful for the reviewer's suggestions. We agree that our original figure 2d could be misleading. This issue arose because the x-axis of the Q_{st} plot was originally expressed in mg/g. Although the adsorbed volumes of H_2 and D_2 in the material were similar, the mass of D_2 is twice that of H_2, which made the D_2 curve appear substantially longer than the H_2 curve. To improve the clarity of the figure, we recalculated the data and replotted the Q_{st} curves using mmol/g as the x-axis unit. The revised figure 2d has been updated in the manuscript accordingly.

In Revised Manuscript on Page 8.

Updated Figure 2(d).

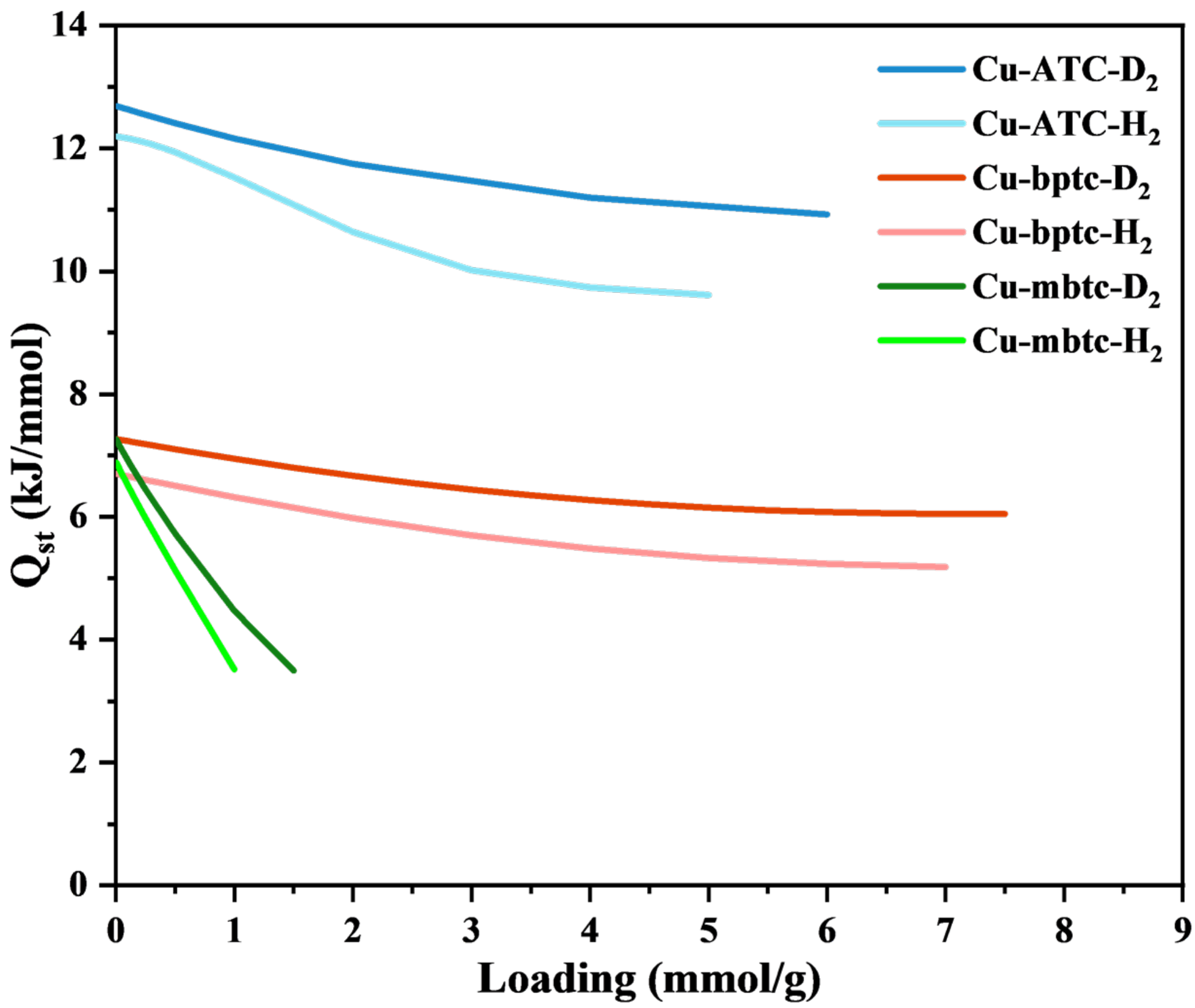

4. The TDS data appear to suffer from a procedural issue. In the SI, the exposure temperature and the TDS onset/peak temperatures do not line up consistently, and this mismatch is especially visible for the higher-temperature exposures. This is a well-known artefact in low-temperature isotope TDS: if, after high-T exposure, the sample is pumped under vacuum for too long at (or near) the exposure temperature, a non-negligible fraction of the more weakly bound hydrogen will already desorb during the pumping step. What remains on the sample when the actual TDS ramp starts is then an enriched, slower-desorbing fraction, so the apparent D_2/H_2 selectivity looks better than it really is.

- *Temperature-dependent pumping time must be defined and justified.*

- *Show reproducibility*

- *Align exposure T and TDS onset.*

Answer: We sincerely thank the reviewer for the insightful comments regarding the TDS experiments. The reviewer's concern that the evacuation step after D_2/H_2 exposure may introduce errors is well taken, as partial desorption of weakly adsorbed gas molecules during this step can indeed affect the selectivity. To address this, we standardized the post-adsorption procedure following established protocols in the literature ***[Science 366, 613–620 (2019); J. Am. Chem. Soc. 2017, 139, 15135−15141]***. Specifically, after gas exposure, the residual gas was evacuated using a molecular pump for a fixed duration of 10 min, after which the temperature was rapidly cooled to 20 K at a rate of 15 K/min. To evaluate the reproducibility, we repeated the TDS measurement for Cu-ATC under the same conditions (50 K, 10 mbar) six months after the initial experiment. The results shown in reviewer-only figure 1, demonstrated good reproducibility. Regarding the observed misalignment between exposure temperature and desorption onset temperature, we noted that similar behavior has been reported in previous low-temperature isotope TDS studies shown in reviewer-only figure 2. Although such misalignments are difficult to eliminate completely, the use of a fixed and consistent experimental protocol helps minimize the resulting systematic deviation.

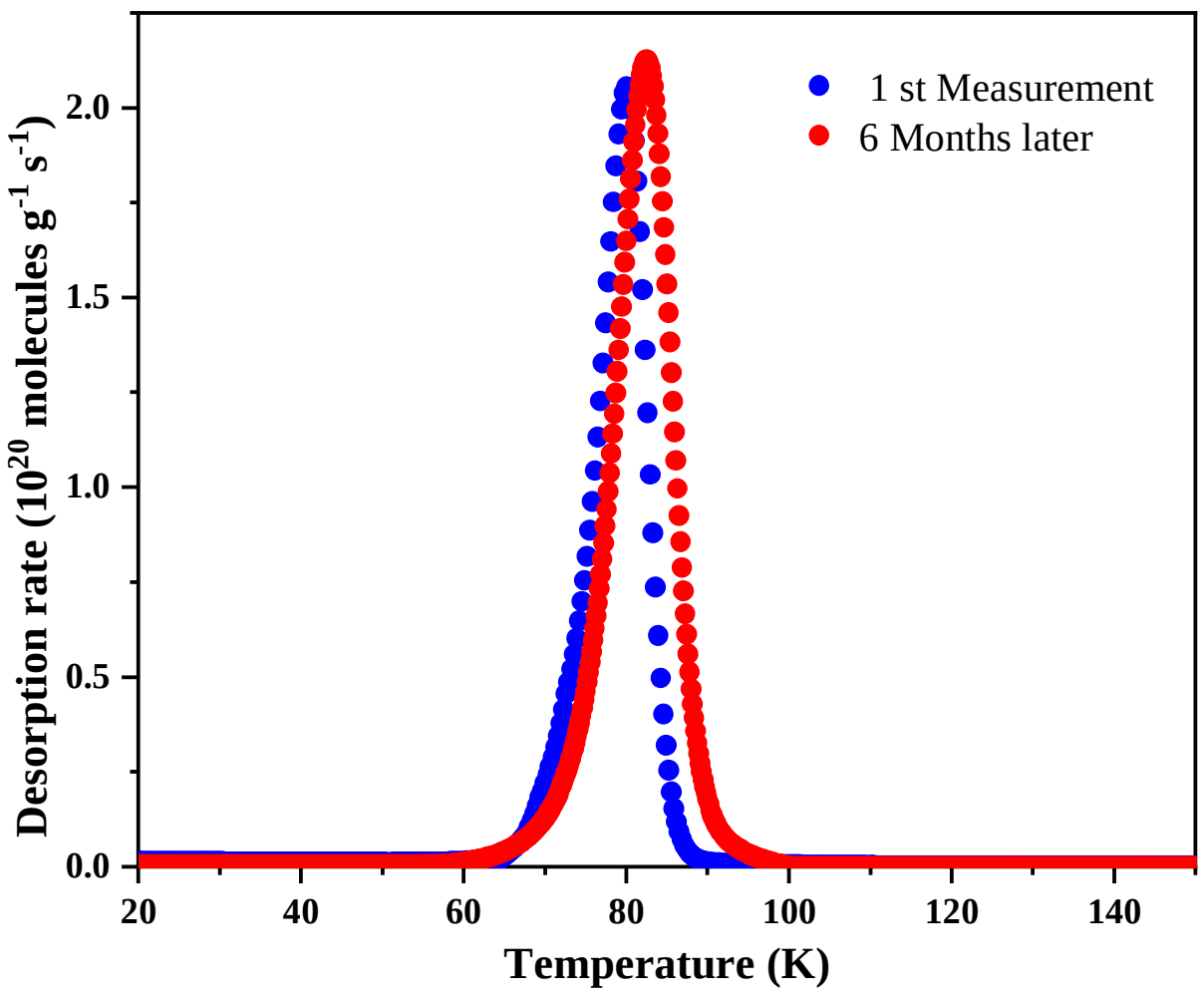

Reviewer’s only figure 1.

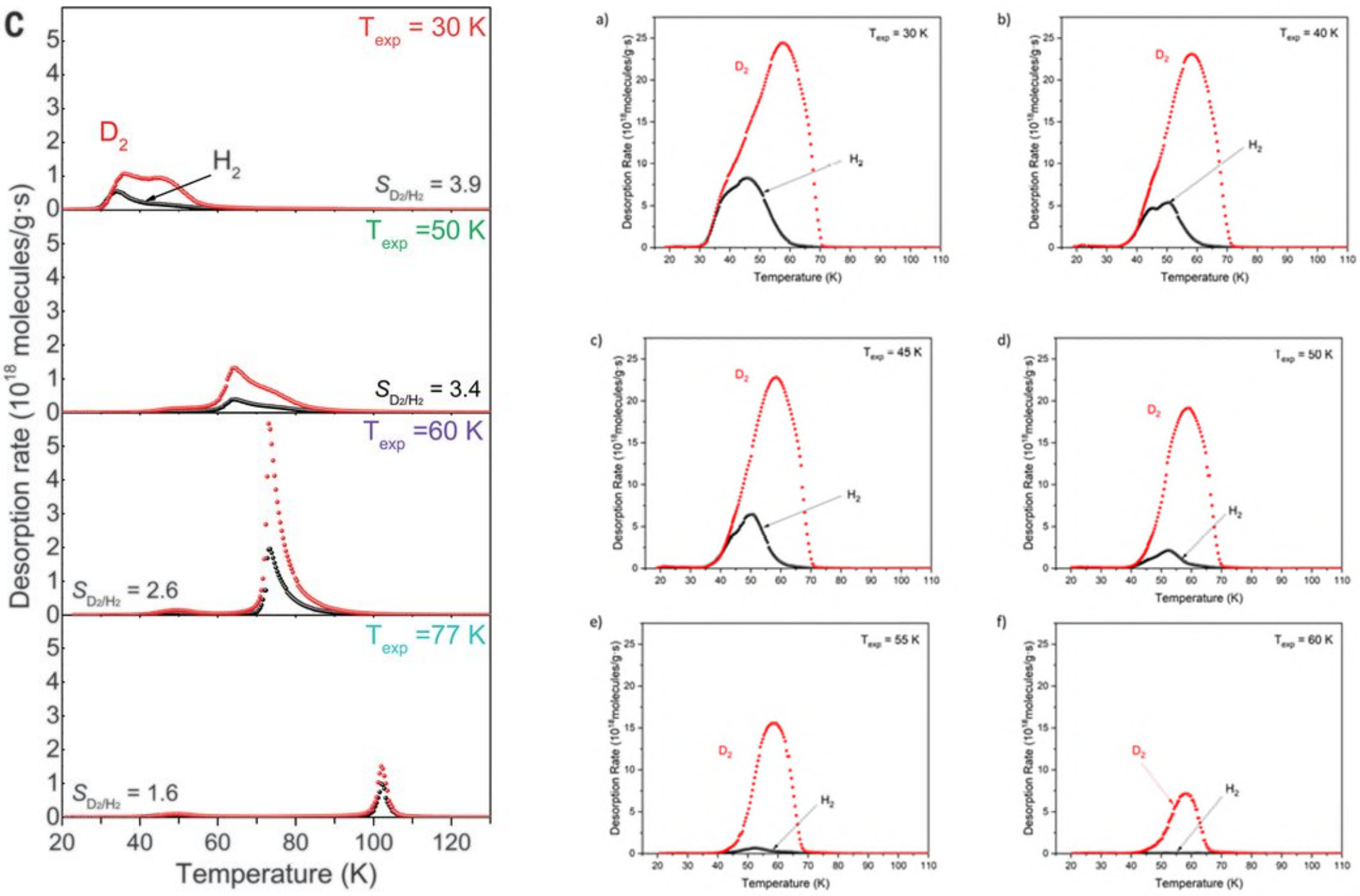

Science 366, 613–620 (2019) ***Nature Communications | (2025) 16:5941***

Reviewer’s only figure 2.

5. The manuscript reports IAST-based D_2/H_2 selectivities (e.g. Cu-ATC: 1.84 at 77 K; 1.60 at 87 K) only in the main text, but provides no description of the actual IAST procedure. Specifically, it is not stated which isotherm model(s) were fitted to the single-component H_2 and D_2 data (Langmuir, dual-site Langmuir, Langmuir–Freundlich, or another form), the fitted parameters, goodness of fit, and the coverage/pressure ranges used for the fits are not given,

the composition and total pressure used for the IAST prediction (nominally "equimolar") are not cross-checked against the experimental mixture used in TDS and breakthrough, no IAST curves (selectivity vs pressure, or vs loading) are shown for the three MOFs.

Answer: We sincerely thank the reviewer for this valuable comment. We agree that the description of the IAST analysis in the original manuscript was not sufficiently detailed. In response, we have recalculated the IAST selectivities in a more rigorous and comprehensive manner and have substantially supplemented the computational details in the Supporting Information. The revised calculation results and details are provided below.

In Revised Manuscript on Page 8, 9.

From:

"Ideal adsorbed solution theory (IAST) selectivity for D_2/H_2 (equimolar mixture), computed using a dual-site Langmuir-Freundlich model, was 1.84 for Cu-ATC at 77 K, surpassing those of Cu-bptc (1.42) and Cu-mbtc (1.42). At 87 K, Cu-ATC retained 1.60, while the others dropped to 1.17 and 1.38. These results affirm Cu-ATC's superior separation performance under mild conditions."

To:

"Ideal adsorbed solution theory (IAST) selectivity for an equimolar D_2/H_2 mixture, calculated using a dual-site Langmuir-Freundlich model, was 1.50 for Cu-ATC at 77 K, 101 kPa, surpassing those of Cu-bptc (1.45) and Cu-mbtc (1.43). At 87 K, 101 kPa, the selectivity of Cu-ATC remained 1.35, higher than that of Cu-bptc and Cu-mbtc (1.22). These results indicate Cu-ATC maintains superior D_2/H_2 separation performance under mild conditions."

Updated Figure 2

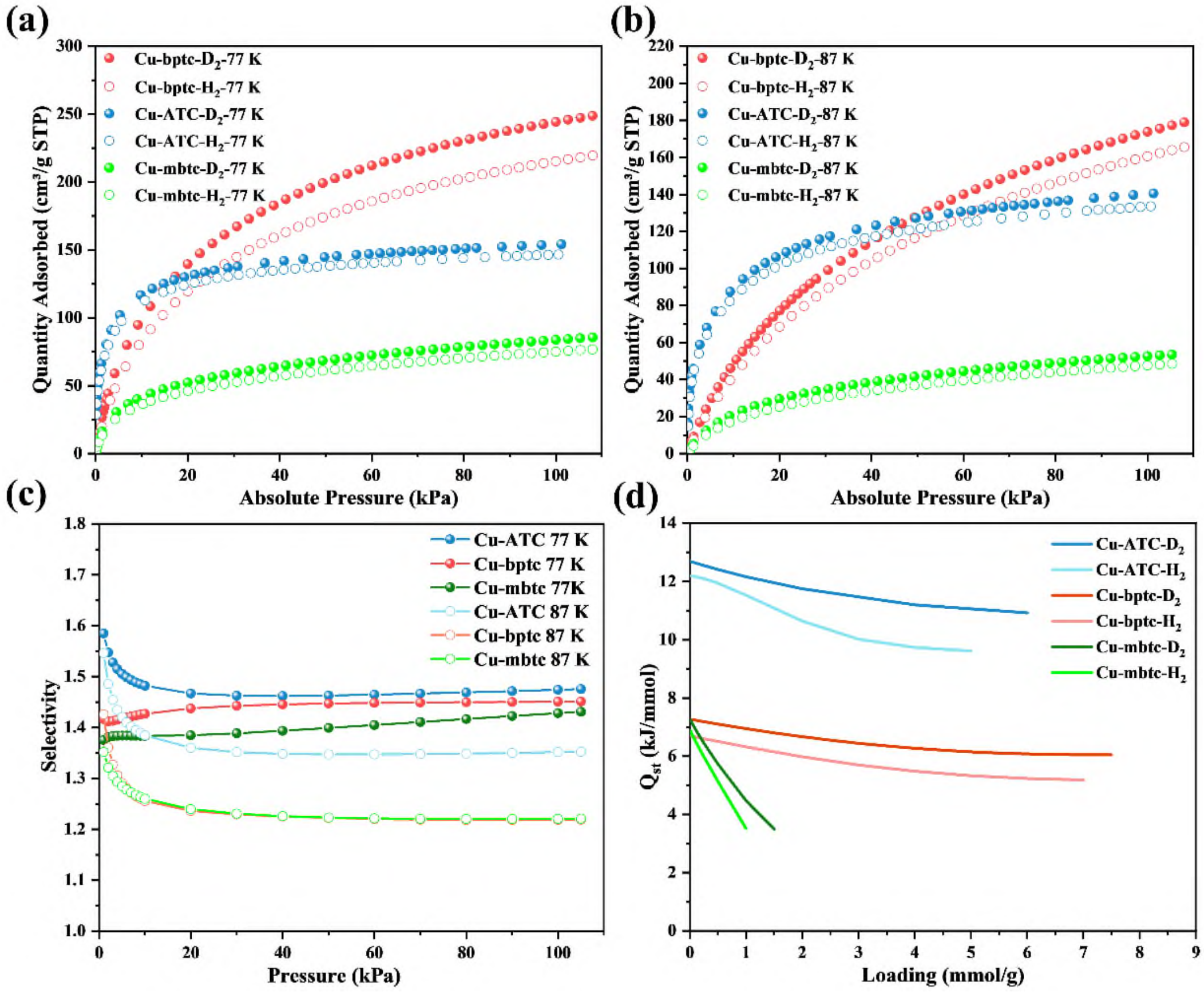

Figure 2. Single-component H_2 and D_2 adsorption isotherms of Cu-ATC (blue), Cu-bptc (red), and Cu-mbtc (green) at 77 K (a) and 87 K (b). IAST selectivity of Cu-ATC, Cu-bptc and Cu-mbtc for 1:1 D_2/H_2 mixture at 77 K and 87 K. (c). The isosteric heat of D_2 and H_2 for Cu-ATC, Cu-bptc, and Cu-mbtc (d).

In supporting information on page S6, S7, S8.

Updated contents:

Selectivity by IAST

Ideal adsorption solution theory (IAST) was calculated to evaluate the hydrogen isotope capture performance. [4] Double-site Langmuir (DSL) equation was first employed to fit the single-component gas equilibrium adsorption isotherms of MOF over the full pressure range. DSL equation can be written as:

$$N = A_1 \frac{b_1 p}{1 + b_1 p} + A_2 \frac{b_2 p}{1 + b_2 p}$$

Where N is the adsorption capacity (mmol g^{-1}), p is the pressure of bulk gas at equilibrium with adsorbed phase (bar), A_1 and A_2 are the saturation loadings for sites 1 and 2, respectively. b_1 and b_2 are the Langmuir parameters for sites 1 and 2. The parameters of A_1, A_2, b_1 and b_2 were then used to predict the adsorption selectivity based on IAST, which is finally defined as:

$$S = \frac{x_1/x_2}{y_1/y_2}$$

Where S represents the ideal selectivity of component 1 over component 2, x_i x_1 and y_i are the adsorbed phase mole fractions and the gas phase mole fractions, respectively.

Adsorption Capacity Calculation Based on Breakthrough Experiments

On the basis of mass balance, the gas adsorption amounts based on breakthrough experiments can be defined as follows [5] :

$$q_i = \frac{a_i V}{m \times V_m} \int_0^t \left(1 - \frac{y_i}{y_0}\right) dt$$

Where qi is the equilibrium adsorption amount of component i (mmol g^{-1}), ai is the percent of component i in the mixture, V is the flow rate of the mixed gas (mL min^{-1}), m is the quality of activated sample packed in the sample column (g), t is the adsorption time for the component i (min), y_0 and y_i are the inlet and outlet gas molar flow rates, respectively. V_m = 22.4 L/mol. L mol^{-1}. The separation factor (α) of the breakthrough test is defined as follows:

$$\alpha = \frac{q_i/q_j}{y_i/y_j}$$

Where y_i and y_j represent the molar fractions of component i and j in the steel bottle, respectively.

Minor comments

a. The manuscript repeatedly uses the term "bimetallic synergistic engineering" although all

presented frameworks are monometallic Cu(II) paddlewheels. Please change to "paired Cu(II) OMS" or "dual-site Cu(II)."

Answer: We are very grateful for the reviewers' suggestions and have replaced the "bimetallic synergistic engineering" in the manuscript with "paired Cu(II) OMS synergistic".

In Revised Manuscript on Page 1.

From:

"Here, we report a dual strategy of pore topology design and bimetallic synergistic engineering to amplify chemical affinity quantum sieving (CAQS)."

To:

"Here, we report a dual strategy of pore topology design and paired Cu(II) OMS synergistic engineering to amplify chemical affinity quantum sieving (CAQS)."

In Revised Manuscript on Page 22.

From:

"In summary, we demonstrate that pore topology modulation combined with bimetallic synergistic engineering enables Cu-ATC."

To:

"In summary, we demonstrate that pore topology modulation combined with paired Cu(II) OMS synergistic engineering enables Cu-ATC."

b. The comparison plot with literature mixes data taken at different pressures (10 mbar vs 60 mbar) and slightly different temperatures without proper normalization; conclusions like

"surpassing all reported Cu-MOFs" are therefore too strong.

Answer: We sincerely appreciate the reviewer's constructive suggestions and for pointing out the errors. The exposure pressure has been uniformly set to 10 mbar in the revised manuscript, and the corresponding conclusions have been revised accordingly.

In Supporting information on Page S27.

Updated Table S1

Compound	method	T_{exp}(K)	P(mbar)	t_{exp}(min)	$S_{D2/H2}$(1:1 mixture)	Ref.
Cu-ATC	TDS	**26/40/50/60/ 77/87**	10	**10**	**7.4/18/20/13.3/9.7/ 7.4**	This work
Cu-bptc	TDS	**26/50/77/87**	10	**10**	**3.7/4.8/2/1.8**	This work
Cu-mbtc	TDS	**26/50/77**	10	**10**	**2.7/2/1.5**	This work
MOF-74-ac	TDS	20/40/**60**/**77**	10	**10**	7.1/11.4/**16.1**/**19.1**	17
MOF-74-IM-10	TDS	20/40/**60**/**77**	10	**10**	5.7/9.2/**19.6**/**25.8**	17
MOF-74-IM-38	TDS	20/40/**60**/**77**	10	**10**	8.5/12.2/**11.3**/**5.2**	17
Cu(I)-MFU-4l	TDS	90/100	10	**10**	7.1/11.1	18
CPO-27-Co	TDS	20/30/**40**/**50**/ **60**/**77**	10	**10**	1.9/2.2/2.6/**2.9**/**5.8**/ **5.5**/**5.4**	19
Ni_2Cl_2BBTA	TDS	**77/87**	10	**10**	**4.5/4**	20
Cu(I)Cu(II)-BTC	TDS	25/30/40/**50**/ **60**	10	**10**	34.4/37.9/7.4/**2.9**/**1**	21
Cu(II)-BTC	TDS	25/30/**40**/**50**/ **60**	10	**10**	4.3/**5.2**/**1.5**/**3**	21
Ag(I)-ZSM-5	TDS	40/**60**/70	10	**10**	2.4/**3.5**/9.1	22
Cu(I)-ZSM-5	TDS	**60**/100	10	**10**	**2.8**/24.9	23

In Revised Manuscript on Page 1.

From:

"achieving a D_2/H_2 selectivity of 20 at 50 K (10 mbar) and 1.8 in breakthrough experiments at 77 K, surpassing all reported Cu-MOFs."

To:

"achieving a D_2/H_2 selectivity of 20 at 50 K (10 mbar) and 1.8 in breakthrough experiments at 77 K, demonstrating excellent H_2/D_2 separation performance among Cu(II)-MOFs."

In Revised Manuscript on Page 5.

From:

"In contrast, Cu-ATC exhibited outstanding separation, achieving a D_2/H_2 selectivity of 20 at 50 K and 1.8 in breakthrough experiments at 77 K, surpassing all reported Cu(II) MOFs."

To:

"In contrast, Cu-ATC exhibited outstanding separation, achieving a D_2/H_2 selectivity of 20 at 50 K and 1.8 in breakthrough experiments at 77 K, demonstrating excellent H_2/D_2 separation performance among Cu(II)-MOFs."

c. The 87 K TDS/selectivity value in the main text does not match the value in the SI; please unify.

Answer: We sincerely appreciate the reviewer for the suggestions and for pointing out the errors. The corresponding corrections have been made in the revised manuscript.

d. Several captions (GCMC/DFT figures) do not state the exact T/P used, which makes

reproduction difficult.

Answer: We sincerely appreciate the reviewer's attention and suggestions regarding the computational details. In the GCMC simulations for the probability density distribution of H_2, the conditions were set **to** 77 K and 100 kPa. In the DFT calculations for charge density difference and adsorption energy, the conditions were set to 82 K. The revised content is presented below.

In Revised Manuscript on Page 18.

From:

"Grand Canonical Monte Carlo (GCMC) simulations were conducted in Materials Studio at 77 K and constant pressure and probability density maps of H_2 were obtained as shown in Figure 8,.

To:

"Grand Canonical Monte Carlo (GCMC) simulations were conducted in Materials Studio at 77 K, 101 kPa and constant pressure, and probability density maps of H_2 were obtained as shown in Figure 8."

e. If the authors wish to keep the strong dual-site wording, they should add at least one site-resolving dataset (VT-DRIFTS with D_2, coverage-dependent calorimetry, or deconvoluted TDS peaks with activation energies- A single gas TDS will also be helpful.).

Answer: We sincerely appreciated the reviewer's suggestion. *In situ* DRIFTS measurements for H_2/D_2 adsorption at 130 K have been added accordingly. According to the literature ***[Chem. Commun., 2023, 59, 3922–3925]***, temperature variation was reported to have a negligible effect on the red shift of the characteristic H_2/D_2 peaks.

f. It seems typo "Cu-mbtc (0.154 cm3 m-2 for D_2 and 0.172 cm3 m-2 for H_2), " □ should be

other way around.

Answer: We sincerely appreciate the reviewer for the suggestions and for pointing out the errors. The corresponding corrections have been made in the revised manuscript.

In Revised Manuscript on Page 9.

From:

"Cu-mbtc (0.154 cm^3 m^{-2} for D_2 and 0.172 cm^3 m^{-2} for H_2)"

To:

"Cu-mbtc (0.172 cm^3 m^{-2} for D_2 and 0.154 cm^3 m^{-2} for H_2)"

Reviewer #2 (Remarks to the Author):

The manuscript entitled "Dual-Orbital Synergy in Paired Cu(II) Open Metal Sites for Enhanced High-Temperature Hydrogen Isotope Separation" presents a carefully designed and well-executed study that advances the field of hydrogen isotope separation. The authors introduced a novel approach using Cu-ATC, a copper-based metal-organic framework featuring paired open metal sites. They demonstrated how dual-orbital synergy enables precise control over adsorption energetics and quantum sieving behavior.

The study employed a comprehensive suite of techniques including in situ DRIFTS, cryogenic temperature-desorption spectroscopy (TDS), column breakthrough experiments, and DFT calculations. These methods collectively provided compelling evidence for the proposed mechanism. The achieved D_2/H_2 *selectivity of 20 at 50 K represents a significant advance for Cu(II)-based MOFs. The data are internally consistent and robustly support the proposed model.*

A key strength of this work lies in its successful integration of fundamental orbital interactions with practical separation performance through systematic characterization. The authors demonstrated how spatially organized dual open metal sites enhance chemical affinity quantum sieving and thereby establish clear, evidence-based design principles. The concept of combining pore confinement with strategic open metal site positioning provides a valuable framework for rational material development that should benefit the broader research community.

This study represents an important contribution to both the mechanistic understanding of quantum sieving and the development of MOF materials capable of maintaining high selectivity at more practical operating temperatures. The work represents a valuable contribution to the field of hydrogen isotope separation. With some additional data and clarifications, this manuscript would be well-suited for publication in Nature Communications.

The following points should be addressed before acceptance:

1） The manuscript highlights the exceptional performance of Cu-ATC but does not thoroughly investigate the structural stability under multiple adsorption-desorption cycles or the framework's tolerance to potential impurities such as water and O_2. Could the authors provide data on the recyclability of Cu-ATC and discuss its chemical stability in practical operating environments?

Answer: We sincerely appreciate the reviewer's insightful comment regarding the importance of material stability and cycling performance for practical applications. In our study, the same Cu-ATC sample was used in a series of breakthrough experiments and was subsequently stored in air for six months. The XRD patterns and gas adsorption measurements obtained afterward, as shown in the figure below, indicated that the crystallinity and gas adsorption capacity of Cu-ATC remained almost unchanged, demonstrating its excellent recyclability and structural stability under these conditions. Furthermore, thermogravimetric analysis (TGA) conducted over a temperature range of 30–800 °C indicated that Cu-ATC remained structural stability below 300 °C, confirming its good thermal stability.

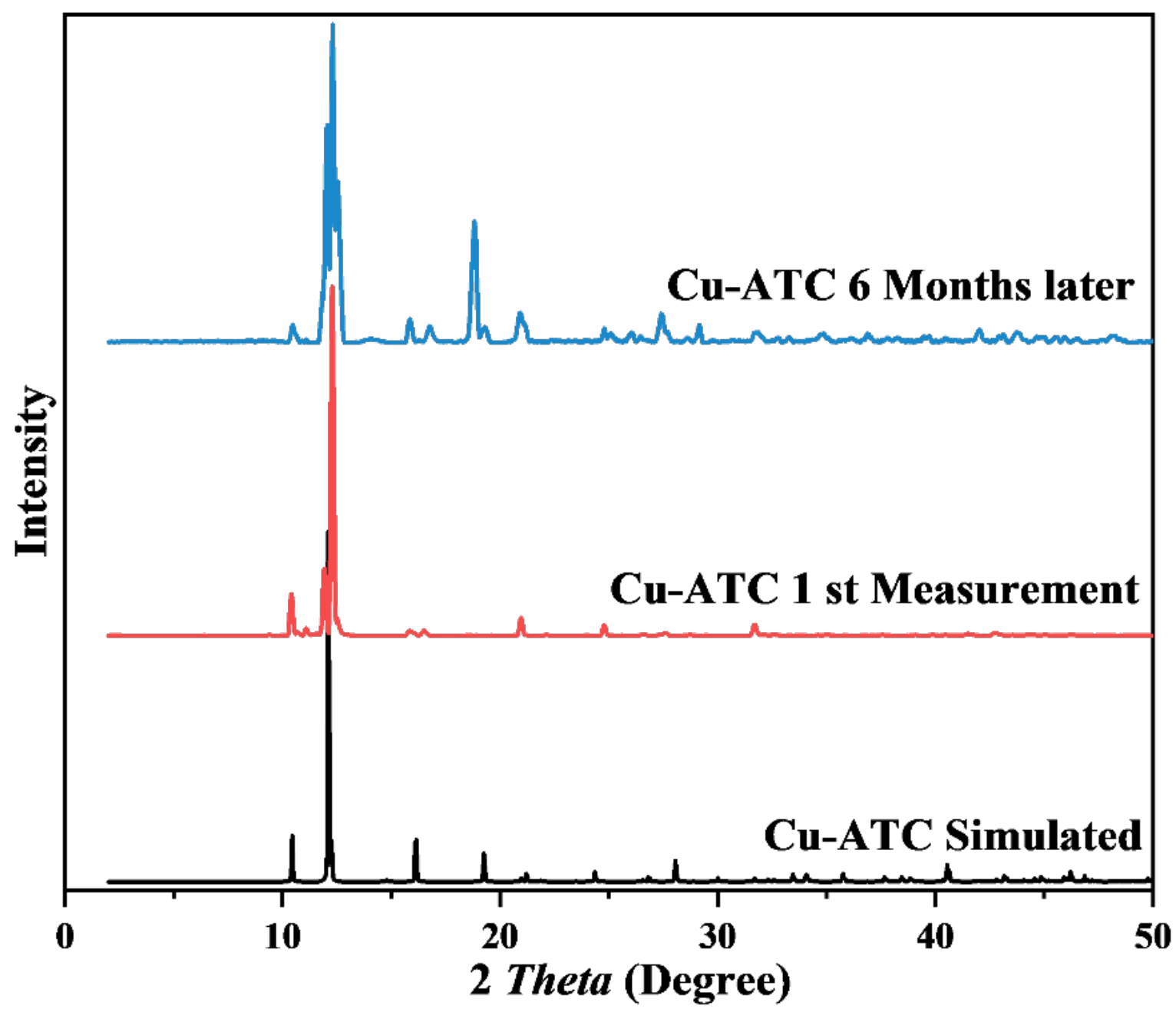

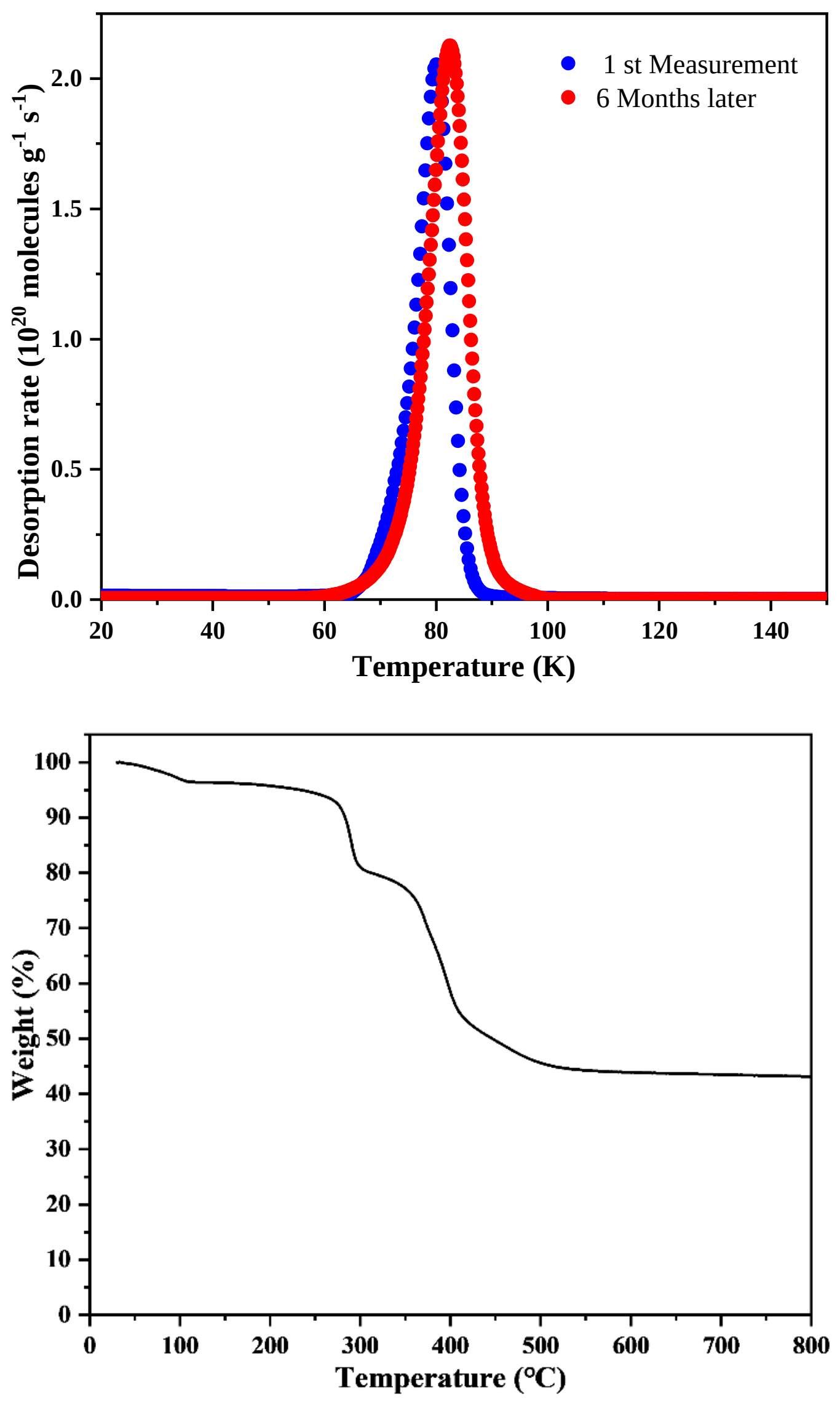

Reviewer's only figure 3.

2） The remarkable selectivity of 20 was achieved at 50 K, whereas the breakthrough experiment conducted at 77 K showed a significantly lower selectivity of 1.8. Could the authors discuss the energy penalty and technical feasibility of operating a large-scale separation unit at 50 K compared to 77 K? What is the primary factor responsible for this substantial selectivity drop over a 27 K temperature increase?

Answer: We sincerely appreciate the reviewer's comment. In fact, TDS is a static adsorption selectivity test. Under an exposure pressure of 1 kPa and an exposure time of 10 minutes, the gas molecules had sufficient time to adsorb in the pores of the material's and undergo H_2–D_2

exchange. In contrast, breakthrough experiments were conducted under dynamic conditions, in which H_2/D_2 mixture passed rapidly through the packed column with a carrier gas flow rate of 15 mL min^{-1}. As a result, the adsorption was less likely to reach equilibrium, leding to lower H_2/D_2 adsorption capacities and selectivities in the breakthrough experiments.

Nevertheless, Cu-ATC achieved a column breakthrough selectivity of 1.8 at 77 K. Since 77 K is the boiling point of liquid nitrogen, this temperature is readily accessible in industrial settings. Compared with the selectivity of approximately 1.5 achieved by current cryogenic distillation processes at 24 K, our approach not only reduced energy consumption but also provides improved selectivity. Therefore, we believe it is technically feasible.

3） The study focuses on H_2/D_2 separation. Given the more pronounced quantum effects in heavier isotopes, does the dual-OMS strategy and the enhanced CAQS effect provide an even greater advantage for separating D_2/T_2 or H_2/T_2 mixtures, which are critically important for the nuclear industry?

Answer: We sincerely appreciate the reviewer for raising this highly insightful and important question, which highlights the potential broader relevance of the core mechanism developed in this study for future applications. Our design, which enhances the chemical affinity quantum sieving (CAQS) effect through synergistic dual open metal sites (OMSs), is based on exploiting the subtle differences in zero-point energy and adsorption well depth among hydrogen isotopic molecules. The difference in zero-point energy between T_2 and D_2 is greater than that between H_2 and D_2 ***[Nature Communications 8, 14496]***. Therefore, materials suitable for H_2/D_2 separation are also hold promise for for D_2/T_2 separation.

4） In the TDS spectra of Cu-ATC at Texp = 26 K, H_2 desorption emerges anomalously earlier than D_2. This phenomenon is not observed in other materials or at other temperatures in this study. Could the authors provide a plausible explanation for this unique behavior in the main text?

Answer: We sincerely appreciate the reviewer's close attention to the details of the TDS profiles and insightful comments on this unusual feature. As generally observed, D_2 consistently desorbed earlier than H_2 as the temperature increased ***[Science 366, 613–620 (2019); Adv. Mater. 2019, 31, 1805293]***. However, for Cu-ATC at T_{exp} = 26 K(shown in figure 4a), when the temperature increased to 25~26 K, H_2 desorbed earlier than D_2. This behavior suggested that most D_2 molecules were captured by the strong adsorption sites in Cu-ATC. As a result, D_2 required a higher temperature than H_2 to be desorbed. *5） Two software packages (Materials Studio and VASP) were employed in the computational section. Could the authors elaborate on how the consistency and reliability between these computational findings were ensured?*

Answer: We sincerely appreciate the reviewer's attention and comments regarding the simulation details. To ensure consistency of the results, we re-performed all DFT calculations using the VASP software.

6） In the breakthrough experiments, Cu-mbtc exhibited a D_2/H_2 separation factor of 1.35, exceeding the value of 1.2 for Cu-bptc. This trend contradicts the observations from TDS measurements. Please provide a reasonable explanation for this apparent discrepancy.

Answer: We sincerely appreciate the reviewer's keen observation. As pointed out by the reviewer, the inconsistency in the selectivity trend of Cu-mbtc between breakthrough experiments and TDS measurements was indeed an important issue. Breakthrough experiment results are an important metric for evaluating the separation performance of materials under practical dynamic adsorption conditions. In this study, both IAST and TDS selectivities followed the order Cu-ATC > Cu-bptc > Cu-mbtc. However, in the breakthrough experiments, the order was Cu-ATC > Cu-mbtc > Cu-bptc. The main reason for this apparent discrepancy was as follows: in the breakthrough experiments, the dynamic adsorption capacities of Cu-mbtc for H_2 and D_2 (H_2: 5.5 cm^3 g^{-1}, D_2: 7.5 cm^3 g^{-1}) were much lower than those of Cu-ATC (H_2: 13.0 cm^3 g^{-1}, D_2:23.4 cm^3 g^{-1}) and Cu-bptc (H_2: 28.5 cm^3 g^{-1}, D_2: 34.2 cm^3 g^{-1}). Since the dynamic adsorption selectivity was calculated based on the dynamic adsorption capacities of H_2 and D_2, when the adsorption capacity was very low, even a small absolute difference in

adsorption capacity between D_2 and H_2 (only 2.0 cm^3 g^{-1}) was be amplified in the ratio calculation, leading to an apparently higher selectivity value.

7） It would be beneficial to include calculations of charge density difference and adsorption energy for H_2 in the VASP simulations. This would enable a direct comparative analysis with the corresponding results for D_2.

Answer: We sincerely appreciate the reviewer's attention and comments regarding the computational details. We performed calculations of charge density difference and adsorption energy for the three MOFs interacting with H_2 and D_2, respectively, using the VASP software. The revised content is as follows.

In Revised Manuscript on Page 19, 20.

From:

"Cu-ATC's primary Site I (between adjacent paddlewheels) yielded a H_2 adsorption energy of -11.01 kJ mol^{-1}, matching experimental Q_{st}, far stronger than the values at the comparable OMS in Cu-bptc (-8.52 kJ mol^{-1}) and Cu-mbtc (-7.30 kJ mol^{-1}). This enhancement arises from Cu-ATC's short Cu-Cu distance (5.98 Å)."

To:

"The calculated H_2/D_2 adsorption energies at the primary adsorption site I (between adjacent paddlewheels, shown in Figure 9a) of Cu-ATC were -9.07 kJ mol^{-1} and -13.00 kJ mol^{-1}, respectively, which are markedly stronger than those of Cu-bptc (H_2: -6.42 kJ mol^{-1}, D_2: -9.61 kJ mol^{-1}) and Cu-mbtc (H_2: -6.90 kJ mol^{-1}, D_2: -10.12 kJ mol^{-1}). To distinguish the paired-site effect from a single-site effect, we further calculated the adsorption energies for H_2/D_2 on an isolated single paddlewheel-type Cu(II) site of Cu-ATC (Figure 9b). The resulting adsorption energies (H_2: -6.16 kJ mol^{-1}, D_2: -9.62 kJ mol^{-1}) were comparable to those of Cu-bptc and Cu-mbtc. More importantly, the calculated vibrational frequencies of adsorbed H_2/D_2 in the three

MOFs (shown in Table 1) were in good agreement with the experimental values. These computational results suggest that the enhanced affinity of Cu-ATC for hydrogen isotopes cannot be attributed to an individual Cu site alone, but is instead associated with the closely spaced Cu···Cu arrangement (5.98 Å). Consistent with this interpretation, the charge density difference maps (Figure 10) reveal electron redistribution involving the adsorbed hydrogen isotope molecule and the two closely spaced Cu open metal sites in Cu-ATC, while the corresponding single-site model as well as Cu-bptc and Cu-mbtc do not exhibit the same dual-site interaction feature. Taken together, these results support the conclusion that the enhanced adsorption strength of Cu-ATC arises from a cooperative effect associated with the paired Cu OMSs."

In Revised Manuscript on Page 20, 21.

Added Figure 9

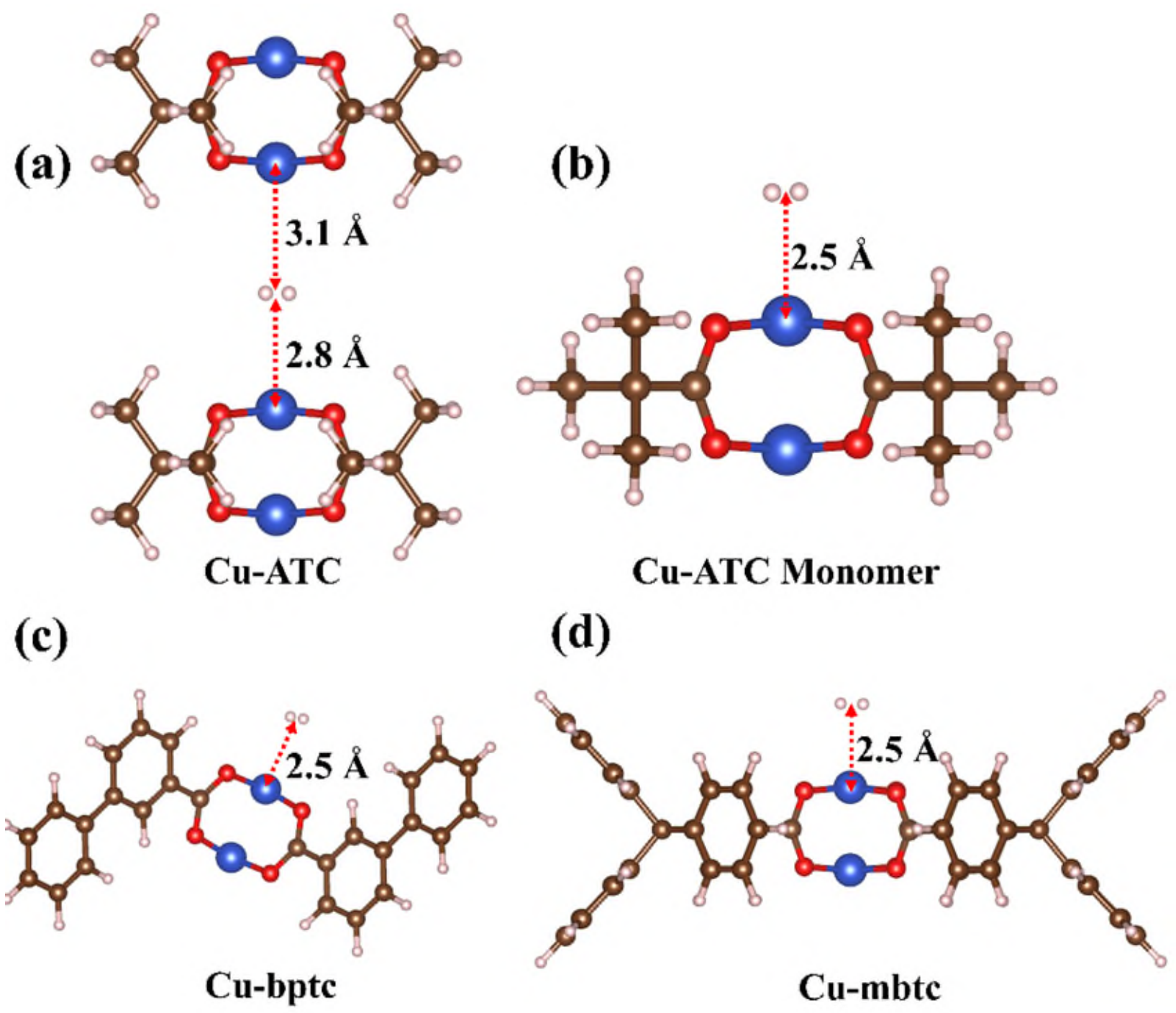

Figure 9. Structure of finite model of (a) Cu-ATC, Cu-ATC Monomer(c) Cu-bptc, and (d) Cu-mbtc as optimized by DFT and corresponding equilibrium position of the hydrogen molecule adsorbed at the Cu(II) atom. (blue, copper; red, oxygen; tan, carbon; pink, hydrogen)

Updated Figure 10

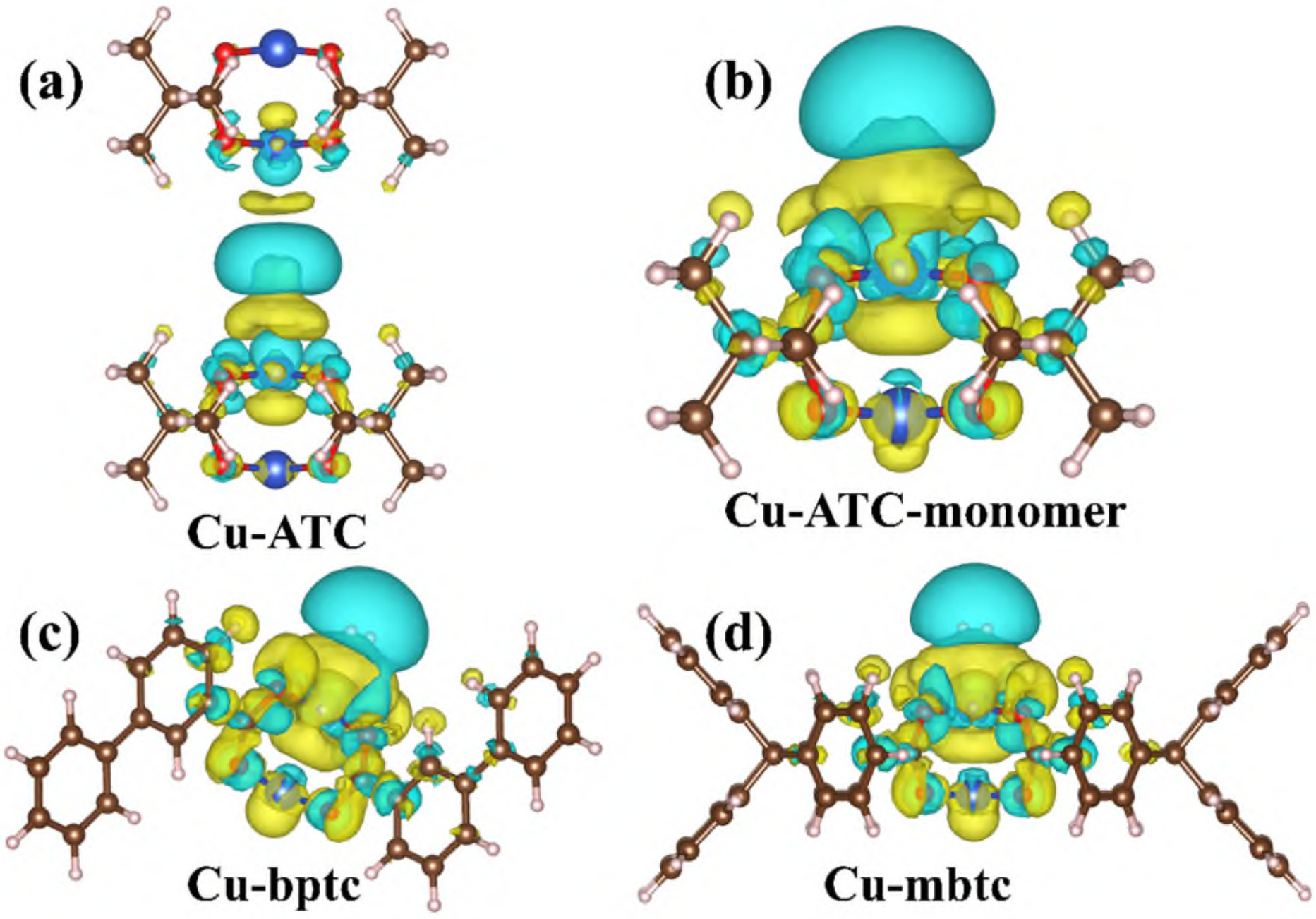

Figure 10. The 3D charge density difference of (a) between the Cu OMSs of adjacent paddlewheels in Cu-ATC and about a single Cu OMS in Cu-ATC-monomer (b), Cu-bptc (c),Cu-mbtc (d) as determined from simulation. The yellow zone and cyan zone denote the charge accumulation and depletion.

8） The authors should provide the purity information for all test gases used in the experiments.

Answer: We sincerely appreciate the reviewer's suggestion. The purities of the gases used in all measurements were as follows: H_2 (99.999%), D_2 (99.999%), N_2 (99.999%), and Ar (99.999%). This information has been supplemented in the Supporting Information.

9） Some physical quantities should be written in italics.

Answer: We sincerely appreciated the reviewer's suggestions and for pointing out the errors. The corresponding corrections were made in the revised manuscript.

10) Reference format should be checked and corrected.

Answer: We sincerely appreciated the reviewer's suggestions and for pointing out the errors. The corresponding corrections were made in the revised manuscript.

Reviewer #3 (Remarks to the Author):

The manuscript investigates hydrogen-isotope separation using Cu(II) paddlewheel MOFs and advances a clear design principle in which facing paired open-metal sites are embedded within ultramicroporous channels to enhance chemical-affinity quantum sieving. The concept is novel, as dual opposing OMSs create a confined interaction environment that strengthens CAQS through cooperative orbital engagement while preserving diffusion pathways. The methodology is tightly integrated, combining two closely related structural controls with TDS, column breakthrough, in situ DRIFTS, and GCMC/DFT to build a coherent structure-to-mechanism-to-performance narrative that connects macroscopic metrics with molecular-level insight. Especially, for Cu-ATC, DFT calculations confirmed the strong interaction between the σ orbital of hydrogen isotope molecules and both Cu dz2 orbital, which underscores both fundamental significance and application relevance. Therefore, Cu-ATC has meaningful selectivity and favorable kinetics persist near liquid-nitrogen temperature. I recommend publication in Nature Communications after minor revision.

1. At line 42, the authors mention utilizing quantum effects for hydrogen isotope separation but provide limited mechanistic detail. A more substantive discussion of the underlying physical principles governing these quantum effects would strengthen the introductory narrative and better contextualize the experimental strategy.

Answer: We sincerely appreciate the reviewer's suggestion and fully agree that a more in-depth elucidation of the physical principles underlying quantum effects in the Introduction would strengthen the foundation of the research. Accordingly, the following content has been added to the revised manuscript.

In Revised Manuscript on Page 3

From:

"The intrinsic difficulty in hydrogen isotope separation lies in their nearly identical physicochemical properties, with the primary difference being a mass ratio of two, necessitating the exploitation of quantum effects for efficient separation under mild conditions."

To:

"The intrinsic difficulty in hydrogen isotope separation lies in their nearly identical physicochemical properties, with the primary difference being a twofold mass difference. The heavier isotope (D_2) has a shorter de Broglie wavelength and a lower zero-point energy, making it more readily adsorbed by microporous materials. This quantum effect can therefore be leveraged for effective isotope separation."

2. The Cu(I)-exchanged ZSM-5 system cited in line 54, which does not exhibit KQS–CAQS synergy, would be more appropriately repositioned within the section introducing CAQS effects, as this placement would enhance the logical flow of the mechanistic discussion.

Answer: We sincerely appreciate the reviewer's suggestion and fully agree with the reviewer's perspective. Accordingly, the relevant discussion has been repositioned in the revised manuscript to improve the logical flow of the mechanistic discussion.

In Revised Manuscript on Page 4, 5.

From:

"Building on this, researchers have attempted to combine the two mechanisms. Imidazole-modified MOF-74 achieved a selectivity of 26 at 77 K, [18] while Cu(I)-exchanged ZSM-5 reached 24.9 at 100 K. [19] These studies illustrate the potential of KQS–CAQS synergy, yet they also highlight persistent challenges including the instability of Cu(I) sites, the complexity of

post-synthetic modification, and the rapid loss of selectivity once the temperature rises above 77 K.”

To:

“Building on this, researchers have further pursued new explorations based on the CAQS separation mechanism. Imidazole-modified MOF-74 attained a selectivity of 26 at 77 K, demonstrating the potential of KQS-CAQS synergy. In addition, Cu(I)-exchanged ZSM-5 reached a selectivity of 24.9 at 100 K, illustrating the feasibility of introducing open metal sites (OMSs) in porous materials. However, these studies also revealed several shortcomings, including the instability of Cu(I) sites, the complexity of post-synthetic modification, and the rapid loss of selectivity at temperature rises above 77 K.”

3. To facilitate reader comprehension of the structural hierarchy, the authors should indicate the spherical diameter representing the intracage space in Figures 1e and 1f.

Answer: We sincerely appreciate the reviewer’s suggestion. We agree that improving the caption information would help readers better understand the crystal structures of the MOFs. Accordingly, this information has been added to the revised manuscript.

In Revised Manuscript on Page 6

From:

“(Atom colors: C: gray, O: red, Cu: cyan, H: white, and the yellow sphere represents the largest sphere that would occupy the cavity without contacting the interior van der Waals surface)”

To:

“(Atom colors: C: gray, O: red, Cu: cyan, H: white, and the yellow sphere represents the largest sphere that would occupy the cavity without contacting the interior van der Waals surface with diameters of 8 Å and 10 Å, respectively)”

4. In the "Adsorption Mechanism Study" section, while charge density difference and electron density distribution were calculated for Cu-ATC, the authors should incorporate corresponding computations for Cu-bptc and Cu-mbtc. This addition would establish a parallel and more rigorous comparative framework across the three MOF systems.

Answer: We sincerely appreciate the reviewer's comment and fully agree with the reviewer's perspective. In response, we have expanded the computational analysis to include Cu-bptc and Cu-mbtc**,** thereby establishing a more parallel comparative framework across the three MOF systems. The updated computational results are presented below.

In Revised Manuscript on Page 19, 20.

From:

"Cu-ATC's primary Site I (between adjacent paddlewheels) yielded a H_2 adsorption energy of -11.01 kJ mol^{-1}, matching experimental Q_{st}, far stronger than the values at the comparable OMS in Cu-bptc (-8.52 kJ mol^{-1}) and Cu-mbtc (-7.30 kJ mol^{-1}). This enhancement arises from Cu-ATC's short Cu-Cu distance (5.98 Å)."

To:

"The calculated H_2/D_2 adsorption energies at the primary adsorption site I (between adjacent paddlewheels, shown in Figure 9a) of Cu-ATC were -9.07 kJ mol^{-1} and -13.00 kJ mol^{-1}, respectively, which are markedly stronger than those of Cu-bptc (H_2: -6.42 kJ mol^{-1}, D_2: -9.61 kJ mol^{-1}) and Cu-mbtc (H_2: -6.90 kJ mol^{-1}, D_2: -10.12 kJ mol^{-1}). To distinguish the paired-site effect from a single-site effect, we further calculated the adsorption energies for H_2/D_2 on an isolated single paddlewheel-type Cu(II) site of Cu-ATC (Figure 9b). The resulting adsorption energies (H_2: -6.16 kJ mol^{-1}, D_2: -9.62 kJ mol^{-1}) were comparable to those of Cu-bptc and Cu-mbtc. More importantly, the calculated vibrational frequencies of adsorbed H_2/D_2 in the three MOFs (shown in Table 1) were in good agreement with the experimental values. These

computational results suggest that the enhanced affinity of Cu-ATC for hydrogen isotopes cannot be attributed to an individual Cu site alone, but is instead associated with the closely spaced Cu···Cu arrangement (5.98 Å). Consistent with this interpretation, the charge density difference maps (Figure 10) reveal electron redistribution involving the adsorbed hydrogen isotope molecule and the two closely spaced Cu open metal sites in Cu-ATC, while the corresponding single-site model as well as Cu-bptc and Cu-mbtc do not exhibit the same dual-site interaction feature. Taken together, these results support the conclusion that the enhanced adsorption strength of Cu-ATC arises from a cooperative effect associated with the paired Cu OMSs."

In Revised Manuscript on Page 20, 21.

Added Figure 9

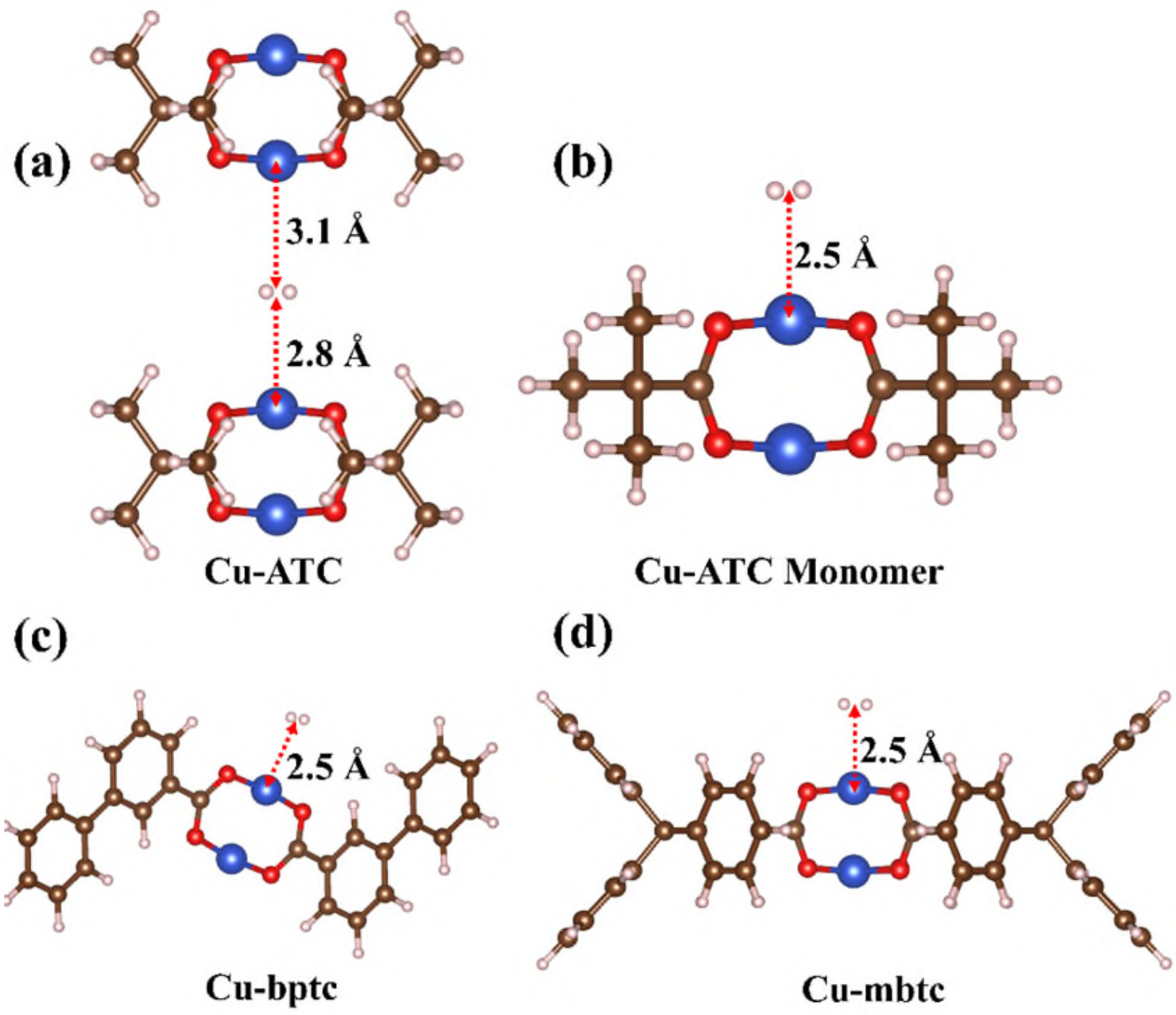

Figure 9. Structure of finite model of (a) Cu-ATC, Cu-ATC Monomer(c) Cu-bptc, and (d) Cu-mbtc as optimized by DFT and corresponding equilibrium position of the hydrogen molecule adsorbed at the Cu(II) atom. (blue, copper; red, oxygen; tan, carbon; pink, hydrogen)

Updated Figure 10

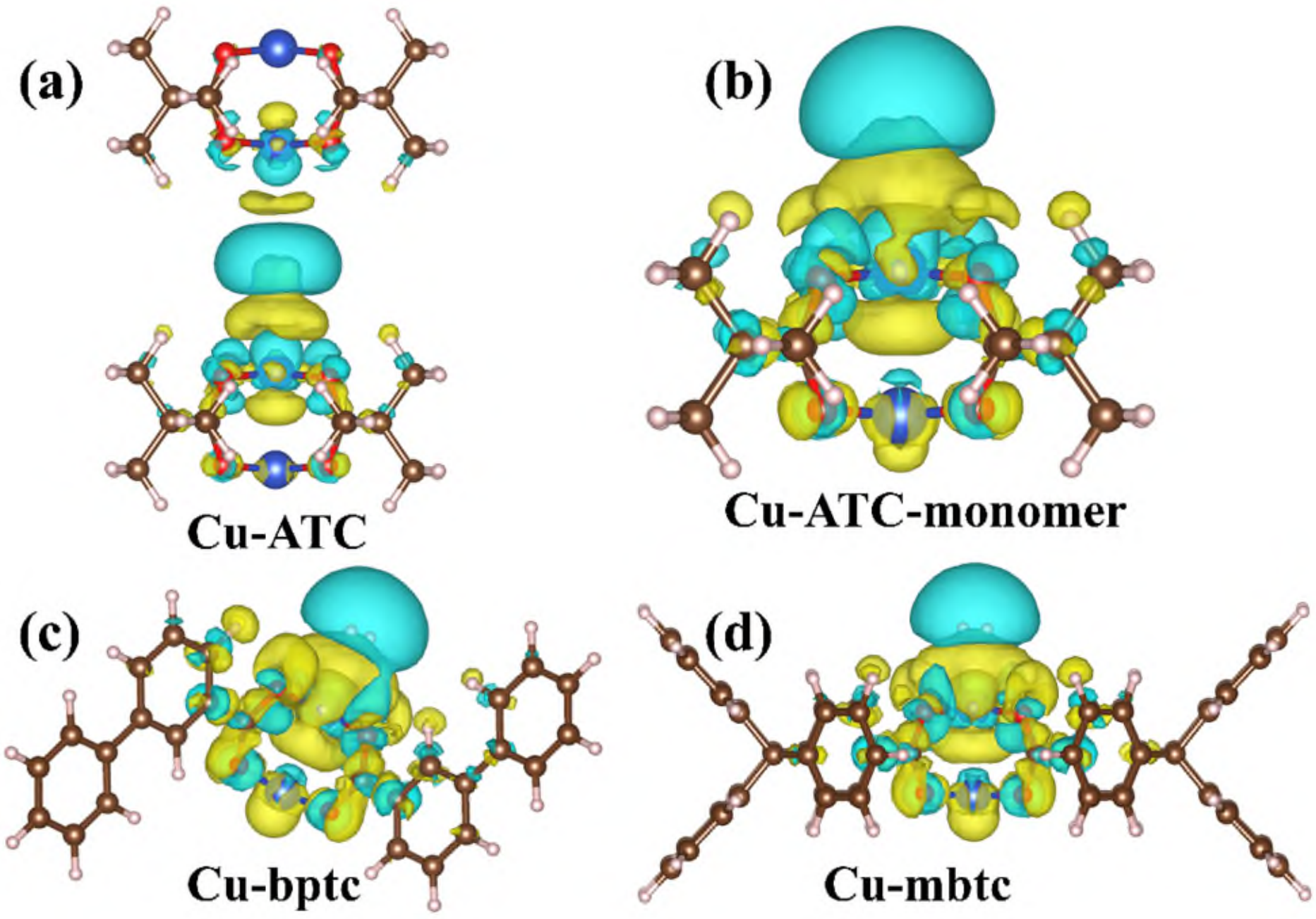

Figure 10. The 3D charge density difference of (a) between the Cu OMSs of adjacent paddlewheels in Cu-ATC and about a single Cu OMS in Cu-ATC-monomer (b), Cu-bptc (c),Cu-mbtc (d) as determined from simulation. The yellow zone and cyan zone denote the charge accumulation and depletion.

5. In the "Adsorption properties of H2 and D2" section, while the authors present adsorption capacities normalized by specific surface area, a comparison based on adsorption capacity per open metal site would offer more direct mechanistic insight, given that OMS are the primary active centers governing isotope selectivity in this work.

Answer: We sincerely appreciate the reviewer's suggestion. After a detailed analysis of the adsorption behavior of hydrogen isotopes within the MOF channels, we found that hydrogen isotopes were not necessarily adsorbed exclusively at the open metal sites; they could also be adsorbed at weak adsorption sites such as oxygen atoms. Therefore, calculating the number of H_2/D_2 molecules adsorbed per open metal site would not be available. Moreover, we noted that due to the size differences between hydrogen isotope molecules and nitrogen or argon molecules, the accessible micropores and surface areas varied for each gas, making the

calculation of H_2/D_2 uptake normalized by specific surface area imprecise. Accordingly, we have removed this description from the revised manuscript.

6. In the analysis of the TDS spectra for Cu-bptc (Figures 4c, d), the first desorption peak is assigned to ultramicropores and the second to oxygen atoms. The authors should provide explicit justification for this assignment to ensure interpretive rigor. Possible supporting evidence could include computational energetics, site occupancy analysis, or previous literatures.

Answer: We sincerely appreciate the reviewer's comment and fully agree with the reviewer's perspective. Based on the references ***[Science 366, 613–620 (2019); J. Am. Chem. Soc. 2017, 139, 15135−15141; Adv. Mater. 2019, 31, 1805293]***, together with the experimental Q_{st} (Figure 2c), *In situ* DRIFTS measurements at 130 K (Figure 3), DFT-calculated adsorption energies (Figure 9), H_2/D_2 vibrational frequencies (Table 1), and charge density difference maps (Figure 10), we made the following inference: (1) the adsorption energy of hydrogen isotope molecules at OMSs was the strongest, and therefore the desorption temperature required was the highest, corresponding to the TDS desorption peaks appearing at the highest temperature range (80–100 K); (2) oxygen atoms in the MOF framework also exhibited a certain adsorption affinity for hydrogen isotope molecules, albeit weaker than that of open metal sites, resulting in a desorption temperature slightly lower than that of open metal sites (60–70 K); (3) if benzene rings were present in the MOF structure, they exhibited weak adsorption toward hydrogen isotope molecules, corresponding to the lowest desorption temperature (30–50 K); and (4) in the presence of ultramicropores, the kinetic quantum sieving (KQS) effect enabled hydrogen isotope adsorption even at low temperatures, and the corresponding TDS desorption peaks might overlap with those associated with oxygen atoms or benzene rings (30–50 K).

7. The TDS selectivity of both Cu-ATC and Cu-bptc shows a characteristic pattern of initial increase followed by decrease with rising exposure temperature. The authors should elucidate the underlying mechanism responsible for this behavior, as understanding this trend is

important for optimizing operating conditions and clarifying the balance between kinetic and thermodynamic factors.

Answer: We sincerely appreciated the reviewer's insightful comment and fully agreed with the reviewer's perspective. The variation trend of TDS selectivity effectively reflected the adsorption behavior of hydrogen isotopes on the material and helped us establish a more comprehensive and accurate structure–performance relationship. Therefore, we supplemented the TDS selectivity measurements at 40 K and 60 K to better observe the trend of selectivity variation. Additionally, we would like to note that since TDS selectivity was influenced by the combined effects of multiple factors, an optimal operating temperature may exist, as reported in the literature [***J. Am. Chem. Soc. 2017, 139, 15135−15141; J. Mater. Chem. A, 2020, 8, 6319–6327***]. At very low temperatures (26 K, 40 K), the pore size of Cu-ATC was comparable to the de Broglie wavelengths of H_2 and D_2, and the kinetic quantum sieving (KQS) effect dominated. Under these conditions, weak adsorption sites within the pores contributed to the uptake of both isotopes, resulting in relatively low selectivity. As the temperature increased from 26 K to 50 K, the thermal motion of H_2 and D_2 molecules intensified and their de Broglie wavelengths shortened, diminishing the influence of the KQS effect. Concurrently, the chemically affinity quantum sieving (CAQS) effect arising from open metal sites became predominant, amplifying the difference in adsorption enthalpy between H_2 and D_2. Consequently, the selectivity reached its optimal value at 50 K. When the temperature exceeded 50 K, molecular thermal motion became even more vigorous, weakening the adsorption at open metal sites and leading to a decrease in selectivity. Overall, the TDS selectivity exhibited a characteristic pattern of initially increasing and then decreasing with rising exposure temperature.

Reviewer #4 (Remarks to the Author):

The manuscript on "Dual-Orbital Synergy in Paired Cu(II) Open Metal Sites for Enhanced High-Temperature Hydrogen Isotope Separation" by Fenglei Wang et al. reports on gas adsorption isotherms, DRIFT spectroscopy, TDS measurements, breakthrough measurements and GCMC calculations for H2/D2 isotope separation. Several experimental details are missing, the interpretation and assignment of adsorption sites is not clear and not well supported by the experimental data.

1. Structure characterization: The BET area is analyzed measuring nitrogen adsorption isotherms. The IUPAC report DOI 10.1515/pac-2014-1117 suggest that preferentially Argon should be used, but nitrogen might be okay but with a higher uncertainty. However, the BET method is not valid for microporous materials, but can be applied using special criteria (so-called Rouquerol's criteria), see IUPAC report. The authors don't specify which pressure range has been applied and if the criteria are met. This should be checked and included in the SI.

Answer: We sincerely thank the reviewer for this valuable comment. We agree that, for microporous materials, N_2 adsorption at 77 K may introduce additional uncertainty because of diffusion and quadrupole-related effects. We additionally performed Argon adsorption–desorption measurements at 87 K to obtain more reliable surface area values. As shown in the figure below, the BET surface areas derived from Ar adsorption–desorption at 87 K for Cu-ATC (S_{BET} = 572 m^2 g^{-1}), Cu-bptc (S_{BET} = 1664 m^2 g^{-1}), and Cu-mbtc (S_{BET} = 439 m^2 g^{-1}) were similar to those of Cu-ATC (S_{BET} = 606 m^2 g^{-1}), Cu-bptc (S_{BET} = 1811 m^2 g^{-1}), and Cu-mbtc (S_{BET} = 498 m^2 g^{-1}) derived from N_2 adsorption–desorption at 77 K with a pressure range of 10^{-6}~1 p/p^0. The BET analysis was performed using the Micromeritics software package in accordance with the Rouquerol's criteria, and this information was noted in the Supporting Information.

In Supporting information on page S14, S15, S16.

Added figure S7, S8, S9.

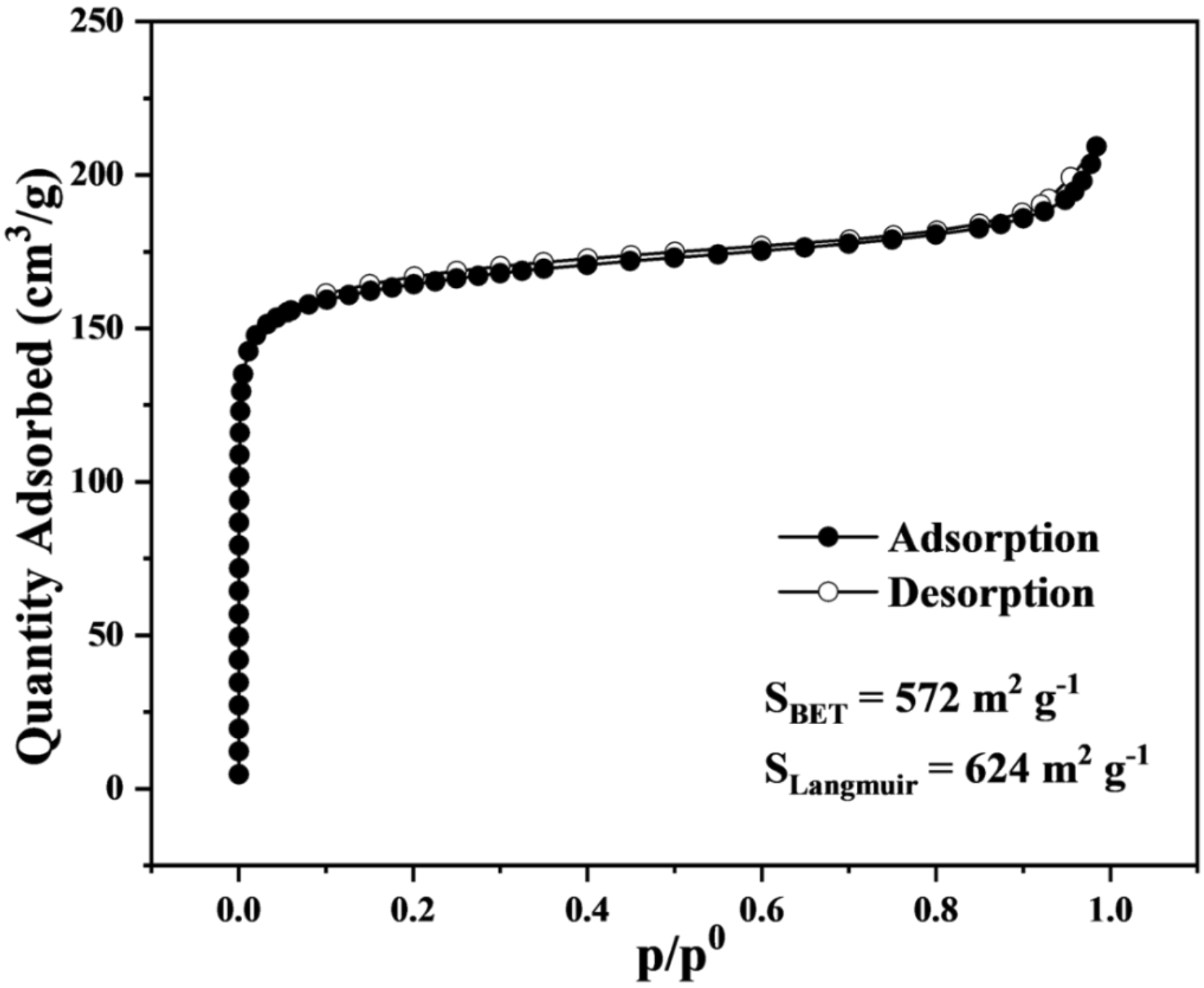

Ar isotherm for Cu-ATC at 87 K.

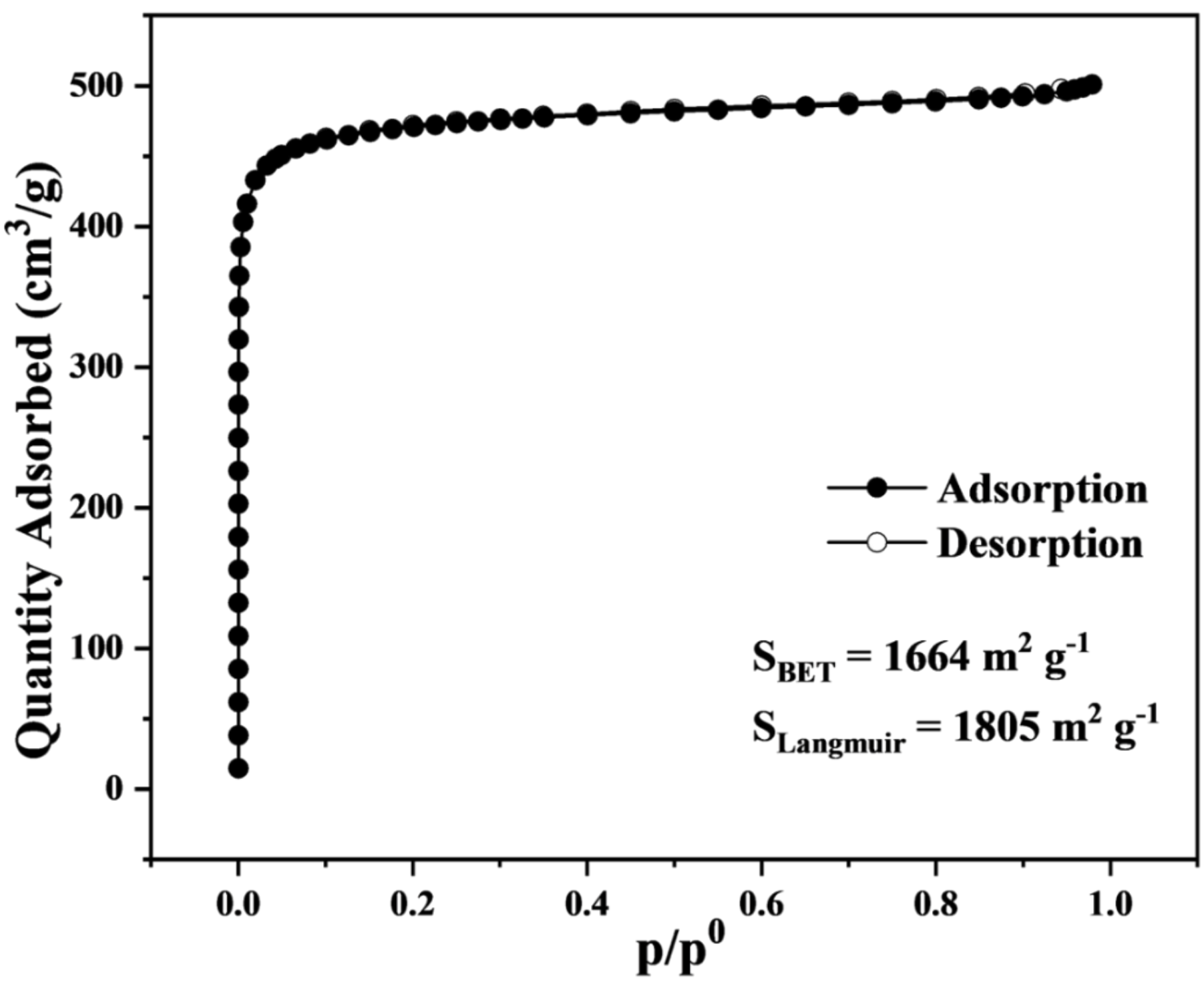

Ar isotherm for Cu-bptc at 87 K.

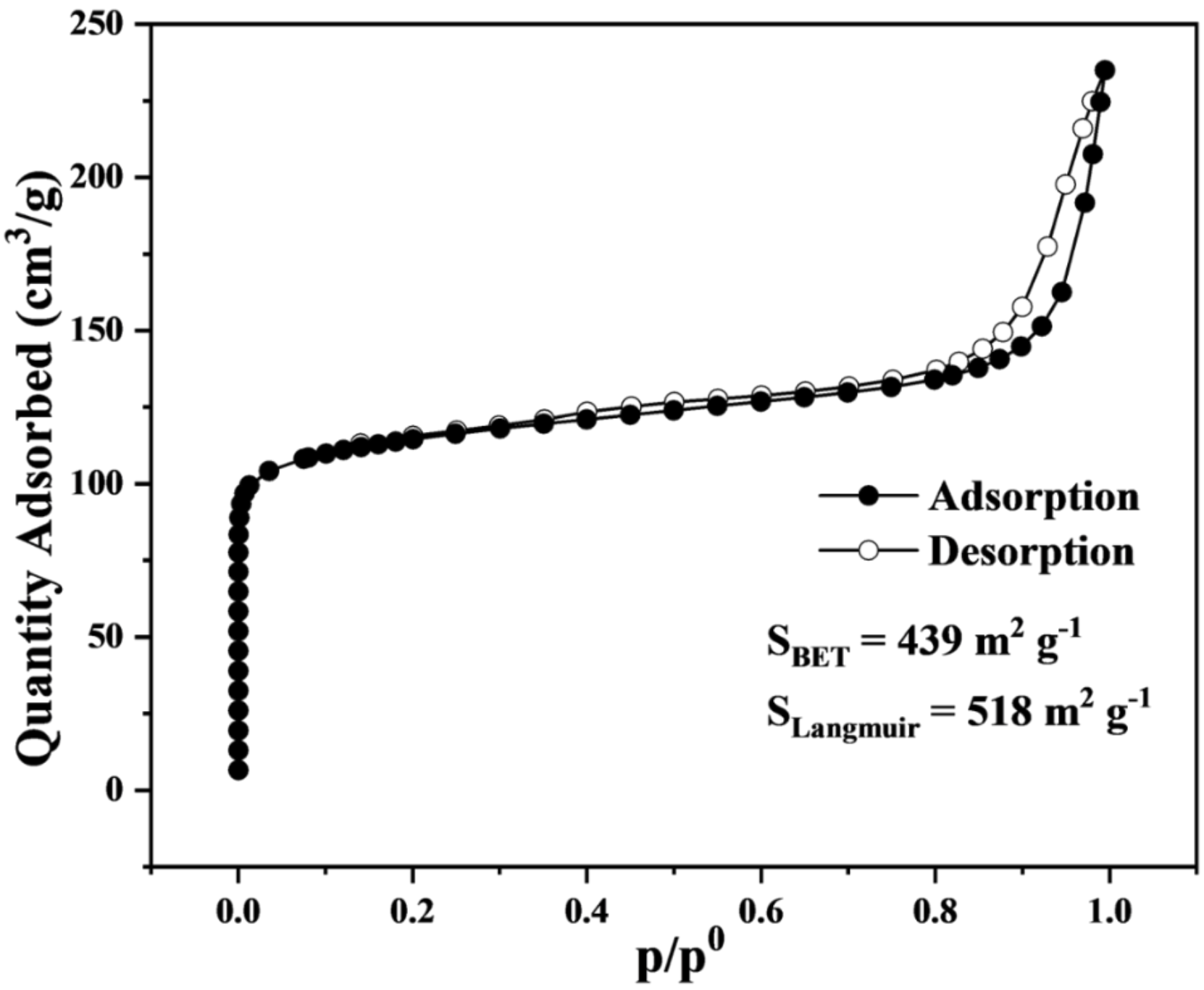

Ar isotherm for Cu-mbtc at 87 K.

2. Adsorption isotherms of H2 and D2: The uptake values are given in a ridiculous accuracy "high uptake capacities (q): 248.907 cm3 g-1 for D_2 and 219.596 cm3 g-1 for H_2". Then the authors discuss the H2 and D2 adsorption by comparing to the BET area obtained by N2. The different size of the molecules may easily cause different areas and pores accessible for each molecule, therefore, such a comparison is questionable. They mention "despite a comparable surface area to Cu-ATC, highlighting the adverse effect of channel expansion" but no proof for an expansion is given. In Figure 2(c) the adsorbed uptake is divided by the N2 BET area, which might be a quite random approach considering my previous arguments.

Answer: We sincerely thank the reviewer for this valuable comment. In the revised manuscript, these values have been corrected. We also agree with the reviewer that normalizing H_2/D_2 uptake by the BET surface area derived from N_2 adsorption is not an appropriate basis for comparison. Accordingly, we have removed the discussion and figure presentation related to H_2/D_2 uptake normalized by the N_2 BET surface area from the revised manuscript.

Finally, we appreciate the reviewer for pointing out that the term "channel expansion" was imprecise. Our intended meaning was not that the framework undergoes a structural expansion,

but rather that Cu-mbtc possesses wider one-dimensional channels (~10 Å) than Cu-ATC. Thus, although the two frameworks share similar topological features, the wider channels in Cu-mbtc provide a less favorable confinement environment for hydrogen isotope adsorption. Therefore, in the revised manuscript, the term "channel expansion" was replaced with "larger channel size."

The revised content is as follows.

In Revised Manuscript on Page 8.

From:

"At 77 K and 101 kPa, Cu-bptc exhibited high uptake capacities (q): 248.907 cm^3 g^{-1} for D_2 and 219.596 cm^3 g^{-1} for H_2, outperforming many reported MOF-based hydrogen storage materials. In contrast, Cu-ATC showed lower capacities (154.2 cm^3 g^{-1} for D_2 and 146.6 cm^3 g^{-1} for H_2)"

To:

"At 77 K and 101 kPa, Cu-bptc exhibited high uptake capacities (q): 249 cm^3 g^{-1} for D_2 and 220 cm^3 g^{-1} for H_2. In contrast, Cu-ATC showed lower capacities of 154 cm^3 g^{-1} for D_2 and 147 cm^3 g^{-1} for H_2."

In Revised Manuscript at Page 9.

From:

"Cu-mbtc displayed even lower uptakes (85.6 cm^3 g^{-1} for D_2 and 76.8 cm^3 g^{-1} for H_2), despite a comparable surface area to Cu-ATC, highlighting the adverse effect of channel expansion (~10 Å) in topologically similar frameworks."

To:

“Cu-mbtc displayed even lower uptakes (86 cm^3 g^{-1} for D_2 and 77 cm^3 g^{-1} for H_2), this result was attributed to its larger channel size (~10 Å) relative to Cu-ATC, which provided a less favorable confinement environment for hydrogen adsorption in these topologically similar frameworks.

In Revised Manuscript at Page 13.

From:

attributed to channel expansion (~10 Å)

To:

attributed to its larger channel size (~10 Å)

3. TDS measurements: The authors claim in the first sentence: “To identify adsorption sites in the three MOFs and their affinity for H2/D2, TDS measurements were conducted.” TDS is a macroscopic measurement of the desorption rate during a constant heating in high vacuum, therefore, no assignment to a microscopic adsorption site is possible. Considering this, the assignment of the specific sites to different TDS maxima is done only by intuition and could be as well completely different or wrong. In Figure 4(a) the site I is even proposed to be occupied on opposite sides in the channel, a highly unlikely situation at relatively high temperature.

Answer: We sincerely appreciate the reviewer’s comment. We agree that TDS is a macroscopic desorption measurement and, by itself, does not provide direct microscopic identification of specific adsorption sites. In the revised version, the TDS features are interpreted more cautiously as reflecting different adsorption environments rather than being treated as direct proof of specific microscopic adsorption sites. Based on prior literature about hydrogen isotope adsorption in porous materials [***Science 366, 613–620 (2019); J. Am. Chem. Soc. 2017, 139, 15135−15141; Adv. Mater. 2019, 31, 1805293***]. We made the following inference based on the experimental Q_{st} (Figure 2c), *In situ* DRIFTS measurements at 130 K (Figure 3), DFT-

calculated adsorption energies (Figure 9), H_2/D_2 vibrational frequencies (Table 1), and charge density difference maps (Figure 10):: (1) the adsorption energy of hydrogen isotope molecules at OMSs was the strongest, and therefore the desorption temperature required was the highest, corresponding to the TDS desorption peaks appearing at the highest temperature range (80–100 K); (2) oxygen atoms in the MOF framework also exhibited a certain adsorption affinity for hydrogen isotope molecules, albeit weaker than that of open metal sites, resulting in a desorption temperature slightly lower than that of open metal sites (60–70 K); (3) if benzene rings were present in the MOF structure, they exhibited weak adsorption toward hydrogen isotope molecules, corresponding to the lowest desorption temperature (30–50 K); and (4) in the presence of ultramicropores, the kinetic quantum sieving (KQS) effect enabled hydrogen isotope adsorption even at low temperatures, and the corresponding TDS desorption peaks might overlap with those associated with oxygen atoms or benzene rings (30–50 K).

We also agreed that the specific occupancy pattern illustrated in the original Figure 4(a), particularly the assignment of site I to simultaneous interactions with both Cu sites, lacked direct evidence. Therefore, we made the following inference based on the experimental Q_{st}, *In situ* DRIFTS measurements at 130 K (Figure 3), and DFT-calculated adsorption energies (Figure 9), H_2/D_2 vibrational frequencies (Table 1), and charge density difference maps (Figure 10): in Cu-ATC, the paired Cu sites were in close proximity (5.98 Å), so H_2/D_2 molecules were likely able to interact simultaneously with paired Cu OMSs, resulting in strong interactions.

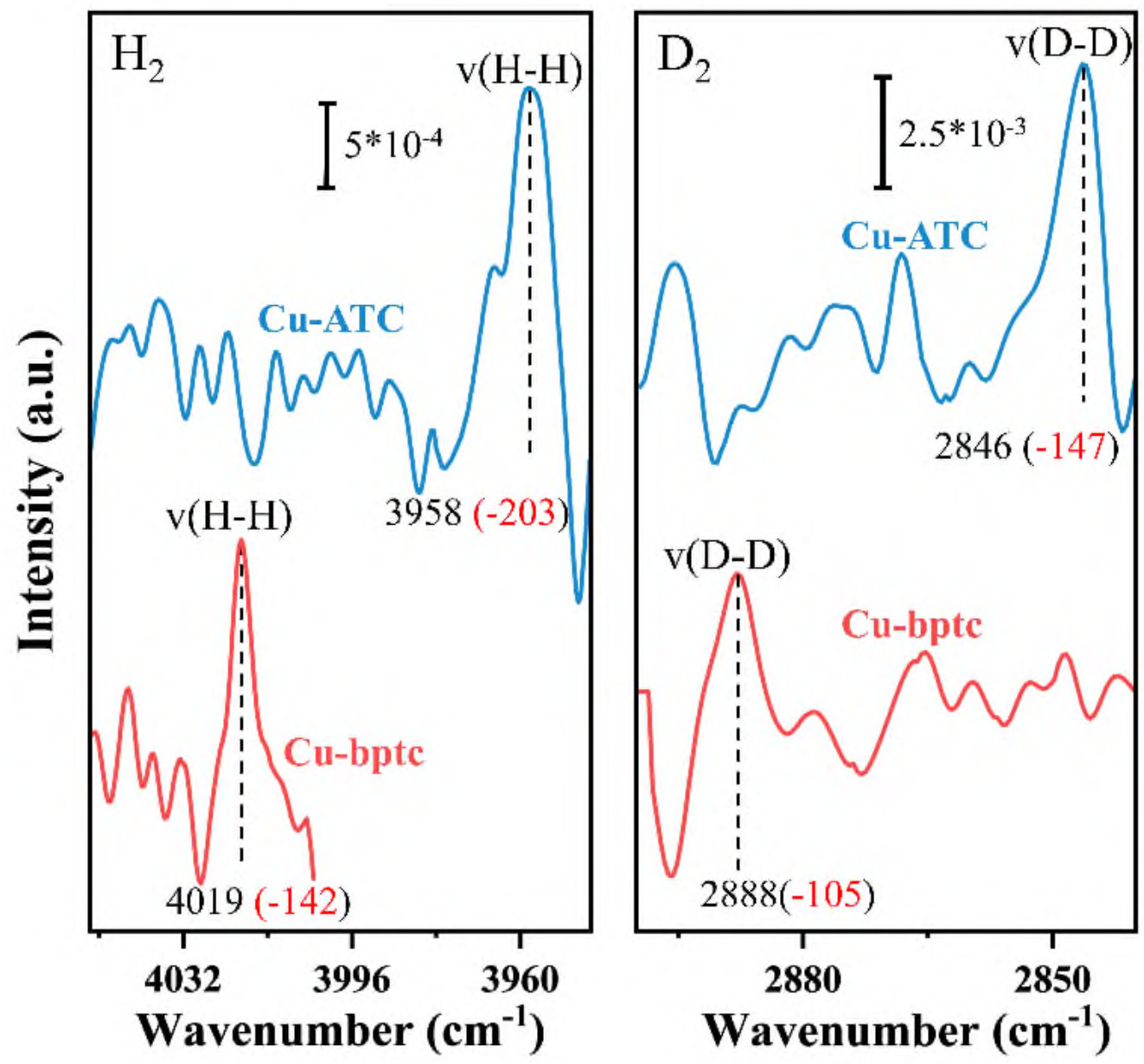

Figure 3. *In situ* DRIFT spectra of of adsorbed H_2 and D_2 in the activated Cu-ATC (blue)/Cu-bptc (red). Gas was loaded at 130 K/1 atm.

Structures	H_2	H_2 red shift	D_2	D_2 red shift
Cu-ATC	3931 (3958) cm^{-1}	230 (203) cm^{-1}	2830 (2846) cm^{-1}	163 (147) cm^{-1}
Cu-bptc	4011 (4019) cm^{-1}	150 (142) cm^{-1}	2886(2888) cm^{-1}	107 (105) cm^{-1}
Cu-ATC monomer	4011 cm^{-1}	152 cm^{-1}	2884 cm^{-1}	109 cm^{-1}

Table 1. The DFT-computed vibrational frequencies of H_2 and D_2 adsorbed on Cu-ATC, Cu-bptc, Cu-mbtc, and the Cu-ATC monomer, with experimental values given in parentheses.

4.Figure 5 shows the dependence on exposure temperature, this should be clearly indicated on the x-axis. It is not described how the experiment is performed, is the evacuation of the non-adsorbed gas performed at the exposure temperature? Furthermore, the amount of D2 desorbed should be included either in Figure 5 or S10-12. For separation efficiency one should consider both selectivity and uptake.

Answer: We sincerely appreciate the reviewer's comments regarding the TDS adsorption selectivity measured at different exposure temperatures. Accordingly, we have changed the

x-axis label in Figure 5 to clearly indicate the exposure temperature (T_{exp}).

We also updated the details of the TDS measurement procedure in the Supporting Information Section 2, specifying that non-adsorbed H_2/D_2 gases were evacuated using a molecular pump at the exposure temperature prior to the TDS measurement. Additionally, to provide a more complete evaluation of the separation performance, we have added the D_2 adsorption capacities measured at different exposure temperatures, as shown in the figure below.

Updated Figure 5.

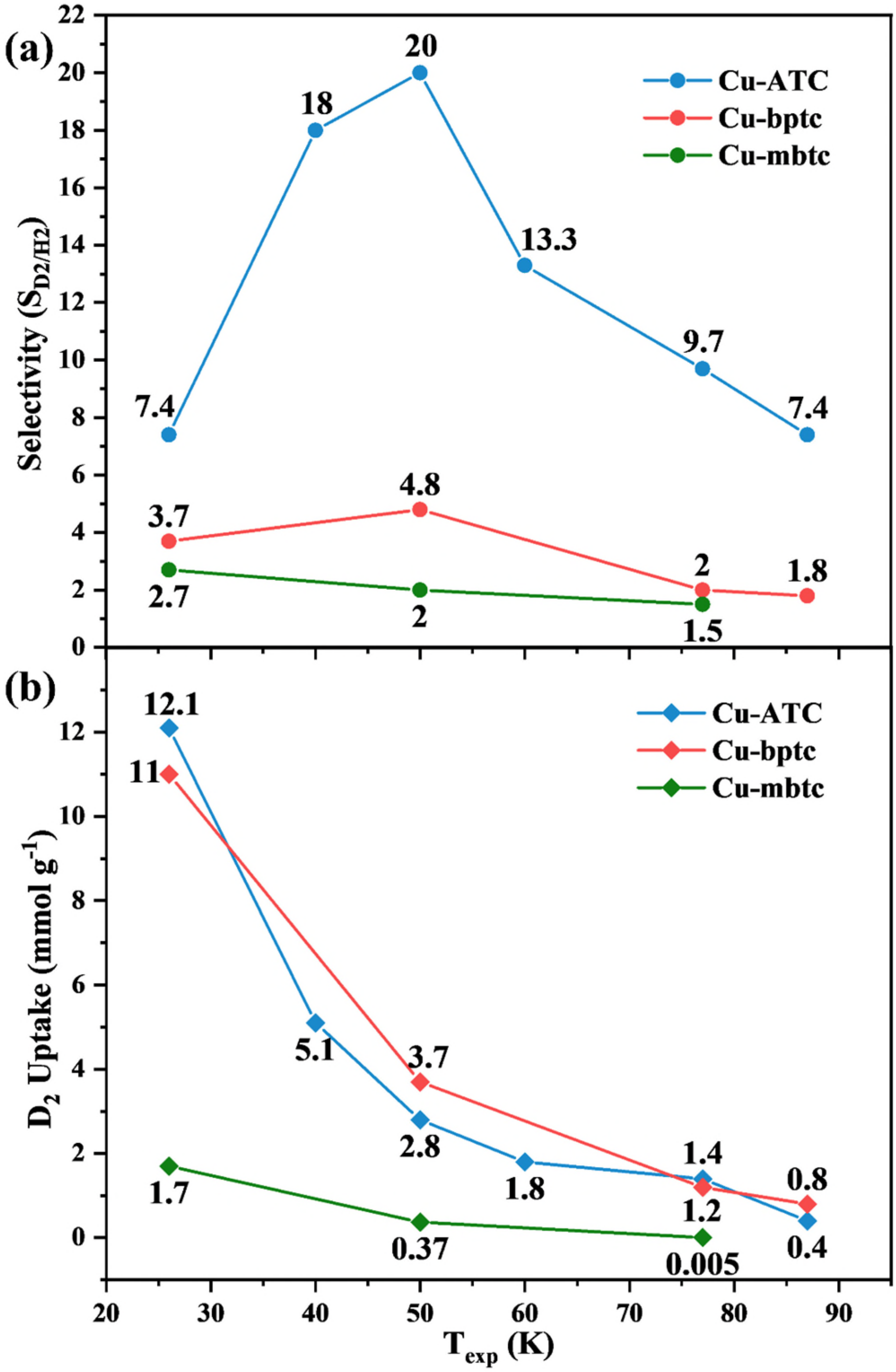

Figure 5. Adsorptive separation for an equimolar D_2/H_2 isotope mixture. (a) Selectivity for D_2 as compared to H_2 and (b) D_2 uptake of Cu-ATC (blue), Cu-bptc (red), and Cu-mbtc (green) as a function of T_{exp}.

5.Comparison to literature in Figure 6: For a proper comparison, the authors should give the references for the different materials. Here it would be important if for all the literature experiments the conditions of exposure pressure and time as well as evacuation temperature are comparable. In contrast, the authors state: "These findings underscore Cu-ATC's CAQS potential above liquid nitrogen temperatures, supporting its promise for industrial hydrogen isotope separation (Figure 6).", I cannot see this promise.

Answer: We sincerely appreciated the reviewer's valuable suggestions. In response, we have added the corresponding references for all comparison materials and summarized the relevant experimental parameters, including the TDS exposure pressure (P_{exp}) and exposure time (t_{exp}) for each material in the Supporting Information？. For clarity and convenience, the data obtained at exposure temperatures comparable to those used in our work have been highlighted in bold in the table, and the relevant references for each material were also provided in the Supporting Information.

We sincerely thank the reviewer for this valuable comment and for pointing out the need for a more careful formulation of this statement. We appreciate the reviewer's caution regarding the practical implications of the present results. Our intention here was to emphasize that Cu-ATC exhibits measurable H_2/D_2 separation performance at 77 K, a temperature readily accessible with liquid nitrogen, which makes this material worthy of further attention in the context of hydrogen isotope separation. Following the reviewer's suggestion, we have revised the relevant statement in the manuscript to present this point in a more precise and balanced manner.

In Revised Manuscript on Page 14.

From:

"These findings underscore Cu-ATC's CAQS potential above liquid nitrogen temperatures, supporting its promise for industrial hydrogen isotope separation (Figure 6)."

To:

“These findings highlight the potential of Cu-ATC for H_2/D_2 separation at liquid-nitrogen-accessible temperatures (Figure 6), while further evaluation under practical process conditions will be required to assess its applicability.”

In Revised Manuscript on Page 17.

From:

“Although dynamic values were lower than those from static isotherms, owing to incomplete equilibration under flow, Cu-ATC’s performance corroborates its industrial potential, consistent with its TDS selectivity (20 at 50 K).”

To:

“Although dynamic values were lower than those from static isotherms, owing to incomplete equilibration under flow, the superior dynamic selectivity and TDS selectivity (20 at 50 K) of Cu-ATC suggested its potential for further applied research.”

Nature Communications manuscript NCOMMS-25-81623B Point-by-point response

We thank all reviewers for their valuable comments helping to improve the manuscript!

Reviewer #1 (Remarks to the Author):

The revised manuscript is improved. The newly added D2 DRIFTS results, the paired-site versus monomer DFT comparison, and the expanded methodological descriptions together provide a reasonably coherent basis for the proposed cooperative dual-site interpretation, even though the evidence remains indirect.

However, a few points still need correction :
(i) the D2 red-shift values in the DRIFTS discussion should be checked carefully for consistency with the figure/table values;

Answer: We sincerely thank the reviewer for this helpful reminder. We carefully rechecked the D_2 adsorption band positions and the corresponding red-shift values in the DRIFTS discussion against the values shown in Figure 3 and Table 1. The red shifts were recalculated using the gas-phase D_2 vibrational frequency of 2993 cm^{-1}. Accordingly, the bands at 2846 cm^{-1} for Cu-ATC and 2888 cm^{-1} for Cu-

bptc correspond to red shifts of 147 and 105 cm^{-1}, respectively. We have corrected these values in the revised manuscript.

In Revised Manuscript Page 12, L191.

From:

“the corresponding red shifts are 169 cm^{-1} for Cu-ATC and 147 cm^{-1} for Cu-bptc, following the expected isotopic trend.”

To:

“the corresponding red shifts are 147 cm^{-1} for Cu-ATC and 105 cm^{-1} for Cu-bptc, following the expected isotopic trend.”

(ii) it still does not fully explain why the selectivity reaches a distinct maximum specifically at 50 K. Since this is one of the most notable features of the data, the discussion should be expanded so that the reader can better understand the physical origin of this non-monotonic behavior.

Answer: We sincerely thank the reviewer for this insightful comment. We agree that the non-monotonic temperature dependence of the TDS selectivity, which increases from 26 K to 50 K and then decreases above 50 K, is one of the key features of our data. We have therefore expanded the discussion in the revised manuscript to clarify its physical origin.

As shown in Figure 5, the D_2/H_2 selectivity of Cu-ATC exhibited a non-monotonic trend: it first increased from 26 K to 50 K and then decreased above 50 K. This phenomenon can be understood as arising from the combined effects of weak binding sites (WBSs), strong binding sites (SBSs), and molecular thermal motion.

At low exposure temperature (T_{exp}), such as 26 K and 40 K, KQS effects were more effective. However, WBSs such as oxygen atoms were also able to adsorb H_2 and D_2, but showed only limited D_2/H_2 discrimination ***[J. Am. Chem. Soc. 2017, 139, 42, 15135–15141]***, resulting in low selectivity.

As the T_{exp} increased from 26 K toward 50 K, molecular thermal motion increased, and adsorption at WBSs gradually decreased. The influence of the SBSs, such as paired Cu(II) OMSs, gradually became more significant, leading to enhanced D_2/H_2 selectivity ***[J. Am. Chem. Soc. 2017, 139, 42, 15135–15141]***.

At around 50 K, the contribution from WBSs was sufficiently reduced, while paired Cu(II) OMSs could still preferentially adsorb D_2 over H_2. This gave rise to the apparent maximum in TDS selectivity at 50 K.

When the T_{exp} was further increased above 50 K (60–87 K), molecular thermal motion became even stronger, the preferential occupation of

deuterium on the paired Cu(II) OMSs weakened, leading to decreased D_2/H_2 selectivity at higher T_{exp}.

We added a dedicated discussion paragraph in the revised manuscript to clearly explain this phenomenon.

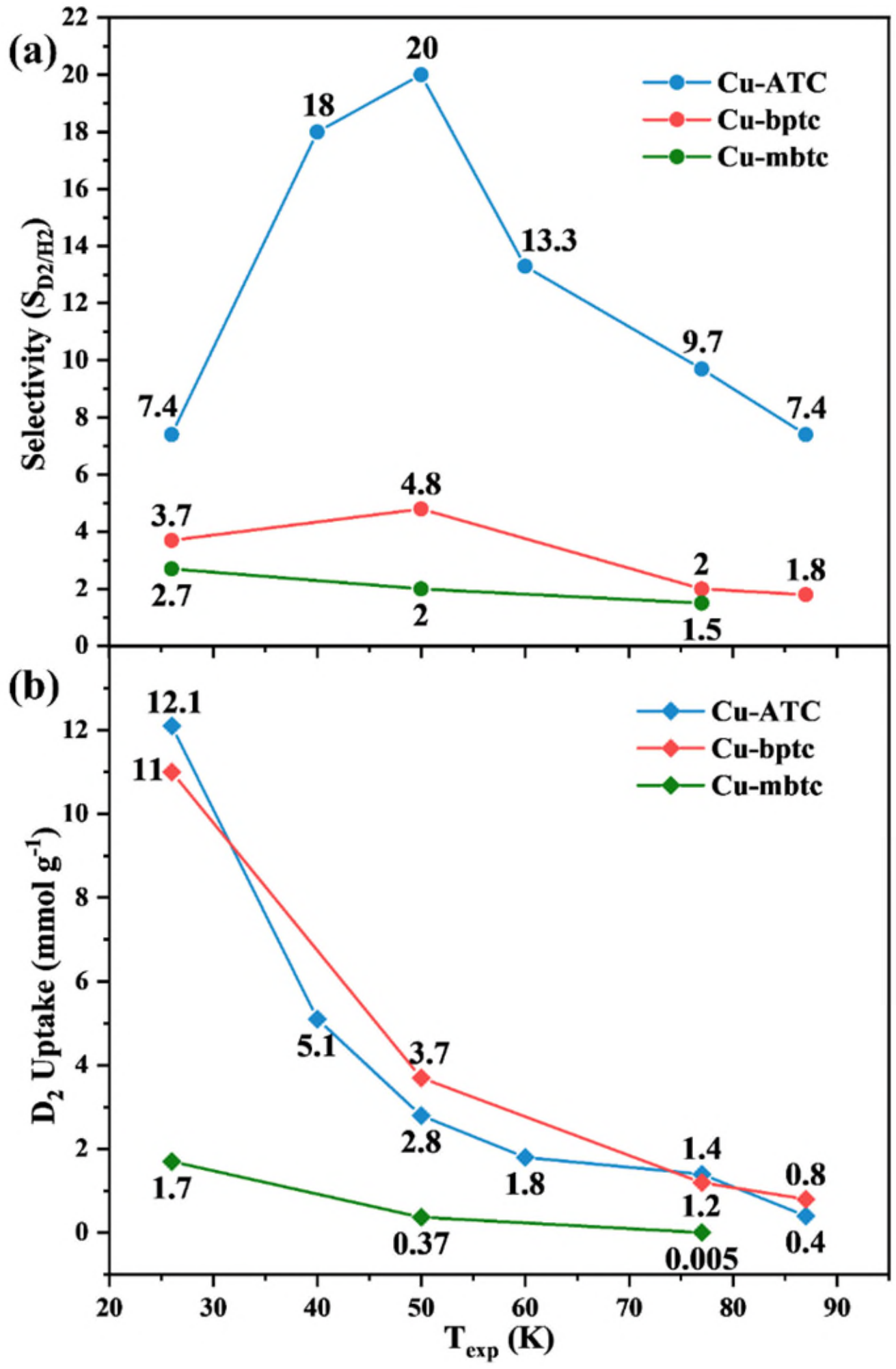

Figure 5. Adsorptive separation for an equimolar D_2/H_2 isotope mixture. (a) Selectivity for D_2 as compared to H_2 and (b) D_2 uptake of Cu-ATC (blue), Cu-bptc (red), and Cu-mbtc (green) as a function of T_{exp}.

In Revised Manuscript on Page 14-15, L229-243.

Added:

The selectivity of Cu-ATC first increased from 26 K to 50 K and then decreased above 50 K, indicating a non-monotonic dependence on exposure temperature. This phenomenon can be understood as arising from the combined effects of weak binding sites (WBSs), strong binding sites (SBSs), and molecular thermal motion ***[J. Am. Chem. Soc. 2017, 139, 15135−15141]***. Below 50 K, WBSs, such as oxygen atoms, also contributed significantly to D_2/H_2 uptake. Because these WBSs showed only limited D_2/H_2 discrimination, their contribution led to relatively low selectivity. As the exposure temperature (T_{exp}) increased toward 50 K, adsorption at WBSs was gradually reduced, while the SBSs, such as paired Cu(II) OMSs, remained capable of preferentially adsorbing D_2 over H_2. As a result, the D_2/H_2 selectivity reached its maximum at 50 K. When T_{exp} increased above 50 K, molecular thermal motion became stronger, and the preferential adsorption of D_2 at the paired Cu(II) OMSs was weakened; consequently, the D_2/H_2 selectivity declined.

(iii) the literature comparison should be softened because the compared data were obtained under non-identical conditions.

Answer: We sincerely thank the reviewer for the suggestion. We agree that the literature comparison should be interpreted cautiously because the reported TDS data were obtained under non-identical experimental conditions. Therefore, we have revised the relevant descriptions to present the comparison for general comparison rather than a strict one-to-one performance ranking. The literature comparisons in the separation performance and conclusions sections have been softened, as shown below.

In Revised Manuscript on Page 14, L228-229.

From:

"whereas Cu-ATC exhibited superior values: 20 at 50 K (the highest among tested Cu(II)-MOFs) and 7.4 at 87 K."

To:

"whereas Cu-ATC exhibited relatively high D_2/H_2 selectivity of 20 at 50 K and 7.4 at 87 K."

In Revised Manuscript on Page 22, L333-335.

From:

"Cu-ATC exhibits steep adsorption isotherms at low pressures and a TDS selectivity of 20 at 50 K, the highest among reported Cu(II)-MOFs."

To:

“Cu-ATC exhibits steep adsorption isotherms at low pressures and a D_2/H_2 TDS selectivity of 20 at 50 K, representing a relatively high value among reported Cu(II)-MOFs, although these literature data were obtained under non-identical experimental conditions.

Reviewer #2 (Remarks to the Author):

It can be accepted.

Answer: We greatly appreciated the reviewer's efforts to help us improve the manuscript. In addition, following the suggestion of Reviewer #4, we corrected the inaccurate parts in our answers to questions 3) and 4).

Reviewer #3 (Remarks to the Author):

The authors have made satisfactory revisions to the manuscript and addressed the concerns I raised. I have no further comments and recommend acceptance of this article for publication in Nature Communications.

Answer: We greatly appreciated the reviewer's efforts to help us improve the manuscript.

Reviewer #4 (Remarks to the Author):

The revised manuscript on Synergistic Effect of Paired Cu(II) Open Metal Sites for Enhanced High-Temperature Hydrogen Isotope Separation by Fenglei Wang et al. has improved in quality and clarity. Most of the reviewers' questions have been satisfactorily answered and additional information is given.

Since the response to reviewers is typically published, the authors' answers should be correct.

1) Reviewer #2's question 3) is answered by "The difference in zero-point energy between T2 and D2 is greater than that between H2 and D2 [Nature Communications 8, 14496]." The difference of zero point energy depends on the mass difference between the isotopes, therefore, H2/D2 = 2/4 and D2/T2 = 4/6 and I cannot find this statement in the reference given.

Answer: We sincerely thank the reviewer for carefully checking this statement and for giving us the opportunity to correct it. We agree that our previous statement comparing the zero-point-energy difference between T_2/D_2 and H_2/D_2 was not accurate and was not directly supported by the cited reference. We have therefore removed this statement and revised the response accordingly.

As the isotopic mass of hydrogen molecules increases, their zero-point energy decreases. At strong adsorption sites in porous materials, the lower zero-point energy of heavier isotope can result in a stronger adsorption. Therefore, heavier isotope such as D_2 and T_2 may be preferentially adsorbed relative to H_2 at strong adsorption sites. Predictive results based on the literature ***[Nature Communications 8, 14496]***, together with experimental findings ***[Chemical Engineering Journal 493 (2024) 152673]***, support the general relevance of quantum sieving effects in hydrogen isotope adsorption and suggest that materials showing H_2/D_2 separation may also be worth evaluating for H_2/T_2 and D_2/T_2 separation.

2) Reviewer #2's question 4) is answered by "As generally observed, D2 consistently desorbed earlier than H2 as the temperature increased [Science 366, 613–620 (2019); Adv. Mater. 2019, 31, 1805293]." Typically, all TDS experiments show H2 desorption maxima at lower temperature compared to D2, again I cannot find this argument shown in the two references.

Answer: We sincerely thank the reviewer for carefully checking this statement and for giving us the opportunity to correct it. We agree that our previous statement was inaccurate and potentially ambiguous; the

phrase "desorbed earlier" could be interpreted as referring to the desorption maximum, whereas our intention was to discuss early desorption features under specific TDS conditions. Therefore, we have removed the unsupported general statement that "D_2 consistently desorbed earlier than H_2." from our response to Reviewer #2's question 4. We have therefore revised the response accordingly.

Based on our literature survey, the relative desorption behavior of H_2 and D_2 is not universal and can vary depending on the material, adsorption environment, and TDS testing conditions. Different cases have been observed, including: 1) H_2 desorb earlier than D_2 ***[ACS Nano 2014, 8, 1, 761–770, J. Am. Chem. Soc. 2017, 139, 15135−15141]***(reviewer's only figure 4a); 2) H_2 and D_2 desorbed simultaneously ***[Nature Communications | (2025) 16:5941]*** (reviewer's only figure 4b); 3) D_2 desorbed earlier than H_2 ***[Science 366, 613–620 (2019), Nature Communications | 8:14496]***(reviewer's only figure 4c). In this study, for Cu-ATC, at exposure temperature of 26 K (figure 4a), H_2 showed a slightly earlier desorption onset than D_2, likely associated with weak binding sites that provide limited isotope discrimination ***[J. Am. Chem. Soc. 2017, 139, 15135−15141]***; in contrast, D_2 was more strongly retained at stronger adsorption environments and therefore required a higher temperature for desorption.

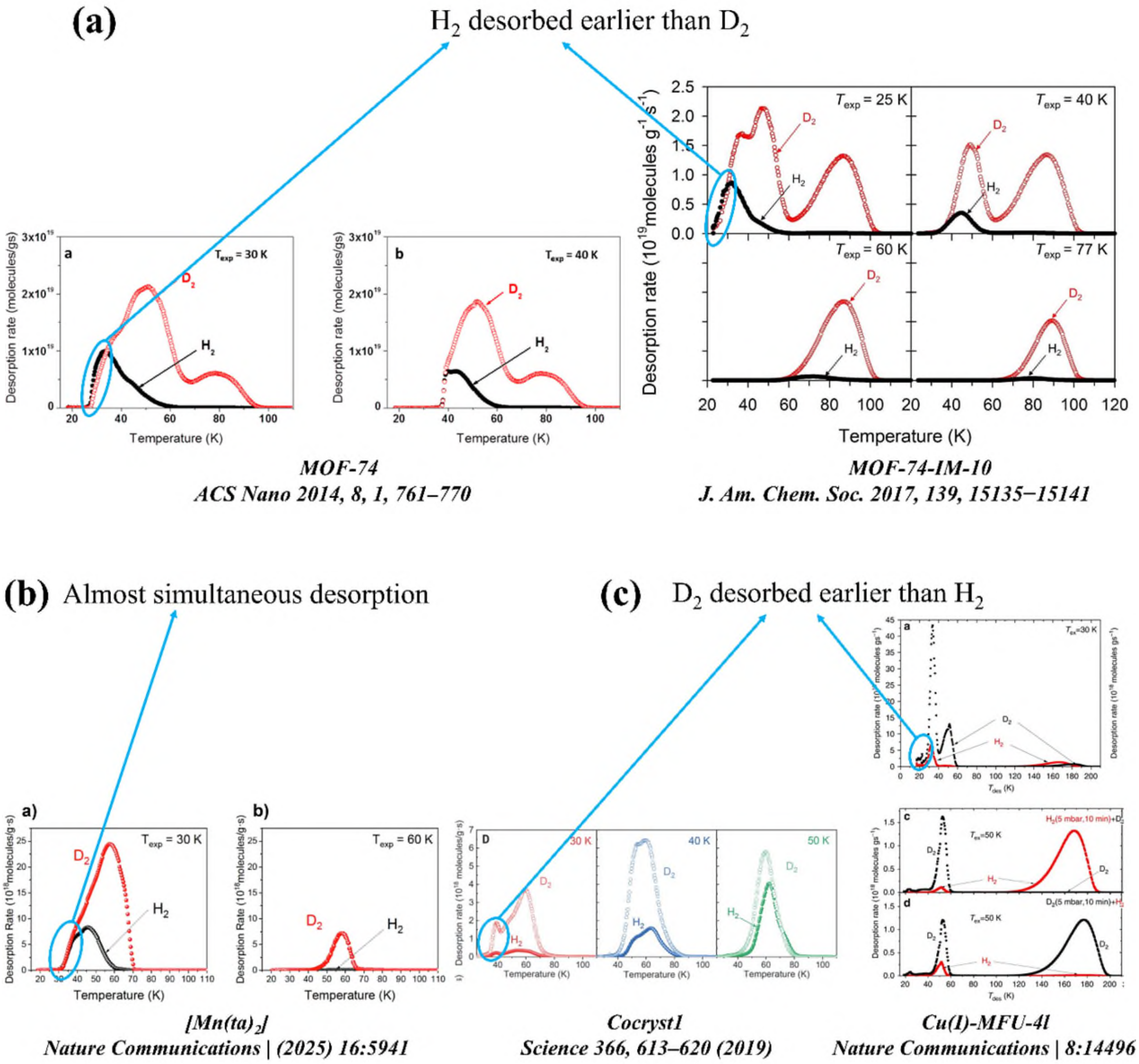

Reviewer’s only figure 4.

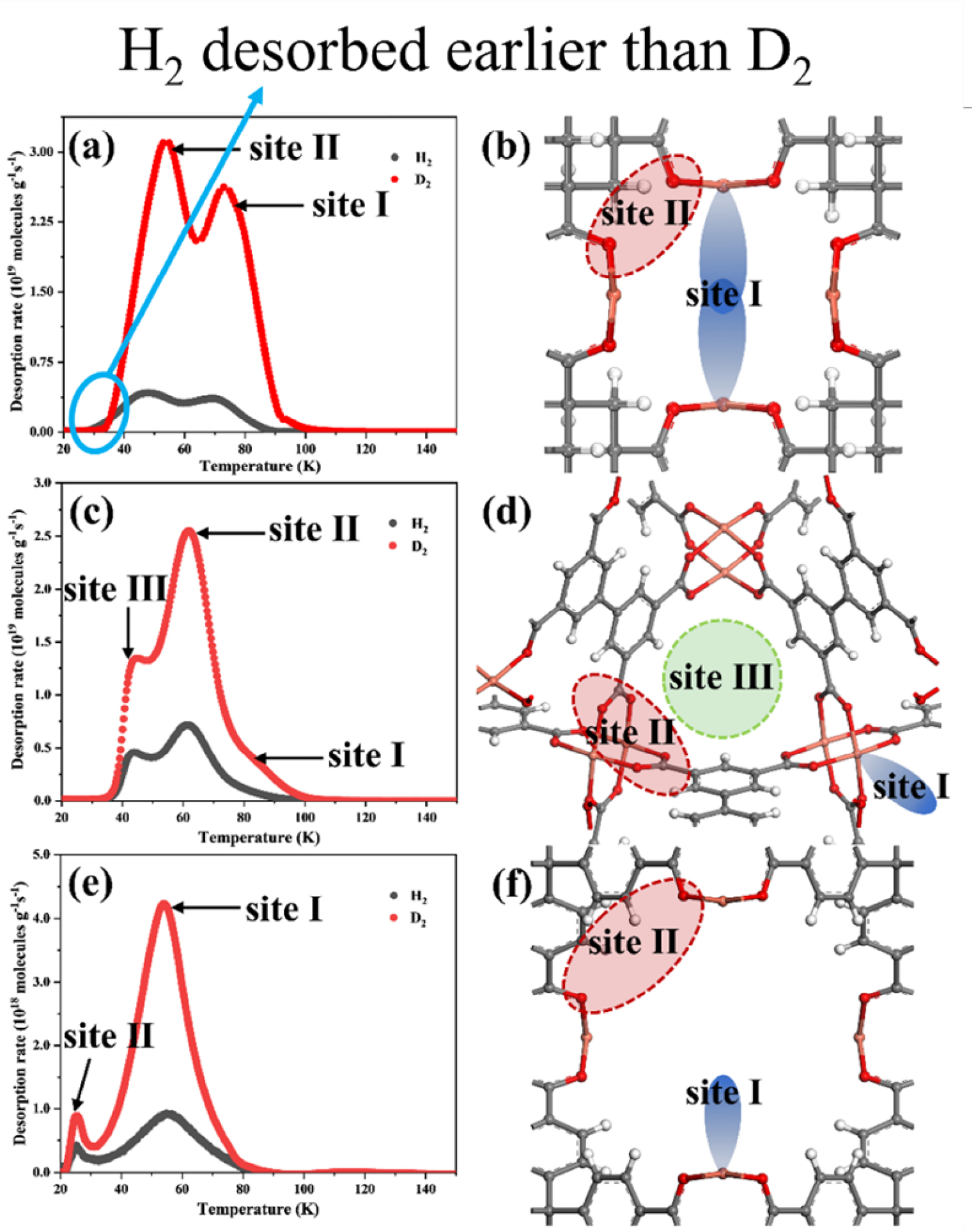

Figure 4

This work

Nature Communications manuscript NCOMMS-25-81623C

Point-by-point response

We thank all reviewers for their valuable comments helping to improve the manuscript!

Reviewer #1 (Remarks to the Author):

Now the authors have responded to all my concerns.

Answer: We greatly appreciated the reviewer's efforts to help us improve the manuscript.

Reviewer #4 (Remarks to the Author):

The second revision of the manuscript on Synergistic Effect of Paired Cu(II) Open Metal Sites for Enhanced High-Temperature Hydrogen Isotope Separation by Fenglei Wang et al. has improved in quality and clarity, again.

However, authors' response to the reviewer's comment 2): "Reviewer #2's question 4) is answered by "As generally observed, D_2 consistently desorbed earlier than H_2 as the

temperature increased [Science 366, 613–620 (2019); Adv. Mater. 2019, 31, 1805293].” Typically, all TDS experiments show H_2 *desorption maxima at lower temperature compared to* D_2*, again I cannot find this argument shown in the two references.”*

The authors' new answer is really a bit strange, since now the authors are no longer considering the main desorption maxima, but look at the tiny initial start of desorption. This little initial desorption is only around 1 % of the total desorption and 98 % are in the maxima which show the typical behavior first H_2 *than* D_2 *desorption. Assigning 1 % of the total adsorbed amount to a specific adsorption site is simply impossible, therefore, such an analysis is unnecessary and doesn't add any additional information to the paper. Furthermore, this very minor effect occurs at temperatures below 30 K, here the higher boiling point of* D_2 *compared to* H_2 *may cause this small prior desorption, by a very tiny amount of condensed* D_2 *in pores or inter-particle voids.*

Answer： We sincerely thank the reviewer for this careful and constructive clarification. We agree that our previous response placed unnecessary emphasis on the very small initial desorption feature below 30 K. Since this feature accounts for only a minor fraction of the total desorption, it should not be used for

mechanistic interpretation or adsorption-site assignment.

Accordingly, we have removed the onset-based discussion and no longer use this minor feature to support the adsorption-site assignment or the separation mechanism. In the revised response, we focus on the main desorption maxima, which represent the dominant desorption behavior. The main TDS maxima show the expected trend, with H_2 desorption maxima occurring at lower temperatures than D_2 consistent with the stronger adsorption of D_2 at stronger adsorption sites.

We also agree with the reviewer that the anomalous desorption onset might have been caused by the different boiling points of H_2 and D_2 or by a very tiny amount of condensed D_2 in pores or inter-particle voids, rather than from a well-defined adsorption site. Therefore, this minor feature is no longer discussed as mechanistic evidence. We have removed the unsupported statement that "D_2 consistently desorbed earlier than H_2" and revised the response to avoid ambiguity between desorption onset and desorption maxima.

We greatly appreciate the reviewer's careful assessment, which helped us correct the wording and avoid overinterpretation of a minor feature. We have revised our response to Reviewer #2's

question 4) as follows.

Based on our literature survey, because H_2 binds weakly to the MOF than D_2, typically all TDS experiments show H_2 desorption maxima at lower temperatures compared to D_2. The anomalous desorption onset might have been caused by the different boiling points of H_2 and D_2 or by a very tiny amount of condensed D_2 in pores or inter-particle voids.
